# Photoactive nanomaterials enabled integrated photo-rechargeable batteries

**DOI:** 10.1515/nanoph-2021-0782

**Published:** 2022-04-06

**Authors:** Cristina Rodríguez-Seco, Yue-Sheng Wang, Karim Zaghib, Dongling Ma

**Affiliations:** Institut National de la Recherche Scientifique (INRS)-Centre Énergie Materiaux et Telécommunications, 1650 Boulevard Lionel-Boulet, Varennes J3X 1P7, Québec, Canada; Center of Excellence in Transportation Electrification and Energy Storage, Hydro Québec, 1806 Boulevard Lionel-Boulet, Varennes J3X 1S1, Québec, Canada; Department of Mining and Materials Engineering, McGill University, Montréal QC H3A 0C5, Canada

**Keywords:** energy storage systems, morphology, nanomaterials, photoactive materials, photobatteries, photocatalysis

## Abstract

The research interest in energy storage systems (*e.g.* batteries and capacitors) has been increasing over the last years. The rising need for electricity storage and overcoming the intermittent nature of renewable energy sources have been potent drivers of this increase. Solar energy is the most abundant renewable energy source. Thus, the combination of photovoltaic devices with energy storing systems has been pursued as a novel approach in applications such as electric vehicles and smart grids. Among all the possible configurations, the “direct” incorporation of photoactive materials in the storing devices is most attractive because it will enhance efficiency and reduce volume/weight compared to conventional systems comprised two individual devices. By generating and storing electricity in a singular device, integrated photo-rechargeable batteries offer a promising solution by directly storing electricity generated by sunlight during the day and reversibly releasing it at night time. They hold a sizable potential for future commercialization. This review highlights cutting-edge photoactive nanomaterials serving as photoelectrodes in integrated photobatteries. The importance and influence of their structure and morphology and relevant photocatalytic mechanisms will be focal points, being strong influencers of device performance. Different architecture designs and working principles are also included. Finally, challenges and limitations are discussed with the aim of providing an outlook for further improving the performance of integrated devices. We hope this up-to-date, in-depth review will act as a guide and attract more researchers to this new, challenging field, which has a bright application prospect.

## 1 Introduction

The development of human society is causing an unprecedented increase in energy demand [1]. The limited supply of fossil fuels and the increasingly serious environmental pollution have become major global problems. In order to address these issues, it has become crucial for the scientific community to explore sustainable energy sources, including hydroelectric power [2], wind [3], solar [4], [5], [6], ocean [7], geothermal [8], and biomass [9] energy. Because of its unlimited nature and sustainability, solar energy is recognized as the most promising and economic alternative to traditional fossil fuels [10, 11]. The use of solar cells as an energy conversion technology can effectively meet our long-term needs [12, 13]. However, this process is dependent on the availability of energy sources and is not always in alignment with actual demand [14, 15]. Several solar energy conversion and storage devices have been proposed as solutions. These include rechargeable (secondary) batteries (*e.g.* lithium-ion, lithium–oxygen, lithium–sulfur batteries, zinc-ion, redox flow batteries, etc.) [16], [17], [18], [19], [20] and supercapacitors [21], [22], [23] coupled with a solar cell [24], [25], [26], [27]. Some of these devices are currently being used in a variety of fields such as electrical vehicles and portable electronic devices, among others [28, 29].

One obvious solution lies in the combination of a photovoltaic cell (silicon, dye-sensitized or perovskite solar cells) with an external electrochemical device (*e.g.* rechargeable battery or capacitor). The former acts as an energy harvester whereas the latter stores electricity externally in the form of chemical energy. These two individual systems are linked via external wire connection [30, 31]. However, they suffer from increased Ohmic resistance, energy mismatch, and low levels of integration between the two systems, which leads to bulky, inflexible devices and high energy losses [30, 32, 33].

An attractive alternative to devices relying on discrete modules and external wires connection is an integrated photo-rechargeable energy storage system [34], [35], [36], [37]. Photobatteries (PBATs) [31, 37, 38], photocapacitors (PCAPs) [36, 39], [40], [41], and redox flow batteries (RFBs) [42], [43], [44] are included in this category. The advantages of integrated systems are their flexibility, light weight, high safety and smaller volume compared to the wired systems [30, 45]. They can be readily implemented in wearable electronics, implanted biosensors and smart optoelectronics, widening the application fields [46], [47], [48]. However, these devices do not allow practical applications due to their low performance to date.

The most commonly used batteries are based on a metal anode, with lithium, zinc, and sodium being the most representative examples of anode materials. Among them, lithium-based materials are most studied and used as anode nowadays in solar rechargeable batteries [14, 37, 45, 49]. The major advantage of using lithium is its large theoretical energy density (3560 W h kg^−1^) [50]. In addition, because of its small size, lithium ions can easily intercalate into other nanomaterials with a high insertion/removal rate, without modifying their structure (*i.e.* MnO_2_) [51]. However, Li-based PBATs present a major drawback related with the significantly large overpotential (4–4.5 V) during the charge process, which leads to high voltage gaps and low round-trip efficiencies [52], [53], [54]. Particularly, the degradation of Li–O_2_ PBATs is linked to the incomplete dissolution of Li_2_O_2_ even at high voltages and the accumulation of parasitic products in the cathode, which ultimately causes decaying in the overall capacity over cycles [55, 56]. Zinc has high specific capacity (820 mA h g^−1^ and 5854 mA h cm^−3^), good stability in aqueous electrolytes, low toxicity, high safety, low cost, and it is environmentally friendly [57], [58], [59]. With these properties, it has the potential to become the new energy storage material. In general, metal-based PBATs suffer from slow intrinsic diffusion of metal ions in a solid material, which limits the intercalation rate, and from sluggish reaction kinetics towards oxygen reduction reaction (ORR) and oxygen evolution reaction (OER). Because of these problems, the focus should be on finding a photoactive bifunctional material towards OER and ORR that can also improve the light harvesting capability, reduce charge recombination, increase the surface area to enhance a better contact with the electrolyte and allow a higher metal ion insertion/removal rate [60, 61]. Nanomaterials are a promising solution that offers all the above mentioned qualities [62], [63], [64]. Vanadium oxides with nanowires or nanorods morphology have been proven to be the best candidates to be used as PEs. J. Wang et al., reported a photorechargeable lithium-ion battery using LiV_2_O_5_ as a photocathode with a photo-to-electric conversion efficiency of 9%, the highest value published to date [65]. Other devices based on V_2_O_5_ nanofibers obtained photo-to-electric conversion efficiencies of 2.6% [66] and 1.2% [59] in Zn–air and Li-ion PBATs, respectively. Other nanomaterials that form 2D nanosheets (such as siloxene, SiC and g-C_3_N_4_) have shown high capacities [67], [68], [69]. However, there still remain many challenges to overcome in order to improve the device performance. Low light absorption, high charge recombination, poor charge transport capabilities, and low photo and chemical stability are the major drawbacks preventing higher battery efficiencies. In addition, sluggish kinetics between the nanomaterial and the redox active species in the electrolyte limit their photocatalytic performance [70], [71], [72]. Great efforts are being made to fabricate cost-effective devices with high energy conversion efficiency and good compatibility between the photo-conversion and energy storage components [73].

**Figure 1:**
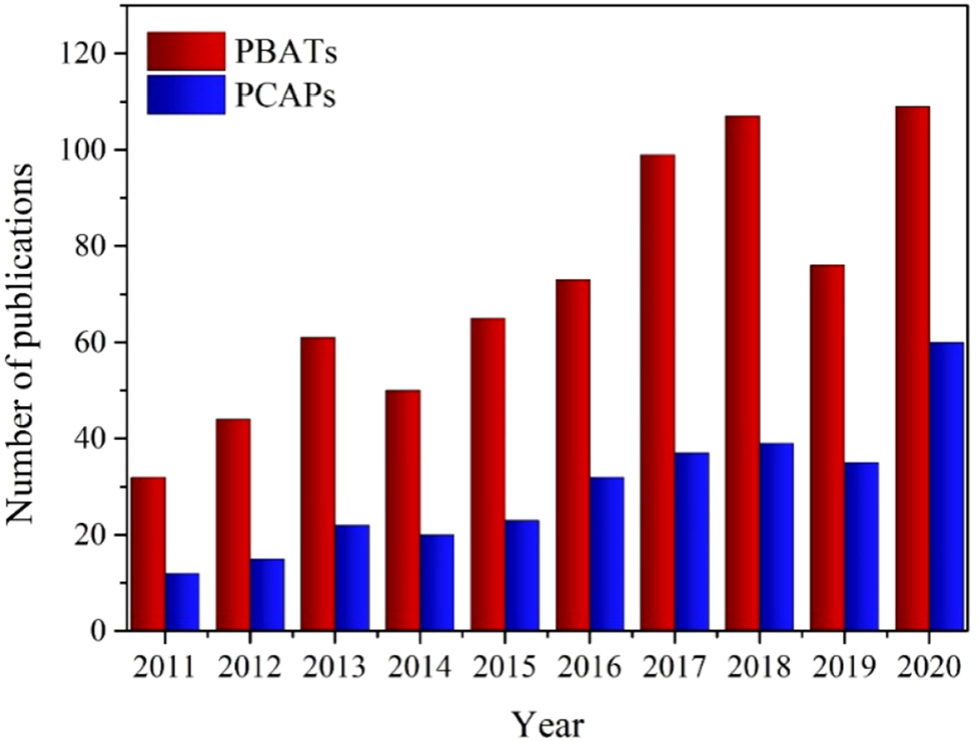
Annual publication number on integrated photobatteries (red) and photocapacitors (blue). Data were retrieved from *Web of Science* on November 09th, 2021, by searching “TS = (solar rechargeable bat* or photo-assisted bat*)” and “TS = (photocapac* or photosupercapac* or photo integrated supercapac*)”.

As seen in Figure 1, the number of publications dealing with integrated photo-rechargeable batteries and photocapacitors has raised exponentially over the last ten years, thanks to the great efforts of many investigators, who have devoted to exploring different strategies, materials and processes in order to achieve highly efficient devices and understand the underlying mechanisms [44, 74]. The accumulated knowledge now enables us to better realize the rational design, which is the key to the rapid development of more efficient energy storage systems. Some excellent reviews dealing with photobatteries from a device performance perspective have been published [14, 38, 43], [44], [45, 75, 76]. However, an up-to-date report of the most relevant photo-active nanomaterials used in photobatteries, from a structural and morphological point of view, has not been done yet.

This review first introduces major configurations and working principles in addition to basic concepts in electrochemistry and energy storage. It is followed by a summary of photo-active nanomaterials incorporated in the photoelectrode (PE), emphasizing the influence of their structure and morphology in the energy harvesting and storing. The most notable photocatalytic mechanisms that govern light-to-electricity energy conversion are also included. Ultimately, a discussion of the challenges and limitations of photoelectrodes in integrated systems is presented along with ideas for rationally designing bifunctional photo-active nanomaterials that serve as photoelectrodes.

## 2 Architecture design, working principles, and basic concepts in photobatteries

Previous reports in the field classify integrated photobatteries according to the number of electrodes (two or three), the physical state of the active materials (solid, liquid, and gas) or the anode materials (*e.g.*, lithium, zinc, and sodium). Here, we describe the photobatteries based on the nature of the photoactive material in the PE. This is a novel point of view that will promote future rational design of PEs in solar-rechargeable batteries. Prior to that, we briefly introduce the different types of configurations, outlining working principles and basic concepts to create a foundation before addressing the topic of interest for this review.

**Figure 2:**
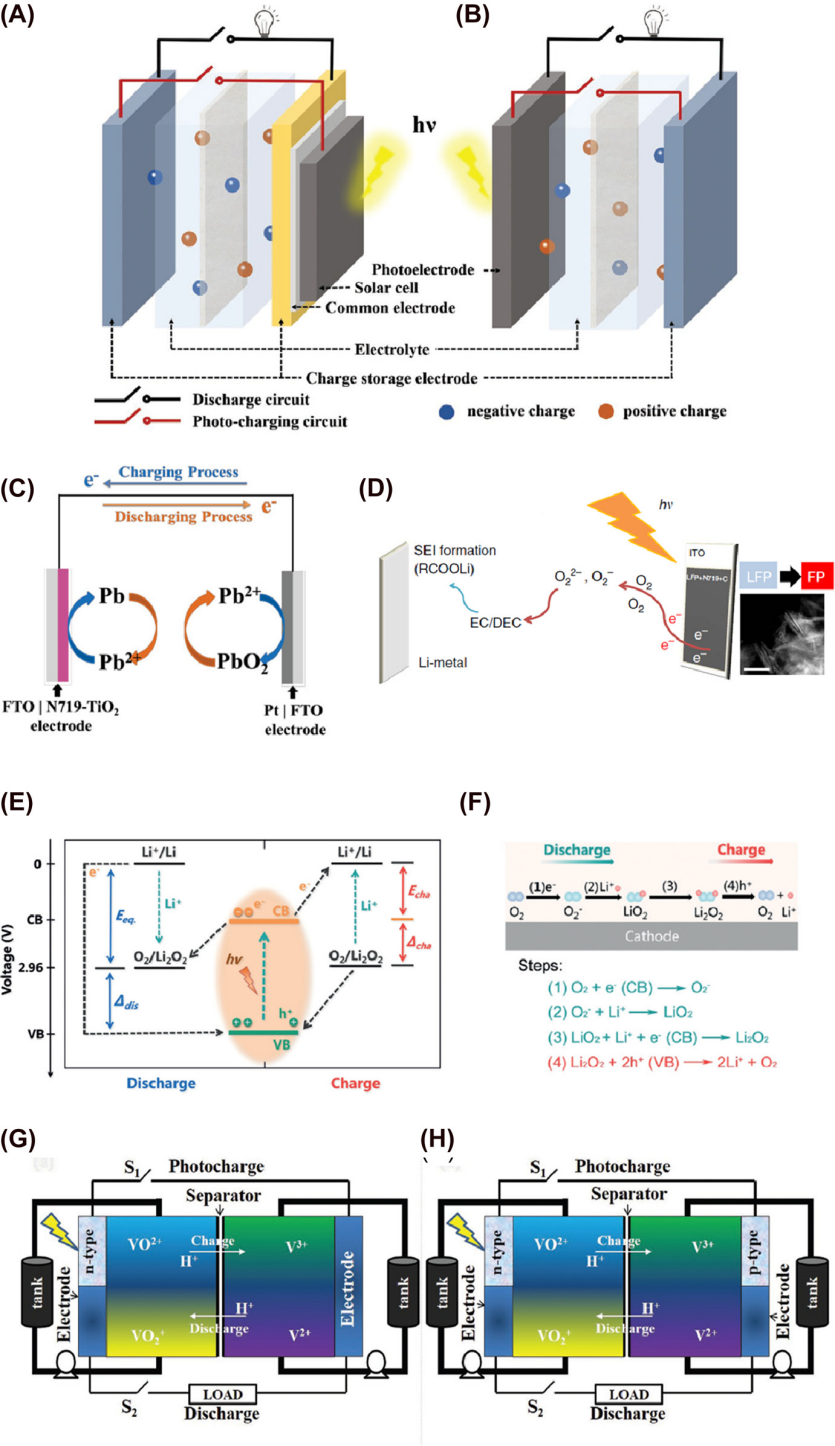
Battery configuration of integrated (A) three-electrode PBAT, (B) two-electrode PBAT [51]. Copyright 2020, John Wiley and Sons; (C) Redox reactions at the electrodes under charging and discharging processes [82]. Copyright 2015, John Wiley and Sons; (D) Global photo-assisted charging mechanism of Li-ion PABT [83]. Copyright 2017, Springer Nature; (E) Energy diagrams for the discharge and charge voltage under illumination; (F) reactions in discharge and charge processes [84]. Copyright 2019, John Wiley and Sons; SFB with (G) single photoelectrode (H) dual photoelectrodes [44]. Copyright 2018, John Wiley and Sons.

The first photo-rechargeable battery was reported by Hodes and co-workers in 1976, where they described a three-electrode system composed of cadmium selenide/sulfur/silver sulfide (CdSe/S/Ag_2_S) [77]. Since then, several reports have been published on the same matter exploring different hybrid photoelectrodes, such as silicon/silicon oxide, dye-sensitized titania (TiO_2_)/poly/3,4-ethylenedioxythiophene, (PEDOT) [78] and dye-sensitized TiO_2_ nanorods [79] (Figure 2A). They usually consist of a photoelectrode (PE), a counter electrode (CE) that works as a redox electron transfer surface, and an energy storage electrode. Under illumination, the photo-active material in the PE creates electron–hole pairs. If the voltage generated is sufficient to activate the electrochemical charging process inside the battery, the electrons travel to the anode where they are either stored or used to reduce the active species. Meanwhile, holes in the PE are counterbalanced by electrons at the CE or by redox species that will be reduced at the CE [45, 76]. If the voltage is insufficient to reduce the active species, an external bias must be applied. In these cases, we say that the device is a photo-assisted rechargeable battery. It helps to reduce the overpotential, but these devices cannot be charged solely with solar energy.

On the other hand, two-electrode integrated PBATs allow direct conversion and storage of the solar energy without any additional energy source [30, 80]. They consist of a photoanode and a photocathode where one or both can contribute to energy conversion as well as to energy storage [35, 74, 76]. Under illumination, the photoactive material in the PE (a semiconductor) generates photoexcited carriers (electrons and holes) through the photovoltaic effect. They interact with redox-active species from the energy storing electrode and reduce them with or without external electrical bias [38, 49]. The holes can oxidize other redox active species or be engaged in OERs. A schematic diagram of two and three photo-assisted rechargeable battery configurations are shown in Figure 2A and B.

In the 1980s, we can find the first publications reporting two-electrode configurations. For example, Sharon et al. developed a two-redox-electrolytes system divided into two compartments based on BaTiO_3_|Ce^4+/3+^|Fe^3+/2+^|Pt. Ce^3+^ was oxidized to Ce^4+^ on the surface of the PE and Fe^3+^ reduced to Fe^2+^ on the counter electrode in the photocharging process [81]. Other examples of two-electrode integrated systems are reported in more recent publications such as the one by Wang et al. in 2015, where they reported a photo-assisted rechargeable battery based on a dye-sensitized solar cell (DSSC) with a lead-organohalide electrolyte (CH_3_NH_3_I·PbCl_2_). In this case, Pb^2+^ is reduced into Pb at the interface of dye–TiO_2_ PE with photogenerated electrons from the dye. Meanwhile, Pb^2+^ is oxidized into PbO_2_ at the Pt counter electrode during charging step (Figure 2C). During the discharge, electrons return to the Pt electrode from the dye–TiO_2_ PE when Pb is oxidized to Pb^2+^. The overall reactions of charging and discharging are summarized in Eq. (1) [82]. Paolella et al. introduced a two-electrode system involving direct photo-oxidation of lithium iron phosphate (LiFePO_4_; LPF) nanocrystals in the presence of dye N719 at the photocathode. Figure 2D shows the photocharging mechanism. Photogenerated holes in the dye aid the delithiation of LFP and photogenerated electrons reduce oxygen (O_2_) into peroxide and/or superoxide species that will react with the carbonate-based electrolyte. Li electrode enabled favourable nucleation sites for the formation of a solid electrolyte interface (SEI) formed by Li-carbonate-based compounds (RCOOLi) [83]. In this case, no external current is applied between the electrodes during illumination and contrary to what it is expected in a regular Li-ion PBAT, lithium is not reduced and deposited on the lithium anode. During discharge, SEI crystals partially dissolved, releasing Li^+^ ions that travel to the cathode to form LPF (see Eqs. (2) and (3)). In 2019, Zhuo et al. reported an aprotic Li–O_2_ PBAT using C_3_N_4_ as a photocatalyst in the photocathode [84]. The photochemical mechanism is explained in Figure 2E and F. During charge, C_3_N_4_ captures light, leading to photogenerated electrons and holes at the conduction band and valence band, respectively. Holes promote the decomposition of Li_2_O_2_ into Li^+^, which will be reduced to Li at the anode by the photogenerated electrons (see Eqs. (4) and (5)). Therefore, the charging voltage is reduced up to 3.38 V. During discharge, O_2_ is reduced to O_2_^−^ and O_2_^2−^ in the cathode followed by its reaction with Li^+^ ions present in the electrolyte to form Li_2_O_2_ (Figure 2F). The value of discharge voltage under illumination (3.22 V) is related to the energy difference between C_3_N_4_ valence band and Li^+^/Li redox potential, and it is higher than that of the equilibrium (2.96 V), indicating the beneficial conversion of solar energy into electricity. Likewise, the charge voltage is greatly reduced (from 4.09 to 3.38 V) in the presence of light.

(1) $$2{\text{Pb}}^{2+}\left(\text{aq}\right)+2{\text{H}}_{2}\text{O}\left(\text{aq}\right)⇆{\text{PbO}}_{2}\left(\text{s}\right)+\text{Pb}\left(\text{s}\right)+4{\text{H}}^{+}\left(\text{aq}\right)$$

(2) $${\text{LiFePO}}_{4}+\text{dye*}+{\text{O}}_{2}\to {\text{FePO}}_{4}+{\text{Li}}^{+}+\text{dye}+{\text{O}}_{2}^{-}$$

(3) $${\text{FePO}}_{4}+{\text{e}}^{-}+{\text{Li}}^{+}\to {\text{LiFePO}}_{4}$$

(4) $${\text{Li}}^{+}+{\text{e}}^{-}\left({\text{C}}_{3}{\text{N}}_{4}\right)⇆\text{Li}\left(\text{anode}\right)$$

(5) $${\text{Li}}_{2}{\text{O}}_{2}+{\text{h}}^{+}\left({\text{C}}_{3}{\text{N}}_{4}\right)⇆2{\text{Li}}^{+}+{\text{O}}_{2}$$

A general idea of the mechanism of a PBAT has been described as well as some relevant examples. However, due to the complexity of the systems, especially the differences on the PE materials and device architecture, the mechanism of each system should be studied individually. The photoactive material at the PE plays a key role in the PBAT mechanism. Its structure, morphology, energy levels and the reaction kinetics between the redox active species and the photogenerated electrons and holes at the PE will highly influence the performance and stability of the PBAT. For instance, BiVO_4_ (BVO) PE has been studied and compared to Fe_2_O_3_ PE to assess the effect of the band structure on LiO_2_ and Zn–air PBATs [85, 86]. BVO presented severe photocorrosion and lost its photocatalytic activity soon after starting photocharging. Under the same conditions, Fe_2_O_3_ PE showed high stability over up to 41 cycles.

RFBs store electrical energy via two liquid redox-couple electrolytes called anolyte and catholyte. The electrolytes are separated by an ion-selective membrane and can be stored in external reservoirs of different volumes. This allows scaling up the energy storage capacity by using larger electrolyte tanks. Due to direct electrode contact with the liquid electrolyte, the working temperature is lower compared to its “solid” counterparts, which grants RFBs higher stability and durability [42, 43, 87, 88]. This is extremely important in solar integrated devices since exposure to sunlight will raise solution temperature, which is deleterious for optimal battery functioning. Integrated solar flow batteries (SFBs) combine a solar energy conversion system with a rechargeable RFB. One or two PEs enable the photocharging process. The working principles are very similar to those found in two and three-electrode systems. In the PE, the generated electron–hole pairs interact with the anolyte, converting the solar energy into chemical energy that will be stored directly in the tanks (see Figure 2G and H). Holes diffuse to the cathode and oxidize the catholyte while electrons move to the anode and reduce the anolyte. The internal charge is balanced by ion species that travel through the membrane or separator [44, 74]. The first publication integrating a photovoltaic system (DSSCs) with nonaqueous redox couples obtained currents of 0.1 mA/cm^2^ and efficiencies lower than 0.1% [89, 90]. Since that time, solar-to-output electric efficiencies up to 20.1% [91], [92], [93] have been achieved through fabrication of single-junction silicon photoelectrodes, perovskite/silicon tandem solar cells, or single-junction GaAs photoanode.

The overall energy efficiency is an important parameter of any integrated energy conversion and storage device, including rechargeable battery systems and directly reflect how effectively the device can convert one type of energy to another (typically between electric to chemical energy). Specifically, for solar rechargeable batteries, there are three main processes that determine the overall energy efficiency: solar-to-electric energy conversion, electric-to-chemical energy conversion (photocharging step) and reversible chemical-to-electric energy conversion (discharge step). The electric-to-chemical energy conversion is the stage that limits overall efficiency. The following formula can be used to define photo-to-electric conversion efficiency (*η*_conversion_):

(6) $$\eta_{\text{conversion}}=\frac{E_{\text{output }\left(\text{battery discharging}\right)}}{E_{\text{input }\left(\text{solar energy}\right)}}=\frac{E_{\text{A}}A_{1}}{P_{\text{in}}tA_{2}}$$

where *E*_output (battery discharging)_ and *E*_input (solar energy)_ represent the discharging energy of the battery and the solar energy applied during charging, respectively. *E*_A_, *A*_1_, *P*_in_, *t*, *A*_2_ correspond to the areal energy density, the surface area of the PBAT, the light intensity, the photocharging time and the illuminated surface area [59, 83, 94].

Another highly cited concept is the round-trip efficiency or energy efficiency (*η*_battery_) that is used in photo and non-photo rechargeable batteries. It is the relationship between the output electric energy *E*_output_ and the input electric energy *E*_input_, dependant on the charging and discharging rate [95, 96]. Equation (8) displays Coulombic efficiency (CE). It is the proportion between the capacity during discharge (total charge extracted from the battery) and charge (total charge put into the battery over a full cycle) [97, 98]. In the same way, voltage efficiency (*V*_eff_) in Eq. (9) corresponds to the ratio between the discharge voltage and the charge voltage, either in the dark or under illumination.

(7) $$\eta_{\text{battery}}=\frac{E_{\text{output}}}{E_{\text{input}}}$$

(8) $$\text{CE}=\frac{Q_{\text{discharge}}}{Q_{\text{charge}}}$$

(9) $$V_{\text{eff}}=\frac{V_{\text{discharge}}}{V_{\text{charge}}}$$

## 3 Photoactive nanomaterials working as bifunctional photoelectrodes

PE is one of the key factors in the development of high-performance photobatteries and usually sets the limit of the overall efficiency. Therefore, the design and selection of the materials, composites or heterojunctions that comprise it are essential for efficient conversion and storage of the solar energy [31]. As we previously mentioned, the PE consists of either a simple semiconductor or a photovoltaic cell. In the latter, the PE is part of the energy conversion unit and works independently from the storage system. In this case, it needs to meet the specific requirements for an efficient solar cell. Integrated devices composed of a solar cell that shares an electrode with the battery component are outside the scope of this review. Many publications have already dealt with this topic, including silicon solar cells [93, 99, 100], DSSCs [101], [102], [103], [104], [105], [106], perovskite solar cells (PSCs) [107], [108], [109], [110], and other 3-electrode systems [111] coupled with a regular rechargeable battery. The mechanisms underlying solar and battery systems can also be found elsewhere [31, 45, 112]. In integrated two-electrode photobatteries, we refer to the PE as bifunctional because it must accomplish two purposes: harvest energy from sun light and store it chemically. To ensure light absorption in a wide range of the spectrum, the optimal bandgap of the semiconductor should fall between 1 and 2 eV [113]. In addition, the PE should be photo-, thermo-, and chemically stable within the working voltage range during charge and discharge. This is a critical factor that greatly influences the lifetime of the battery.

Batteries can store energy via two processes: redox reactions and/or ionic transfer electrolytes. Both mechanisms need an efficient charge transport between the PE and the electrolyte. An efficient charge carrier transport is not only conditioned by the energy levels, but also by the morphology, defect concentration and doping level of the semiconductor material in the PE [113]. Additionally, it is desirable that the nanomaterial offers a high specific surface area providing extensive contact with the electrolyte. If the working mechanism involves the insertion of metal ions (Li^+^, Na^+^, K^+^), it should have interstitial sites to reversibly accommodate these ions without deforming or cracking the nanostructure [114].

Finally, the requirements for the band-edge positions vary depending on the type of batteries. For intercalation photobatteries, there should be a trade-off between the CB position of the photoactive semiconductor and the redox reaction to compensate the charge imbalance due to cation intercalation [113]. In the case of Li–O_2_ photobatteries, the redox potential of O_2_/Li_2_O_2_ couple must be positioned between the VB and CB of the semiconductor [84, 115]. On the other hand, RFBs batteries have a more complex system with different redox active species. The electron acceptor redox level should be lower than that of the semiconductor’s CB and the VB of the semiconductor should be placed lower than the electron donor level [116]. However, the VB cannot be too positive in order to avoid solvent oxidation on the PE surface. Consequently, a deep study of the band alignment between the photoactive material and the active redox couples has to be carried out to maximize the storage performance, stability and ensure an optimal thermodynamic compatibility in the integrated system [75].

Although there are many articles describing rechargeable batteries, such as Li-ion batteries, Li-ion [111], Li–O_2_ batteries [117, 118], Li–S batteries [109, 119], non-Li anode–air batteries [73, 120] and metal photo-intercalation batteries [113], based on the working electrode composition, which are built on different working principles and also review articles focusing on nanomaterials in non-photo rechargeable batteries, there is no publications concerning photoactive nanomaterials for solar rechargeable batteries. Considering the importance of the PE material in integrated solar-batteries, the review is organized by PE composition, rather than battery configurations or types as shown in previously published review articles.

We focus on nanostructured materials because of the unique characteristics arising from their nanometer size. Compared to bulk materials, lower dimensional (2-, 1-, 0-dimensional: 2D, 1D, 0D) nanomaterials tend to have a more uniform size distribution, which enables the control of their properties. Their higher surface area allows larger contact area with the electrolyte, thus, higher ion flux across the interface [121, 122]. As a result, the rate and the kinetics of ion insertion/removal are enhanced in intercalation batteries. Additionally, they facilitate reactions that cannot occur by materials composed of micrometer-sized particles. For instance, reversible ion intercalation can take place in the mesoporous nanostructure of β-MnO_2_ without any structure destruction [51]. Another advantage is that in some cases, due to the orientation of the nanostructures (*e.g.* nanowire arrays) the charge transfer is favored in one specific direction, reducing charge recombination. It also tends to be easier to tune the bandgap and electronic band structure of nanomaterials by modifying their size, doping, etc. [123]. Nanomaterials usually work throughout photocatalytic reactions that benefit from aforementioned high specific surface areas, optimal interfacial charge transfer and high porosity [124, 125]. However, it may be noted that their large surface area could lead to a large number of side reactions with the electrolyte and the synthesis of a particular nanostructure could be relatively complex to achieve [60]. In the following section, a description of state-of-the-art nanomaterials employed in solar rechargeable batteries is presented.

### 3.1 Dye-sensitized photoelectrodes

The idea behind dye-sensitized photoelectrodes stems from DSSCs, where an organic dye is commonly attached to a layer of mesoporous TiO_2_ (or an alternative wide bandgap oxide) with a ∼20 μm particle size. The film is commonly deposited by doctor blading or screen-printing techniques and its thickness is usually 5–20 μm [24, 126]. When light strikes the dye, the photogenerated electrons are transferred to the CB of TiO_2_ and the holes are quenched by electrons coming from the electrolyte. Frequently, this is an organic solvent containing a redox system such as iodide/triiodide (I^−^/I_3_^−^). Desirable morphologies of TiO_2_ films have mesoporous channels or nanorod nanostructure aligned in parallel to each other, but perpendicular to the contact or charge collector [127, 128]. A thin dense blocking layer of TiO_2_ is also often deposited between the mesoporous layer and the charge collector to prevent direct contact between the later and the electrolyte [24, 126, 129, 130]. The advantages of this morphology include a high surface area for sensitizer adsorption, favorable electron transport and enhanced light-scattering by metal oxide [127]. All these together contribute to increased light absorption of (and thereby charge generation in) the dye sensitizer and charge carrier separation. Because the dye is mainly responsible for light absorption in this system, it is desirable to have one, whose absorption covers a wide wavelength range in the visible regime and even extends to the near-infrared (NIR) part. Similarly important, considering the device lifetime, the dye should be electrochemically and thermally stable and also have a strong binding with the metal oxide. In the case of the strongest interfacial interaction, the dye is covalently bound to the semiconductor surface. It must thus contain anchoring groups (*i.e.* –COOH, –SO_3_H). The energy levels should allow for electron transfer to TiO_2_ and regeneration by the electrolyte [126, 130]. The photoelectric conversion remains as the key factor that determines the overall efficiency of the device [30]. Several publications have used this DSSCs approach [90, 105, 131].

**Figure 3:**
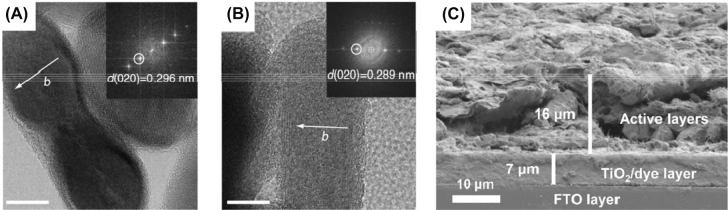
Structure and morphology of dye-sensitized photoelectrodes. (A) HRTEM of pristine LFP (scale bars, 10 nm) and (B) HRTEM of LFP after light exposure (scale bars, 10 nm) [60] Copyright 2017, Springer Nature. (C) High magnification cross-sectional view SEM images of the hybrid N719/S cathode [113] Copyright 2022, Elsevier.

One example is the report of McCulloch et al. where the PE is formed by TiO_2_ sensitized with ruthenium-based Z907 dye in an SFB [132]. They achieved Coulombic efficiencies up to 91% over 50 cycles along with a high range of pH. In 2017, Paolella et al. published triphylite (LiFePO_4_, LFP) nanoparticles (NPs) sensitized with N719 dye in Li-ion batteries [83]. LFP showed complete delithiation by the photo-holes produced in the dye when illuminated, leading to the formation of heterosite (FePO_4_). High-resolution transmission electron spectroscopy (HRTEM) found that the lattice constant of LFP decreased from 0.296 nm to 0.289, confirming the reduced volume of the de-lithiated FePO_4_ structure (see Figure 3A and B). The photo-oxidation and overall efficiency were promoted by ball-milling LFP, which greatly increased the surface area, facilitating the charge transfer between the dye and LFP nanoplatelets. Despite a relatively low photon-to-electric efficiency of 0.06–0.08%, this pioneering work revealed that photogenerated charges can be stored chemically as a solid electrolyte interphase (SEI) layer at the Li metal electrode (as it happens in a classic Li-ion battery, where Li^+^ ions are deduced to Li metal) but without the help of an external circuit [37]. The low current efficiency was attributed to large charge recombination losses at the LFP/dye/electrolyte interface. Since the dye cannot store the photogenerated charge, Xu et al. designed a composite formed by TiO_2_/N719 dye/Cu_2_S to act as photocathode in a solar rechargeable Li–S battery [133]. By depositing Cu_2_S on top of the dye, the third electrode is no longer needed, creating a bifunctional PE that can harvest light and store it. In the first discharge step, Cu_2_S is reduced to Cu(0) and Li_2_S is formed. During photocharging, the dye has the same function as in a regular PBAT, but instead of being regenerated by the electrolyte, the photogenerated holes oxidize Cu/Li_2_S into Cu_1.96_S/Li^+^ while electrons reduce Li^+^ to Li metal at the anode. The following discharge steps consist of the reduction of Cu_1.96_S/Li^+^ into Cu/Li_2_S. The oxidation reaction between the dye^+^ and Cu_2_S is expected to be more efficient and stable compared to that of dye^+^ and the common electrolyte used in DSSCs. The reason relies on the shorter charge transfer path at the dye/Cu_2_S interface, and the avoidance of degradation/incompatibilities between the electrode and electrolyte [134]. In 2021, Li et al. also reported a charging voltage decrease of 0.12 V in a photo-assisted Li–S battery [135]. In this case, N719 dye was covered by a 16 μm of a S layer (see SEM image in Figure 3B) that acted as the energy storing component. Its surface area and conductive properties were enhanced by ball-milling sulfur, carbon nanotubes and binder all together. This is favored also because of the increased roughness and porosity of the sulfur layer that can be observed in the SEM images. However, the capacity faded along charge–discharge cycles due to the dissolution of polysulfide mediator product.

### 3.2 Transition metal oxide-based semiconductors

#### 3.2.1 Titanium oxide

Titanium dioxide (TiO_2_) is a well-known *n*-type semiconductor due to its interesting properties: high stability, nontoxicity, biocompatibility, light absorption, and high electron mobility [136, 137]. It exists in three forms: anatase, rutile, and brookite. Rutile is the most used structure because it is chemically more stable, and scatters white light more efficiently compared to anatase. However, anatase shows enhanced surface chemistry and a higher conduction band-edge, which makes it more suitable for photocatalytic applications [138, 139]. One way to increase the surface area and the diffusion coefficient and decrease charge recombination is through synthesis of nanostructures (nanotubes [140, 141], nanotube arrays [142], nanobelts [143, 144], nanowires [145], nanowire arrays [146], mesoporous films [147, 148], etc.). TiO_2_ nanostructures provide more active sites and facilitate reactions or interactions between the electrode and the active species. These interactions occur mainly on the surface and are therefore very dependent on nanostructure morphology [149], [150], [151]. Their properties and photocatalytic activity will vary depending on the particle size, shape, degree of crystallinity and ratio of anatase to rutile [150]. The main drawback is limited absorption in the visible range, which hinders the generation of photocurrent under solar illumination. Because of its remarkable properties, TiO_2_ has found numerous applications, including photocatalytic degradation of pollutants [152], hydrogen generation [153], photovoltaic devices (solar cells [154], batteries [155] and capacitors [156]), air purification [157] and antimicrobial uses [158]. It has also been widely used as electrode material in rechargeable batteries [159], [160], [161], [162] and, more recently; it was also employed in PBATs.

**Figure 4:**
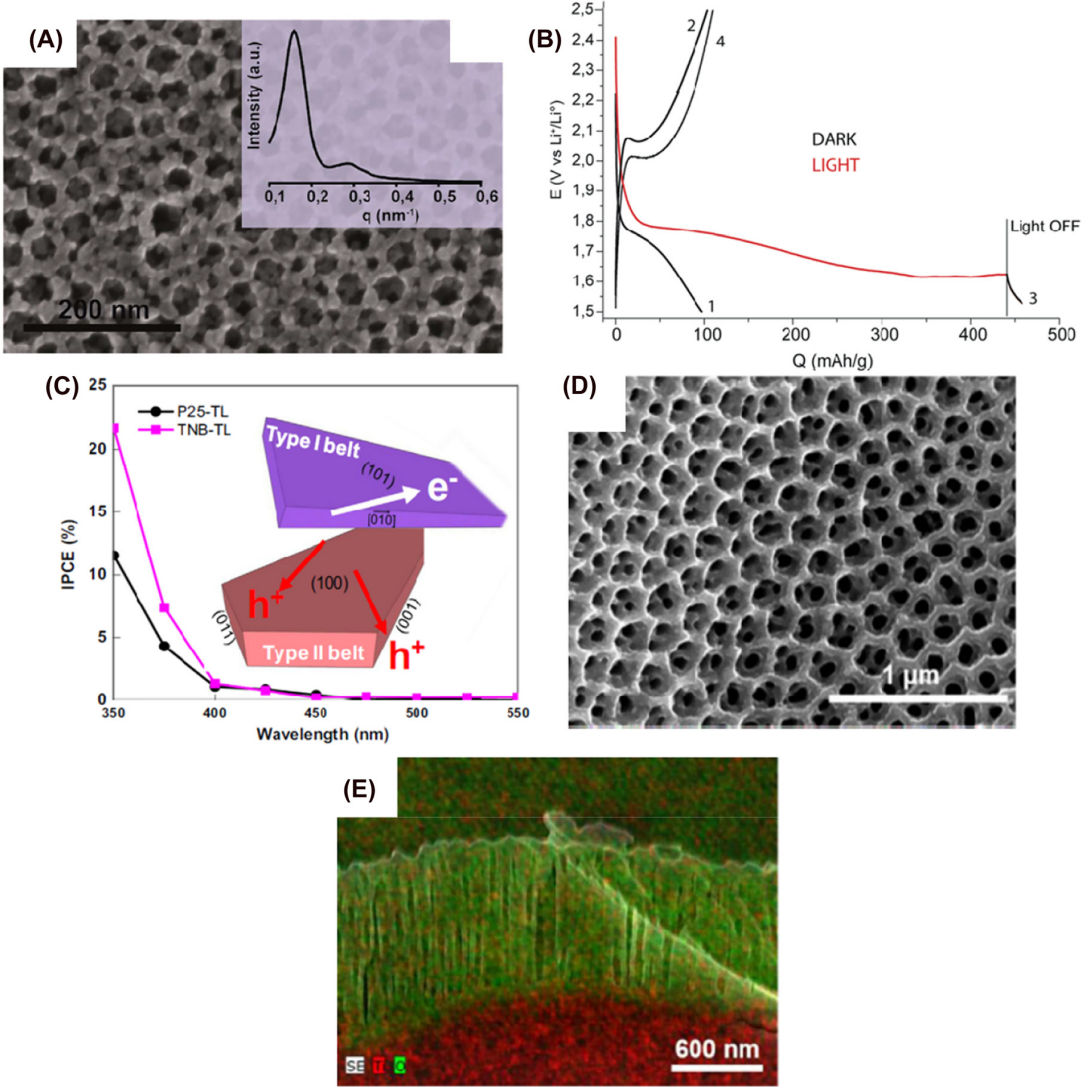
Structure and performance of titanium oxide-based photoelectrodes. (A) SEM-FEG image of the top of the mesoporous TiO_2_ film. The inset corresponds to grazing-incidence small-angle X-ray scattering scan of the film. (B) Discharge-charge curves under dark (black) and light (red) conditions. Discharge no. 3 was performed under light [141]. Copyright 2017, Royal Society of Chemistry. (C) IPCE spectra of all-vanadium photoelectrochemical device assembled using titanium TiO_2_ nanobelts after TiCl_4_ treatment, as the photoelectrode [146]. Copyright 2016, Elsevier. (D) SEM image of the surface morphology and (E) cross section-EDX of anatase-TiO_2_ nanobelts photoanode [152]. Copyright 2020, Elsevier.

In 2017, Nguyen et al. designed a photocathode based on a mesoporous anatase-TiO_2_ thin film of 280 nm in thickness [163], for an integrated Li-ion photobattery. In the SEM-FEG (Scanning Electron Microscopy-Field Emission Gun) top image (Figure 4A) a 3-dimensional (3D) interconnected network with pores of an average diameter of 17 nm with a high degree of pore organization was observed. However, the pore diameter decreases with the depth of the layer until a minimal diameter of 12 nm is achieved next to the interface with the fluorine-doped tin oxide (FTO) substrate. This morphology helps with charge separation and transport of both electrons and ions, which results in enlarged capacity in comparison with a non-mesoporous nanostructure (240 mA h g^−1^) when illuminated and in the dark (180 mA h g^−1^). This is explained by insertion of Li^+^ ions into the TiO_2_ network, forming first a Li-poor phase (tetragonal Li_*α*_TiO_2_, 0 < *α* < 0.25) and Li-rich phase (orthorhombic Li_0.5_TiO_2_) that coexist during charge and discharge processes. On the other hand, the photogenerated holes oxidate Ti^3+^ to Ti^4+^, boosting the lithium ions extraction at the same discharge rate [164]. When the battery is discharged under illumination, Li^+^ has been extracted and inserted simultaneously, which would allow for a theoretically-infinite capacity (see Figure 4B and C) without any signs of electrode deterioration. These results are in agreement with previous reports [165]. Furthermore, the authors suggest that surface plasmon resonance effect of lithiated nanocrystals might have a noticeable impact on the titanium dioxide oxidation process [166, 167], which warrants further studies.

Another strategy to increase the photocurrent and consequently the capacity is the synthesis and application of ultralong TiO_2_ nanobelts (80–110 nm wide, 10 nm thick and 20 μm long) [168] in all-vanadium RFB. The authors describe the effect that stirring has on the nanostructure and morphology of TiO_2_ nanobelts and their advantages versus conventional nanospheres. It was shown that raising the stirring speed caused an increase in the length-to-diameter ratio. As a result, crystallinity, light scattering and absorption are greatly enhanced while charge recombination and the specific surface area decreases [169, 170]. The porosity of the semiconductor layer had also a positive effect in the interaction with the electrolyte, amplifying the photogenerated current. In addition, treatment with TiCl_4_ shifted the absorption of TiO_2_ to longer wavelengths, improving photogenerated charges even more, showing 22% incident photon-to-current efficiency (IPCE) without any external bias. Additionally, the TiCl_4_ treatment also improved electron diffusion and charge separation, reduced charge recombination and generated more exposed high-energy facets acting as reactive sites for the oxidation of active redox vanadium-based species in the electrolyte (see Type II belt, Figure 4D). This is highly beneficial for RFBs where the working mechanism fully relies on redox reactions between the electrodes and the liquid electrolytes.

In contrast with the previous example, Gong et al. reported TiO_2_ nanorod arrays as the photoactive material in the PE for Li–O_2_ batteries that are shorter and thicker (200 nm in diameter and ∼1 μm long). In this case, the challenge consists in boosting the performance of the OER so the formation of Li_2_O_2_ is reversible during the charging process under illumination. Rutile TiO_2_ structures with the nanorod morphology grant high oxygen diffusion and complete infiltration of the electrolyte. They also provide ample space to deposit Li_2_O_2_ as “storing product”. The charging and discharging voltage are 2.86 and 2.65 V respectively for the first cycle, increasing to 3.03 V (charging voltage) after 30 cycles when illuminating, which is close to the theoretical voltage (2.73 V) calculated from the conduction band of TiO_2_ and oxidation potential of Li/Li^+^. However, the discharge voltage increases to 2.85 V after 30 cycles. This unexpected result could be explained by an improvement on the oxygen reduction reaction (ORR) performance related with the generation of more oxygen defects which produce intrinsic changes in the rutile TiO_2_ structure (*i.e.* formation of Ti^3+^). These defects are a consequence of the photogenerated electrons during the charging step that would reduce TiO_2_. It is well known that defective metallic oxides provide much higher ORR and OER performance than their high crystalline counterparts [171], [172], [173]. In 2020, Han et al. proposed a photo-rechargeable seawater battery with two compartments utilizing sodium metal electrode as anode and TiO_2_ nanotube arrays as photocathode [174]. Pure anatase phase annealed at 500 °C exhibited the largest photocurrent compared to mixed anatase-rutile and pure rutile because of its higher crystallinity. In Figure 4E and F, the hierarchically nano-ring structure on the top and the vertically aligned nanotubes on the bottom of the TiO_2_ layer can be observed from the SEM top-view and cross-section images. The nanotubes have an average diameter of 100 nm, a wall thickness of ∼20 nm and a length of ∼800 nm. The 1D nanostructure promotes high electron mobility and the high surface area at the outer part enhances light absorption and contact with the electrolyte. The authors observed that the morphology, crystallinity, and crystal phase of TiO_2_ nanotubes are dependent on the annealing temperature. Formation of clusters and morphological disorders and decrease in porosity were noticed when annealed at temperatures higher than 750 °C [175]. It is important to note that, the morphology, crystallinity and crystal phase of nanomaterials are generally dependent on the annealing temperature. In the two-electrode system based on this PE, the charge and discharge voltages were 2.65 and 2.50 V, respectively.

Although TiO_2_ has proved to be a promising candidate for photobatteries, its poor response in the visible-light spectra originating from its large bandgap and the high charge recombination rate hinder its performance in photoelectrochemical devices. Designing heterostructure composites by combining TiO_2_ with other semiconductors, dyes or plasmonic materials can effectively overcome the abovementioned limitations and boost the redox reaction kinetics. Many examples have been reported over the last years in fields such as photocatalytic degradation of antibiotics [176], solar cells [177], and solar fuel generation [178]. Some publications demonstrated the successful design of TiO_2_-based heterojunctions as the photoactive material in photo-rechargeable batteries. For instance, TiN/TiO_2_ composite nanowires [179] and TiO_2_–Fe_3_O_2_ heterojunction [180] positively reduced the overpotential to 0.19 V with a round-trip efficiency of 94% for the first composite and 86% after 100 cycles for the later. Tong et al. proposed TiO_2_ nanotube arrays with gold nanoparticles in a photo-assisted Li–O_2_ battery with 100% Coulombic efficiency mostly due to photocatalytic processes that effectively reduced the overpotential [181]. Among all the TiO_2_-based nanomaterials, TiO_2_ nanorods and nanowires so far exhibited the highest round-trip energy efficiencies due to the higher surface area compared to other nanostructures (NPs or mesoporous network), exposing a greater number of active sites, and enhancing light harvesting.

#### 3.2.2 Hematite (Fe_2_O_3_)

α-Fe_2_O_3_ is one of the most Earth-abundant metal oxides and is thermodynamically more stable than other iron oxides in the presence of oxygen. It is chemically stable over a wide pH range, of low-cost and sustainable, and has a relatively narrow band gap of 2.1–2.3 eV, which is beneficial for light absorption in the visible range of the spectrum. It is considered an *n*-type semiconductor with magnetic properties [182, 183]. It has been used in many fields, such as photocatalysis, water-splitting, CO_2_ removal, energy storage systems, and gas sensing [184], [185], [186]. The known nanostructures of α-Fe_2_O_3_ are nanorods [186], nanorods arrays [187, 188], nanoflowers [189], microcubes [190], nanowires [191], nanotubes [185, 188], nanoflakes [192], and nanoparticles [193] among others.

**Figure 5:**
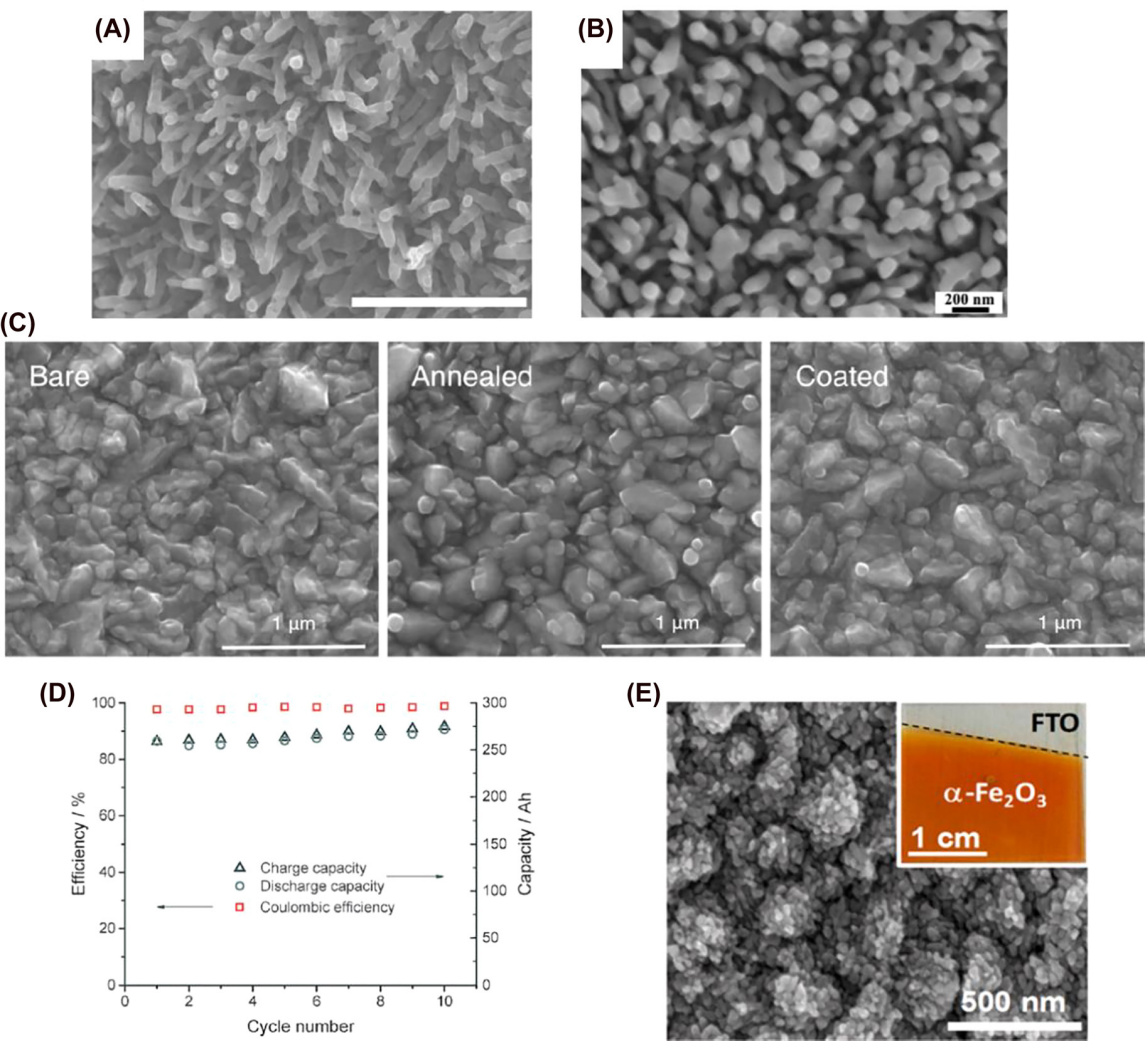
Structure and performance of hematite-based photoelectrodes. (A) SEM image of α-Fe_2_O_3_ film (scale bar 1 μm) [172]. Copyright 2019, Springer Nature. (B) SEM image of α-Fe_2_O_3_ film [173]. Copyright 2020, Elsevier. (C) Field emission scanning electron microscope (FE-SEM) images of bare, annealed and coated α-Fe_2_O_3_. (D) Capacity data for 10 cycles of charge–discharge cycles with a constant current density of 20 mA cm^−2^ [175]. Copyright 2016, John Wiley and Sons. (E) Top-view SEM and digital (inset) images of hematite granular NPs [176]. Copyright 2016, American Chemical Society.

Liu et al. and Gong et al. presented α-Fe_2_O_3_ nanorods as PE in Zn–air and Li–O_2_ batteries respectively [85, 86]. They performed a hydrothermal synthesis followed by two-and one-step annealing processes, respectively. The annealing at 800 °C ensures the doping of Sn into the Fe_2_O_3_ nanostructure to increase its conductivity. However, nanorods that went through two annealing processes (550 °C for 2 h and 800 °C for 10 min) have smaller diameters and look highly ordered, suggesting that this intermediate step is beneficial to control the size and shape of α-Fe_2_O_3_ nanorods. They show an average diameter of 70 and 80 nm, and film thickness of ∼350 and 450 nm, respectively (Figure 5A and B). Plenty of space was observed between nanorods, which is considered essential for electrochemical reactions on the PE surface. This is specifically relevant for the photogenerated holes, which have a strong ability to oxidize water into oxygen. This structural feature thus facilitates OERs which have sluggish kinetics in conventional rechargeable batteries. The photogenerated electrons in the CB of the semiconductor are transferred to the metal electrode for reduction of the metal ion (M^+^ + e^−^ → M(0)). The highly stable surface morphology does not change with charge and discharge cycles in both cases, which was confirmed by X-ray diffraction (XRD) and X-ray photoelectron spectroscopy (XPS). As a result, it delivers a stable current density over time. α-Fe_2_O_3_ PE shows absorption in the visible range with an absorption edge of ∼600 nm and an optical bandgap of ∼2.10 eV. Both works show an improvement in the charging voltage, thus, in the round-trip energy efficiency up to 70.3 and 87.7%, respectively. α-Fe_2_O_3_ nanorods PE in the Zn–air photobattery yielded charge and discharge voltages of 1.64 and 1.15 V for several cycles over 50 h under illumination [86]. Gao et al. reported a reduction of the charging voltage, decreasing from 3.96 to 3.15 V under illumination, with a discharge voltage of 2.56 V, resulting in an energy efficiency increase of 16.6% for the Li–O_2_ system [85]. Due to a reduction in resistance of the charge transfer along the electrode and its interface with the electrolyte, α-Fe_2_O_3_ nanorods show enhanced photoelectrochemical response when they are highly ordered [194]. The same configurations were tested using BiVO_4_ nanoplates with no positive results due to their dissolution in alkaline environments and aggressive photocorrosion.

In 2016, Wedege et al. utilized hematite synthesized by spray pyrolysis as the photoanode for aqueous alkaline SFB. They used ferrocyanide and anthraquinone-2,7-disulphonate (AQDS) in a solution of NaOH as electrolytes in the anode and cathode side respectively divided by a conductive cation membrane [195]. Since hematite suffers from back electron recombination [196, 197], the photogenerated electrons reduce ferricyanide instead of AQDS. Therefore, the experimental charging voltage under illumination is lower than expected taking into consideration its energy levels (CB and VB) under alkaline conditions. Polyaniline (conjugated polymer formed by blocks of benzyl rings linked by an amine) was deposited on the surface of hematite to overcome this issue and compare the results with bare and annealing-only samples. It was proven that low-temperature steam annealing significantly reduces surface defects [198], creating a smoother hematite layer that facilitates the deposition of a more homogeneous layer of polyaniline. FE-SEM (see Figure 5C) was performed to evaluate the morphology of the samples showing granular particles of different sizes ranging from 0.1 to 0.5 μm for annealed and coated samples and more irregular particles for bare hematite. The polyaniline layer must be thin to allow light absorption by the hematite. This coating enhances the photoelectrical response because its lowest unoccupied molecular orbital (LUMO) level is lower than the CB of the α-Fe_2_O_3_. This prevents the previously mentioned back electron recombination to the ferrocyanide electrolyte while allowing the holes from the VB to move to its HOMO level. Although the solar-to-chemical-energy conversion efficiency for this SFB is still rather low (0.08% for the coated hematite), this work shows the possibility of a pure solar rechargeable redox flow battery. The same year, Nikiforidis et al. published a Li–I_2_ SFB with granular nanoparticles of hematite photocathode with a film thickness of 90 nm. In this case, the PE was synthetized by anodic electrodeposition followed by annealing at 550 °C [199]. The charging voltage under illumination was reduced by 0.66 V, with an increase in the energy efficiency of ∼21% when compared with the same device under dark conditions. However, unlike the previous example, no full charge was observed in the absence of an external bias; charging fully under illumination could only reach up to 10% state-of-charge (SOC). The SEM image in Figure 5E depicts the PE surface after electrodeposition of the hematite and annealing, which shows many particles with an average diameter of 33 nm. The surface appears to be less smooth with less defined nanoparticle shape compared to the PE described by Wedege et al. The reduced surface area of the granular nanoparticles compared to nanotubes or nanowires [200] in contact with the electrolyte decreased photocatalytic activity which resulted in lower energy efficiency and the impossibility of unbiased charge. The low temperature steam annealing and the deposition of polyaniline had a positive effect on the PBAT performance.

PEs that employed α-Fe_2_O_3_ nanorods obtained higher specific capacity values and more stable devices in comparison to α-Fe_2_O_3_ NPs, but lower overall device efficiencies. However, it should be noted that the described systems are quite different (Zn–O_2_, Li–O_2_, Li–I_2,_ and RFB) and they do not show standardized experimental parameters to allow accurately comparing the performance of these PBATs and indisputably assessing the effect of morphology.

#### 3.2.3 Tungsten trioxide

The transition metal oxide tungsten trioxide (WO_3_) is an *n*-type semiconductor with a bandgap of ∼2.6–3.0 eV [201]. It responds to visible light, making it an optimal PE candidate. WO_3_ nanoparticles have high specific surface area, impressive hole diffusion length, and high electron mobility (due to good surface permeability), and are nontoxic, as well as being chemically and photochemically stable [202]. WO_3_ is often present as sub-stoichiometric oxide (WO_3−*x*_) due to the presence of numerous oxygen vacancies, which affects the electron density and its conductivity [203], [204], [205]. Monoclinic is the most stable phase of WO_3_ at room temperature and has the greatest absorption in the visible range of the spectrum among all of its phases, making it more desirable for photocatalytic applications [206, 207]. Among the most common WO_3_ nanostructures, we can find nanorods, nanosheets, 3D nanostructured *papilio paris* and thin films being used by themselves or as part of heterojunctions with other materials [208], [209], [210], [211], [212]. They have been widely used as photodetectors [213, 214], as photocatalysts [215, 216], for water splitting [212, 217] and in gas sensors [218, 219]. On top of these uses, there have been several publications where WO_3_ was used as a PE in PBATs.

**Figure 6:**
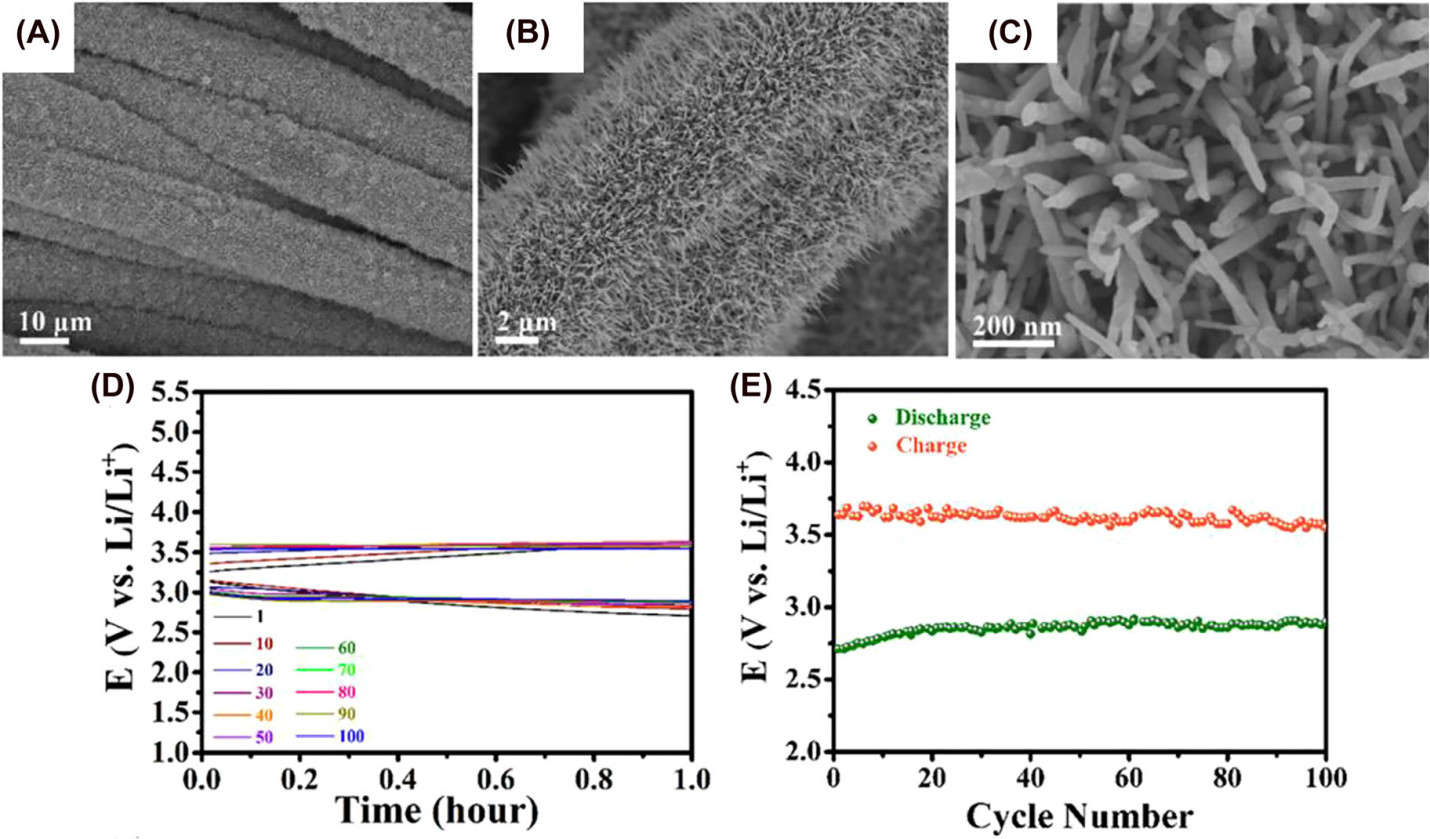
Structure and performance of tungsten trioxide-based photoeletrodes. (A–C) SEM images of WO_3_-AN NWs. (D and E) Charging and discharging curves of Li–O_2_ PBAT using WO_3_-AN photocathode at 0.06 mA cm^−2^ [200]. Copyright 2019, American Chemical Society.

In 2019, Feng et al. reported WO_3_ nanowire (NWs) arrays grown *in situ* on carbon textiles in Li–O_2_ batteries [220]. By controlling the reaction time of the hydrothermal synthesis, they achieved an optimal coverage of the tungsten oxide precursor on the surface of carbon fibers at 16 h. A heating treatment was subsequently needed to control the crystalline phase of WO_3_ [201]. Generally, the greater the degree of crystallization, the less active sites and surface area WO_3_ has negatively affects photochemical reactions [221]. Three samples were prepared at different temperatures under air and/or nitrogen (see Table 1). The samples prepared under nitrogen atmosphere have more defects, which is detrimental for hole diffusion and electron mobility. However, all of them displayed absorption in the range of 500–800 nm due to oxygen vacancies [222]. WO_3_-AN is the best PE candidate since it has a smaller bandgap (2.63 eV), which leads to more visible light absorption, charge separation and fewer defects as confirmed by XPS measurements. Monoclinic WO_3_-AN absorbs more visible light than the hexagonal structure and also has fewer defects, which grants more beneficial hole diffusion length and electron mobility. Consequently, we can expect a higher rate. SEM image (Figure 6–C) shows WO_3_ NWs grown vertically, covering the surface of the carbon fiber uniformly, and having a high surface area (high number of active sites, very beneficial for the photocatalytic ORR). It shows the lowest charging potential (∼3.55 eV) under illumination, maintained over 100 cycles, demonstrating high stability under light irradiation. This was attributed to its monoclinic phase and relatively fewer defects.

**Table 1:** WO_3_ synthesis conditions and structure [200].

Name	Heating temperature (°C)	Time (hours)	Conditions	Phase
WO_3_-A	450	2	Air	Hexagonal
WO_3_-N	550	2	Nitrogen	Monoclinic
WO_3_-AN	(1) 450	2	Air	Monoclinic
	(2) 550	2	Nitrogen	

In 2020, the same group reported a photocathode made up of WO_3_ NW arrays decorated with g-C_3_N_4_ for Li–O_2_ batteries [223]. The morphology of WO_3_ NWs arrays is highly comparable to their previous report. The advantage of this heterojunction is facilitating the separation of the electron–hole pair and inducing faster migration of charges. Thus, charge recombination is avoided. It also increases visible absorption, which enhances catalytic performance. Furthermore, g-C_3_N_4_ acts as a co-catalyst, providing new active sites for the ORR and lowering the overpotential 3.69 V (*vs* 3.99 V from WO_3_ NW array).

#### 3.2.4 Vanadium oxides

Vanadium is considered a highly abundant element in the Earth’s crust [224]. It shows multiple oxidation states and presents an elevated number of crystalline structures with different oxygen coordination [225, 226]. The most common crystalline structures are VO_2_, V_2_O_5,_ and V_6_O_13_. The two first ones have already been used as cathode materials in Li-ion batteries [227], [228], [229], showing high specific capacity and energy density as well as good photocatalytic properties, which also make them promising materials for use in photobatteries. De Volder’s group has published several articles during recent years using vanadium oxides as PEs in PBATs.

**Figure 7:**
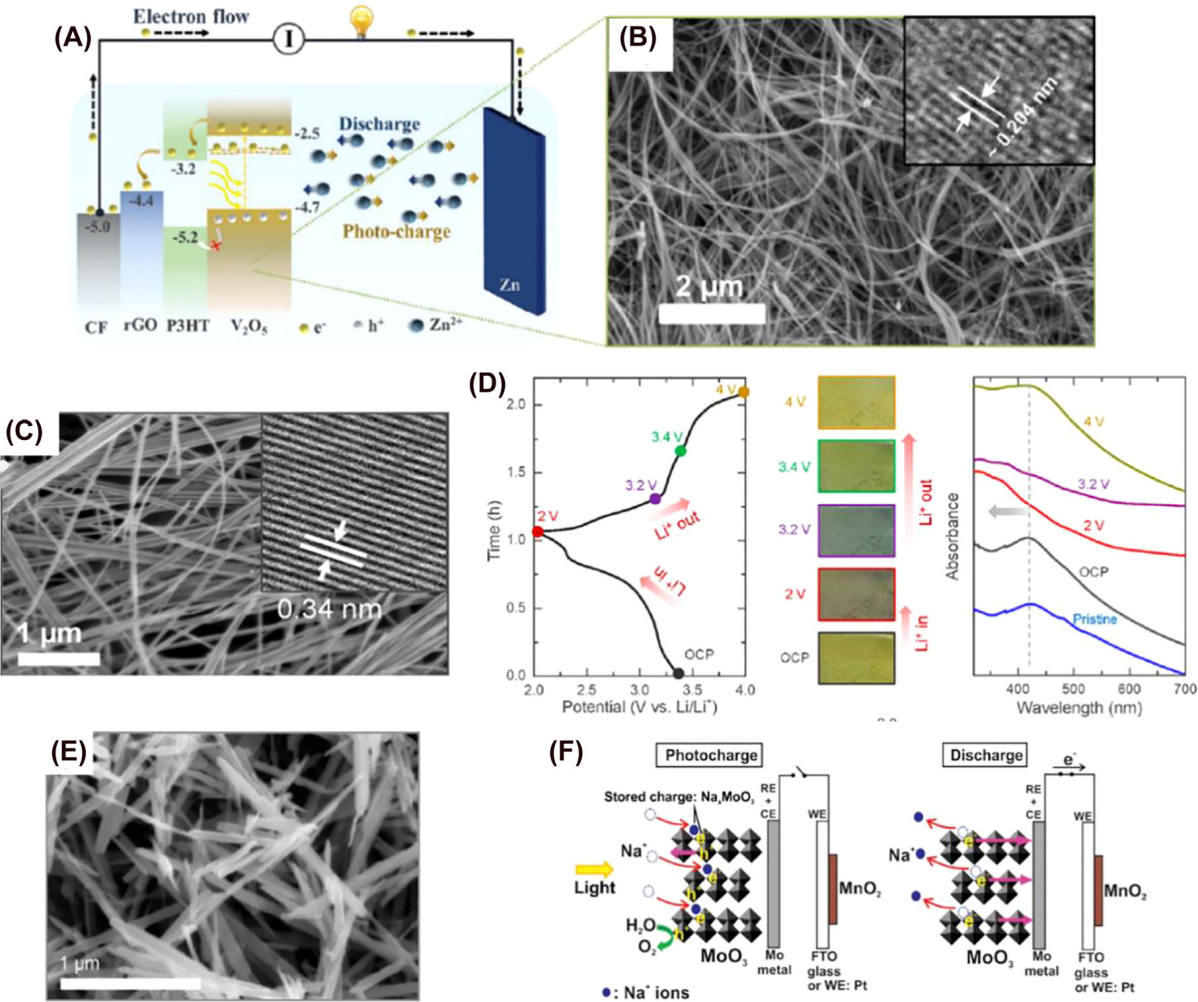
Structure and performance of vanadium oxides-based photoelectrodes. (A) Photocharging mechanism of Zn–air PBAT. (B) SEM images of V_2_O_5_ nanofibers and inset HR-TEM image of Zn–air PBAT [69]. Copyright 2020, Royal Society of Chemistry. (C) SEM image of V_2_O_5_ nanofibers and inset HR-TEM image of Li-ion PBAT. (D) Initial discharge–charge curve using an optical-cell, images showing the change in color as a function of the SOC, and *ex situ* absorbance spectra of the PE at different SOC of V_2_O_5_ nanofibers in Li-ion PBAT [211]. Copyright 2021, American Society of Chemistry. (E) SEM image of VO_2_ nanorods [215]. Copyright 2021, Royal Society of Chemistry. (F) Photocharging and discharging mechanisms of Na-ion PBAT [216]. Copyright 2017, John Wiley and Sons.

In 2020, his group reported a photo-to-electric conversion efficiency of 1.2% with a system containing V_2_O_5_ nanofibers mixed with poly(3-hexylthiophene-2,5-dyil) (P3HT) and reduced graphene oxide (rGO) as PE, in Zn-ion battery [59]. The photogenerated electrons travel to the Zn electrode while holes are blocked by P3HT and accumulate in the photocathode (Figure 7A). They observed a clear increase in capacity when the PE is exposed to light. Furthermore, the capacity of the battery increases under illumination even during the discharge process. V_2_O_5_ nanofibers show orthorhombic structure with diameters of 50–100 nm with an interplanar spacing of ∼0.204 nm (see SEM and TEM images in Figure 7B). The optical bandgap of 2.2 eV ensures light absorption in the visible range. The advantages of using V_2_O_5_ nanofibers versus other nanostructures include higher conductivity along the nanofiber length and larger surface area, which will provide more active positions for interactions with other molecules or ions [114]. Combination with rGO provides an optimal lithium-ion diffusion and electron transfer to the cathode and high cycling stability. It also impedes the agglomeration of nanostructures [230].

In 2021, Boruah et al. of the same group reported the same photocathode structure in Li-ion batteries (rGO, P3HT, and V_2_O_5_ nanofibers) [66]. The difference was in the nanofibers’ diameter, which was found to be between 20 and 50 nm (see Figure 7C), considerably smaller compared to their previous work, and an increase in the interplanar spacing up to 0.34 nm. During charge, the oxidation state of vanadium changes due to the accumulation of the photogenerated holes, releasing the Li^+^ that was intercalated in the V_2_O_5_ nanofibers, Li^+^ travels to the anode, where the electrons are been collected, and it is reduced to Li metal. V_2_O_5_ nanofibers experience reversible phase transformations when they are oxidized [231, 232], something that takes place during the charging and discharging steps. These phase transformations can be observed because of changes in the band gap energy. This is visible by looking at the color change of the photocathode in the charge and discharge states and it is due to lithium insertion/removal [233], as it is shown in Figure 7D. The achieved photo-to-electric conversion efficiency with this system was 2.6% under 455 nm illumination and 0.22% for 1 sun illumination without the need for an external energy source. The photocharging voltage was 2.82 V, achieving a nearly constant 2.45 V when discharging under illumination. It may be noted that a direct comparison between two different metal-ion batteries should be avoided because of the potential difference of the metal electrodes. However, we can say that nanorod and nanofiber vanadium oxide-based PEs confer higher photo-to-electric conversion efficiencies and allow the device to operate without the need of applying an external bias.

De Volder’s research group published two more articles using vanadium oxides with Zn-ion batteries. The first one replicates the previous report except for removing P3HT trying to reduce the overall cost of the device [234]. In this case, rGO was mixed with VO_2_ instead of V_2_O_5_. They achieved double capacity, higher rate capability, and better capacity retention over 1000 cycles (of about 90%). VO_2_ nanorods have a monoclinic structure and an interplanar spacing of ∼0.35 nm [235, 236]. The band gap of VO_2_ nanorods was obtained from UV–vis absorption measurements, showing an indirect and direct band gap of 2.3 and 2.53 eV. They were higher than that of V_2_O_5_ nanofibers, and thus exhibited a blue-shift in the absorption spectra. They observed greater intercalation of Zn^2+^ in V_2_O_5_ nanofibers during the discharge process due to the photogenerated charges that improve the capacitive contribution of the photocathode, leading to an increase in the Zn^2+^ diffusion kinetics. This is due to the structure of the framework with relatively large tunnels of 0.82 nm approximately along the *b*-axis and 0.5 nm along *c*-axis [237] (Figure 7E). In this case, the insertion of Zn ions does not change the optical band gap. However, the photo-to-electric conversion efficiency achieved was only 0.18%. Afterward, they published another paper on the Zn-ion battery [234] where they showed an increase in the photoconversion efficiency up to 0.51% (at 455 nm illumination). In this example, VO_2_ was deposited on top of ZnO, which acted as hole collector instead of rGO. The improvement in efficiency was due to a more efficient charge separation with ZnO, mainly because of the direct deposition instead of physical mixtures used in the previous publications. The battery capacity in this last work is two times higher than those using V_2_O_5,_ but its efficiency was lower.

The combination of vanadium oxides and carbonaceous materials, such as graphene, carbon nanotubes (CNTs), and carbon fibers, was extensively studied. A thin layer of carbon coating on vanadium oxides was found to have improved cycling stability and electrochemical properties for LIBs [181]. However, the thickness of carbon coatings needs to be carefully controlled, since thicker coatings might impede the diffusion of Li ions [182]. In this regard, mesoporous carbon layers are highly desired to enhance the ion transfer between vanadium oxides and electrolytes [183]. The stacked structures of vanadium oxide and rGO were found to exhibit enhanced capacity and cycling stability [184]. Also, the fabrication of nanocomposites promotes electrolyte diffusion and ion transfer, improving electrochemical kinetics [238]. Structural stability can also be enhanced by compounding multiple strategies such as surface coating and particle-supporting.

#### 3.2.5 Molybdenum oxide

Like vanadium oxides, molybdenum oxides also have light absorption in the visible range of the spectra, several crystal structures, and can incorporate alkali ions in their structures [239, 240]. MoO_3_ has attracted great attention in the last decades due to its nontoxic nature and remarkable performance in fields such as photovoltaics [241], energy storage [242], gas sensing [243], and catalysis [244]. It is an *n*-type semiconductor whose oxygen composition varies as follows: MoO_*x*_, where 2 ≤ *x* ≤ 3. These variations can induce changes in the physicochemical properties (*i.e.* light absorption, electrical conductivity, and localized surface plasmon resonance) [240, 245], [246], [247]. Lou et al. reported a MoO_3_-based photoanode in Na-ion battery that can be charged without applying an external bias [248]. MoO_3_ nanostructure is formed by particles with platelet morphology and an average particle size of 306 ± 170 nm [249]. Na^+^ ions from the electrolyte neutralize the photogenerated electrons and are stored in the lattice structure of the MoO_3_ while the holes move to the electrode surface to oxidize water to oxygen. The process takes place in two steps as the voltage increases. During charge, cation intercalation distorts the orthorhombic α-MoO_3_ phase first to a sodium bronze phase (Na_0.33_MoO_3_), followed by the formation of Na_*x*_MoO_3_ (0.33 < *x* < 1.1) (see Figure 7F). The intercalation/deintercalation is only partially reversible, confirmed by XDR and XPS analysis where the phase composition of the photoanode was shown to be Na_0.7_MoO_3_ after discharge, resulting in a change in the Mo oxidation state equivalent to Na^+^ insertion [250, 251]. Based on these facts, it was demonstrated that the two mentioned phases coexist after 2 h of discharge. Although the authors report no sign of physical deterioration at fast Na^+^ intercalation rates, MoO_3_ dissolves with time, hindering the stability of the photobattery. More research must be performed in order to address this issue.

#### 3.2.6 Cobalt oxide

Cobalt oxides are inexpensive and same as many metal transition oxides; they can delocalize electrons that previously occupied O 2p orbitals to ease the oxidation of Co^3+^ into Co^4+^ during OER [252, 253]. Their catalytic activity can also be tuned by introducing oxygen vacancies, among other strategies [252]. Because of these beneficial properties, the use of cobalt oxides has been explored in photocatalysis as well as EESs, including secondary Li-ion batteries [254, 255]. Spinel Co_3_O_4_ is a *p*-type semiconductor with a direct and indirect bandgap of 1.4–1.8 and ∼2.2 eV, respectively. Cobalt is present in two oxidation states, where Co^3+^ has an octahedral structure and Co^2+^ is tetrahedrally coordinated with oxygen ions, forming close-packed face centered cubic lattice [256]. When Co_3_O_4_ is exposed to light, it becomes a highly active, releasing super-oxidizing species that reacts with OH^−^ and H_2_O. In 2019, microrod structures of spinel-like Co_3_O_4_ interconnected with other Co_3_O_4_ spherical particles of a size of 25 nm were introduced as a bifunctional electrode in Zn–air PBATs [257]. Its mesoporous structure assists the ORR and OER photocatalytic reactions which involve OH^−^ groups during charge/discharge under illumination. Despite its high stability, the difference between charge and discharge voltage, as well as the difference in specific capacity under dark and light conditions are not significant.

### 3.3 Chalcogenide-based nanomaterials

Metal chalcogenides are well-known materials usually referred to MX_2_, where X represents an element from VI A group (X: S, Se and Te) and M a transition metal. MX_2_ are 2D materials with large interlayer distances [258, 259]. They consist of a hexagonal dense layer of M atoms sandwiched between two layers of X atoms. Because of large interlayer spacing and weak van der Waals interaction between layers, metal ions can intercalate into their structure [260, 261]. Because of this, they have already been used as electrodes in Li- and Na-ion batteries [259, 261]. However, they experience lower volume expansion which can be detrimental. They can undergo multi-electron redox reactions and have a fast ion diffusion rate due to the lability of the M–X bond. The bandgap can vary from 0 to 2 eV depending on elemental composition, the number of layers and the presence of doping agents, which allows for tailoring properties according to the desired application [258].

**Figure 8:**
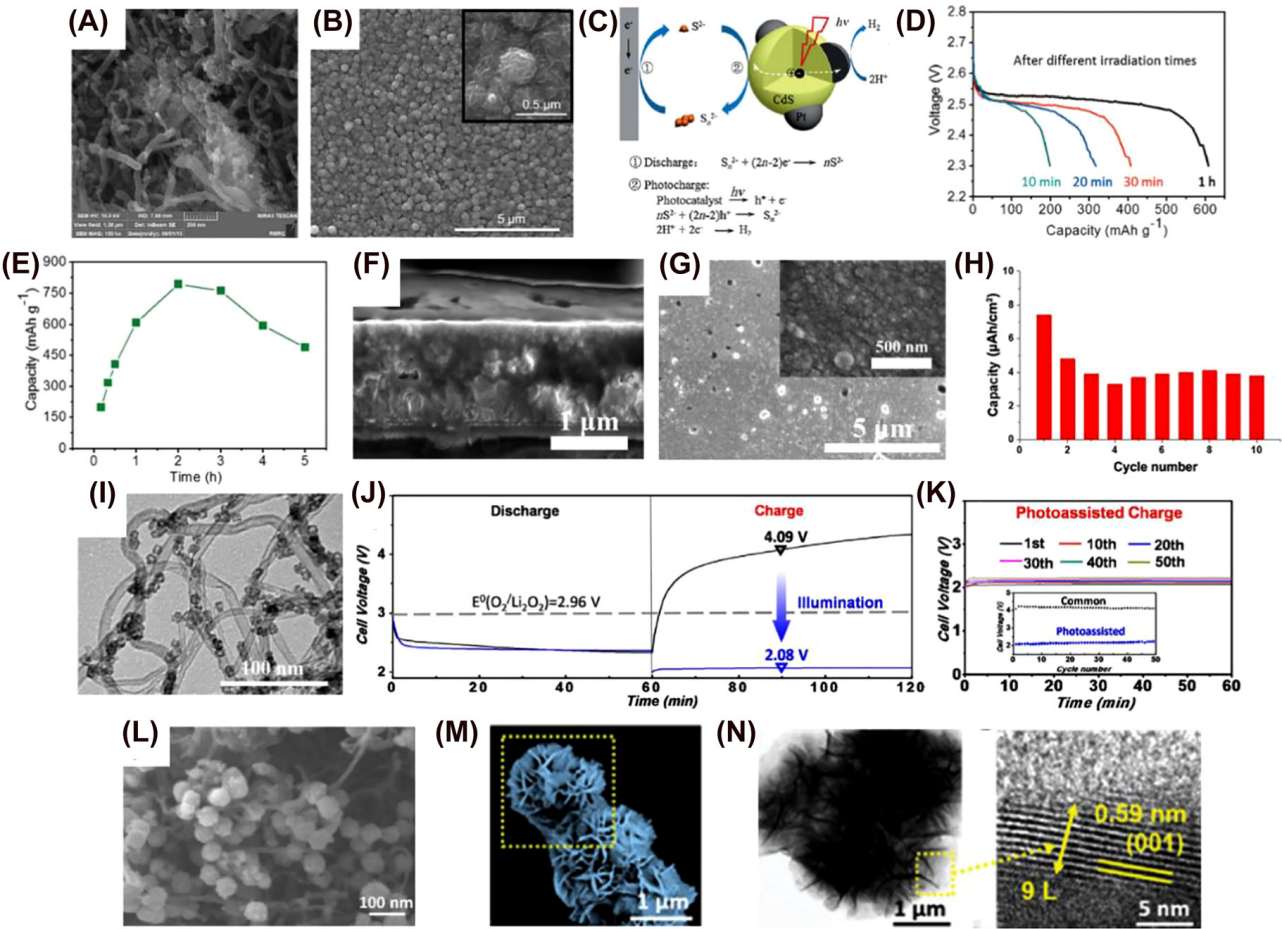
Structure and performance of chalcogenide-based photoelectrodes. (A) SEM image of MWCNT/CdS PE [246]. Copyright 2015, Elsevier. (B) SEM image of CdS photoanode [250]. Copyright 2016, Elsevier. (C) Charging mechanism of Cd/Pt Li–S PBAT. (D) Discharge curves at different irradiation times. (E) Specific discharge capacity versus irradiation time [95]. Copyright 2015, John Wiley and Sons. (F) Cross-section and (G) top-view SEM SE images of CdS-coated WO_3_ film, inset corresponds to high-magnification SEM SE image. (H) Discharge capacity versus cycle number [88]. Copyright 2019, John Wiley and Sons. (I) TEM image of CdSe/ZnS QDs@CNT composite [259]. Copyright 2018, Elsevier. (J) Electrochemical performance of solid-state Li-ion O_2_ battery with (blue line) and without (black line) irradiation at 0.026 mA cm^−2^. (K) Photo-assisted charge curves of photo-assisted solid-state Li-ion O_2_ battery based on the voltage at the half-charge capacity point. (L) SEM image of ZnS@CNT composite [260]. Copyright 2018, Elsevier. (M) SEM and (N) TEM images of SnS_2_ [261]. Copyright 2019, Royal Society of Chemistry.

Cadmium sulfide (CdS) is the most used chalcogenide. It is an *n*-type II–VI semiconductor that has been widely employed as a photocatalyst because of its light absorption capabilities in the visible range. It has high conductivity in addition to its low-cost synthesis. It can be used with other metals or coupled with other semiconductors to prevent electron–hole recombination [262], [263], [264]. Its VB and CV positions are suitable to use with vanadium redox species in SFBs. Two examples are reporting its use as a photoelectrode in this type of PBAT. Peimanifoard et al. reported two and three-electrode SFBs using V^3+^/V^2+^ and VO^2+^/VO_2_^+^ as electrolytes [265]. The photoelectrode is formed by CdS clusters covering the surface of multi-walled carbon nanotubes (MWCNTs), as Figure 8A displays. The clusters are attached to MWCNTs, providing a higher surface area that will facilitate contact with the electrolyte, boosting the photocatalytic reaction. Under illumination, the photogenerated electrons are successfully transferred to the counter electrode and reduced V^3+^ to V^2+^; while the holes oxidize VO_2_^+^ into VO^2+^ in the electrolyte. Nevertheless, it is necessary to apply an external bias. The photo-to-electric conversion efficiency achieves 2.12% when the voltage is 1.38 V (*vs* the CE) (*vs* Ag|AgCl|KCl_3M_) for the two-electrode system. The photoelectrode does not present photocorrosion after 1 h of on–off cycles, a common problem when using CdS. It is brought on by photogenerated holes that fail to be removed in time, causing degradation and dissolution [266], [267], [268]. A similar system was developed by Azevedo et al., which consisted of a two-electrode SFB using V^3+^/V^2+^ and V^3+^/VO^2+^ as active species in the electrolyte and carbon felt as a cathode [269]. The photoactive material was based on CdS nanostructures with 50 nm cauliflower shape (Figure 8B) that were electrodeposited as a thin and compact layer of 200 nm. The changes in the deposition potential gave them different morphologies. These cauliflower-like nanostructures yielded the highest photocurrent performance, owing to their rougher morphology and increase number of active sites for photocatalytic reactions. In order to improve the performance of the battery, a layer of compatible semiconductors was deposited over CdS. TiO_2_ and CdSe were chosen since they have photocatalytic activity in the studied system and have been proven to be stable in the voltage range where the battery functions [270], [271], [272], [273]. Despite TiO_2_ having been widely explored as a protective layer for other semiconductors, including CdS, it did not generate any benefits in this system. The CdS/CdSe PE generated three times more photocurrent (1.4 mA cm^−2^ at 0 V *vs* 0.47 mA cm^−2^) compared to bare CdS. However, none of them was able to provide the sufficient potential to fully charge the SFB without an external bias or enough stability to measure charge/discharge cycles for more than 5 min. More efforts need to be put in this regard by including a sacrificial agent or increasing the pH of the electrolyte [274, 275]. Because of the differences in the counter electrodes and electrolytes, it is not possible to make a straightforward comparison between the above-mentioned SFBs. However, the cauliflower-like CdS nanostructures allowed photocharging of the battery until 90% SOC was achieved. We can conclude then, that this specific morphology allows for better battery performance by increasing the surface area, shortening the diffusion paths, and exposing more active sites, which improves the reaction kinetics with the redox-active species and reduces charge recombination [276, 277].

To improve the stability and performance of CdS, Li et al. proposed the introduction of Pt as a co-catalyst for lithium–sulfur PBATs [119]. The PE is composed of CdS decorated with Pt NPs of 4–8 nm in size. The S^2−^ ions produced during the discharging process are oxidized into polysulfides (S_*n*_^2−^, 2 ≤ *n* ≤ 8) by photogenerated CdS holes during the charging process and the electrons are transferred to the Pt nanoparticles. These electrons will reduce H^+^ from the electrolyte to produce H_2_, a valuable fuel (Figure 8C). Hence, this singular system can store energy electrochemically and generate H_2_ at the same time. Pt NPs can lower the overpotential for water splitting and sulfide-based electrolytes are well known as efficient sacrificial agents that can be oxidized by the photogenerated holes in CdS. This helps avoid recombination between holes and electrons in the semiconductor and prevents corrosion by simplifying the system [263, 278, 279]. In the end, 792 mA h g^−1^ of discharge capacity are obtained at 0.1 mA cm^−2^ rate after 2 h under illumination, being proportional with the irradiation time. The hydrogen evolution rate is 3.04 mmol g^−1^ h^−1^. However, insoluble elemental sulfur (S_8_) is detected after 4 h of irradiation, compromising the battery stability and the cycling performance (Figure 8D and E).

Demopoulos’s and Zaghib’s groups designed a photo-absorbing and energy storing bifunctional photoelectrode based on WO_3_ coated with TiO_2_ and sensitized by CdS for two different types of two-electrode systems: Li and Na-ion intercalation batteries [113, 280]. As explained earlier, WO_3_ allows the intercalation of Li or Na ions in its structure, enabling charge storage under illumination. This is also a well-studied process in Li-ion batteries [220, 223]. Since optical absorption of WO_3_ is limited by its bandgap, a light sensitizer can help improve the photocharging process. WO_3_ (coexisting cubic and hexagonal phases) was formed *in situ* on top of a layer of FTO and treated with TiCl_4_ that leads to the formation of a thin layer of TiO_2_ after heating treatment. TiO_2_ was chosen due to the favorable alignment of its CB with that of WO_3_, facilitating charge transfer and reducing the recombination of holes and electrons [102]. CdS acts as a sensitizer; with a bandgap of 2.5 V versus Li/Li^+^ (−4 eV *vs* vacuum), it has visible light absorption and can efficiently transfer the electrons to WO_3_ during the charging step under illumination. Li or Na ions will compensate for the negative charge due to the accumulated electrons from CdS by intercalation into its crystal structure. The photogenerated holes are collected by sacrificial hole scavengers or polysulfide species (I_3_^−^/I^−^ from LiI in the Li-intercalation battery and, Na_2_SO_3_ and Na_2_S in the Na-ion system). For the Li-intercalation photobattery, SEM images (Figure 8F and G) display the complete infiltration of CdS in the porous structure of WO_3_, forming a low-crystalline structure with a smooth surface; also confirmed by energy-dispersive x-ray spectroscopy (EDS), XPS, and XRD analysis. This morphology seems to be favorable for light absorption and charge transfer to WO_3_, but it hinders the Li ion diffusion. After several photocharging-discharging cycles, the smooth CdS coating cannot be observed. This is the result of degradation by the electrolyte and photocorrosion, which eventually lead to a decay in the photocharging efficiency. However, this degradation is beneficial since it exposes WO_3_ to more Li ions and increases interfacial intercalation. As a result, the stability of the system is compromised but self-discharging over time can be observed (see Figure 8H) [113]. In the Na-ion photobattery, the CdS layer remains stable after several cycles because of the introduction of Na_2_SO_3_ and Na_2_S as hole scavengers. This system achieved 0.3% of overall photo-to-electric energy conversion-storage efficiency [280]. Nevertheless, the scavengers need to be continuously replenished, and the Pt counter electrode is not stable in sulfide electrolytes [281, 282]. For this reason, they replaced Pt with Cu_2_S electrode and added S^−2^/S_*n*_^2−^ as a nonsacrificial redox couple. The resulting device gained stability and the photocurrent was greatly enhanced because of the higher catalytic activity of the new electrolyte [280]. More efforts must be put towards avoiding CdS’s photocorrosion and degradation by the electrolyte, which compromises the battery stability.

In order to decrease the overpotential caused by Li_2_O_2_ in Li–O_2_ photobatteries, Veeramani et al. developed a nanocomposite photocathode containing CdSe/ZnS QD@CNT. Both CdSe and CdSe/ZnS present the same cubic zinc blende structure [283]. The average particle size was 10 ± 0.5 nm with *d*-spacing of 3.42 Å, corresponding to the CdSe planes (111) in the composite. Figure 8I shows the surface of CNTs homogenously covered by the QDs. The absorption spectrum of CdSe QDs demonstrates absorption of photons in the visible range, related with a narrow bandgap like some organic chromophores [284, 285]. The CB of the composite allows efficient oxidation of Li_2_O_2_ by the photogenerated holes, showing optimal reversibility in the oxidation reaction and a reduction on the charging voltage up to 2.65 V. CNTs, without any QDs on the surface, were tested demonstrating no reversibility whatsoever. The stability and efficiency of the CdSe QDs can be attributed to the surface passivation effect of ZnS, making them more robust (useful in aggressive environments with oxidation and reduction reactions), hindering recombination of photogenerated electrons and holes as well as increasing photoluminescence [286], [287], [288].

The ZnS@CNT composite was proposed by Liu et al. for solid-state Li-ion O_2_ photobatteries [289] to address the overpotential issue present in these systems. Even though the CB of ZnS and CdSe@ZnS QDs give them the same theoretical charging voltage under illumination, the actual charging voltage of the ZnS system was 2.08 V (2.65 V for that of CdSe@ZnS QDs), presenting an electric energy efficiency of ∼113%. The charge voltage remains stable at 2.2 V after 50 cycles (see Figure 8J and K). The diffraction peaks of ZnS revealed the hexagonal structure and SEM images (Figure 8L) showed CNTs uniformly covered with spherical grain-like nanoparticles of ∼70 nm of diameter. They offer a large surface area that boosts the photocatalytic oxidation of Li_2_O_2_. In 2020, Ren et al. tested GeSe NPs as photoactive material in Li-ion photobatteries [290]. GeSe is a chalcogenide with visible light-absorbing properties, large absorption coefficient (>104 cm^−1^), and high electrical conductivity [291, 292]. GeSe nanostructures have already been studied in non-photo Li-ion batteries. They exhibit a high diffusion rate of lithium ions without impacting the GeSe nanostructure, particularly when they form nanoparticles with a high surface/volume ratio [293], [294], [295]. The synthesized GeSe nanoparticles had a wide size distribution (100–300 nm) with an orthorhombic crystal structure composed of folded layers that interact by van der Waals forces, where atoms within the layer are covalently bound to three neighbors [296]. The NPs were uniformly distributed on the surface of the PE. The electrochemical characterization of the PE was investigated in a Li/GeSe half-cell, where the first discharge step revealed the formation of SEI, Ge, and Li–Ge alloys [292, 293, 297] that decrease the capacity during the charging process. However, no capacity loss is observed after the second cycle, with a Coulombic efficiency of 99.8% over 100 charging/discharging steps. When tested in a two-electrode configuration with LiClO_4_ as the electrolyte, the battery suffers from self-discharging. Tian et al. proposed SnS_2_ arrays on Ti mesh as photocathode in a two-electrode redox flow system [298]. SEM and TEM images (see Figure 8M and N) confirm its nanoflower- and nanowall-hierarchical morphology, with a well-layered structure because of the presence of weak van der Waals interactions between sheets (with an interlayer distance of 0.59 nm). These unique vertically oriented SnS_2_ arrays provide a large surface area that facilitates the contact with the liquid electrolyte and enhances light harvesting efficiency and charge transport. As a result, the PE shows good cycling capability without any apparent decay in voltage or current.

Another example of chalcogenide-based photoactive nanomaterial is molybdenum disulfide (MoS_2_), reported by the group of de Volder in 2021 [299]. They used the same recurring strategy by introducing an electron blocking layer between the photoactive material and a charge collector (carbon felt) to reduce the recombination of the photogenerated holes and electrons [59, 234]. The better light-harvesting properties, higher cycling stability and less toxicity of MoS_2_ compared to vanadium oxides make it a more suitable alternative as photoanode in Zn–air photobatteries. The reason why MoS_2_ is more stable after charging and discharging cycles is that the intercalation of Zn ions does not affect the semiconducting 2H phase (hexagonal structure, where Mo atoms are coordinated by six surrounding S from the upper and lower layer) of MoS_2_ dense nanosheets. This is the key to the photocharging process (versus 1T phase, metallic) [300], [301], [302]. Only after 500 cycles, it starts to present some cracks and its surface becomes rougher due to the formation of Zn dendrite. This resulted in a photoconversion efficiency of 1.8 and 0.2% for 455 nm and 1 sun illumination, respectively.

A mesoporous In_2_S_3_@CNT/SS (stainless-steel mesh) was proposed as a photocathode in Li–CO_2_ batteries by Guan et al. [303]. Of the three crystalline structures of In_2_S_3_, β-In_2_S_3_ (spinel-like) is the one that behaves as an *n*-type semiconductor [304]. Its high stability, low toxicity and resistance to photocorrosion have made it an attractive candidate in photocatalysis [305], [306], [307]. In_2_S_3_ nanosheets covered uniformly CNT surface with an ultrathin layer of 2–5 nm in thickness. Its porous structure (with a pore size of 3–10 nm) in the 3D-nest like CNT nanoforest exposes many active sites for photocatalytic reactions between the electrode and the electrolyte, increases light absorption and promotes charge separation. Charge and discharge voltages are 3.14 and 3.20 V, respectively under illumination. As a result, the round-trip efficiency is 98.1%, compared to 70.7% in the dark. This demonstrates the beneficial photocatalytic properties of In_2_S_3_. The mechanism of the discharge step under illumination is slightly different from Li–O_2_ batteries. The authors proposed the formation of In^+^ when the photogenerated electrons travel to the surface of the composite. Afterward, CO_2_ is reduced by In^+^ and they form In^3+^–C_2_O_4_^2−^ adduct, confirmed by a vibration peak at 132 cm^−1^ (indication of the In–O bond) observed in Raman spectrum and another peak at ∼444.2 eV corresponding to In^+^ in XPS analysis. In addition to the above mentioned photoactive materials, there has been a recently-published report of a double metal chalcogen-based material formed by nickel and cobalt in two different oxidative states (+2 and +3): NiCoS_4_ (NCS) [308]. Compared to its oxygen-based analogue (NiCoO_4_), NCS has 100 times more conductivity, more flexible structure (favorable for ion intercalation) and more suitable redox properties because of the lower electronegativity and larger atomic radius of sulfur [309, 310]. It also has a better response to visible light absorption, which gives NCS the needed driving force to carry out the OER reaction efficiently with lowered overpotential. The synthesis was performed with the help of a template that provides a spinel structure with microsphere-like morphology that exponentially increases the number of active sites for the heterogeneous photocatalytic reaction. However, the round-trip efficiency is 68.8%, much lower than what was obtained with the In_2_S_3_-based PE, suggesting deposition of a thin layer on top of the CNT charge collector might be more beneficial [311].

### 3.4 Perovskite-based nanomaterials

**Figure 9:**
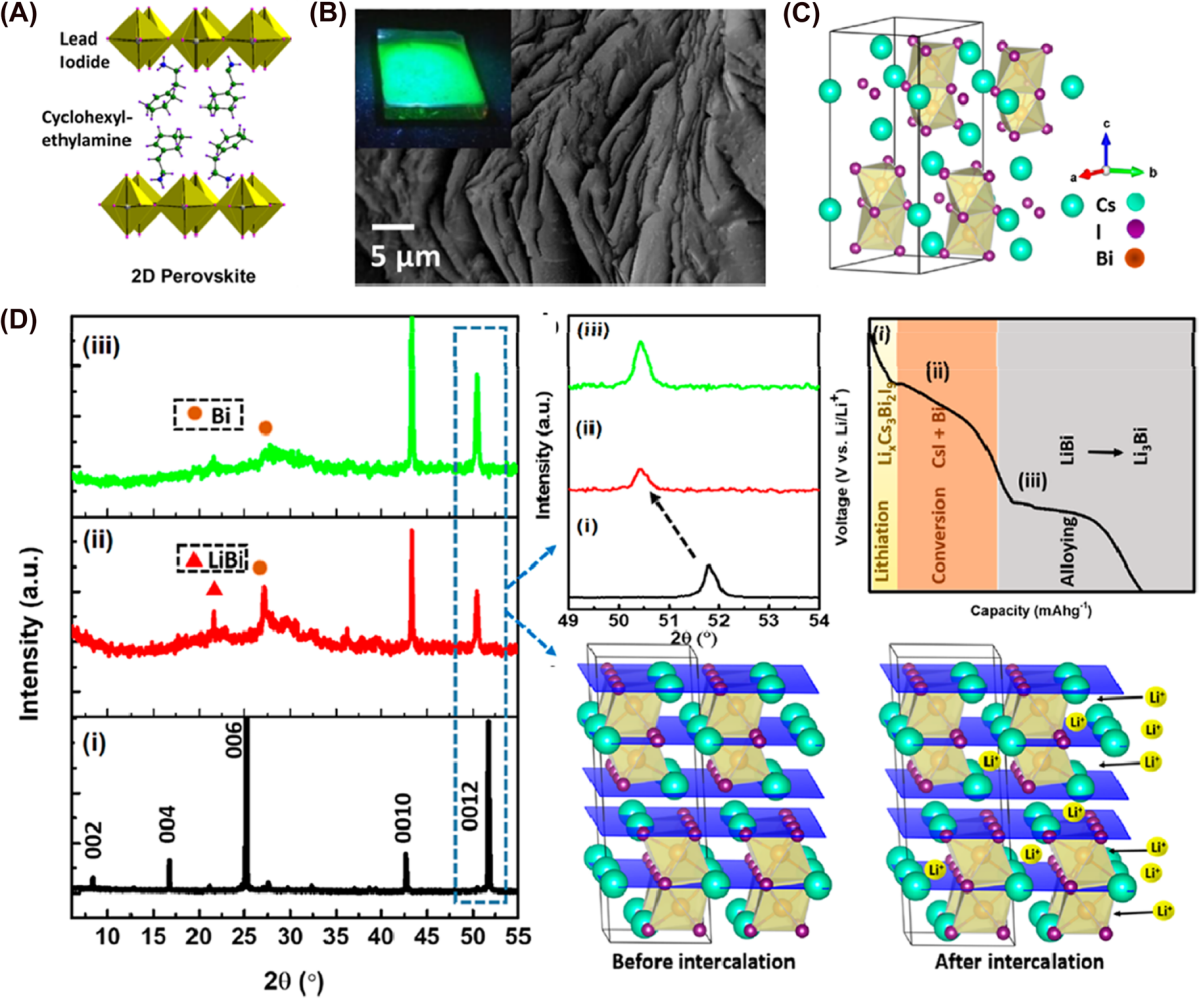
Perovskite-based photoelectrodes. (A) Representation of the crystal structure of 2D CHPI. (B) SEM image of 2D CHPI perovskite PE taken at 45° tilt. The inset corresponds to a photoluminescence (PL) image of the perovskite film (*λ*_ex_ ∼ 300 nm) [293]. Copyright 2018, American Chemical Society. (C) Crystal structure of Cs_3_Bi_2_I_9_. (D) XRD patterns of Cs_3_Bi_2_I_9_ at various stages of a discharge cycle (i, ii, iii) with a magnified view of the characteristic peak (0012); the discharge curve showing the three phases of discharge and the crystal structure before and after Li^+^ intercalation [294]. Copyright 2021, American Chemical Society.

Perovskite lends its name to the class of compounds that have the same type of crystal structure as CaTiO_3_ [312]. Organic-halide perovskites have received tremendous attention over the last decade in the field of photovoltaics due to their unique properties which include tunable bandgap, high charge carrier mobility, broad absorption spectrum, long charge diffusion lengths, and high defect tolerance [313, 314]. Metal-halide-based perovskite solar cells have achieved high power conversion efficiencies of up to 25.5% [315, 316]. More recently, some publications have shown that perovskites can be also used in Li-ion or Li–air batteries [317], [318], [319], [320]. However, it was not until 2018 that the first perovskite-like material was used in photo-rechargeable Li-ion batteries. Ahmad et al. reported two polycrystalline metal-halide-based 2D perovskites as energy conversion and storage photoanodes: cyclohexylethylamine lead iodide (C_6_H_9_C_2_H_4_NH_3_)_2_PbI_4_ (CHPI) and its bromine analogue, cyclohexylethylamine lead bromide (C_6_H_9_C_2_H_4_NH_3_)_2_PbBr_4_ (CHPB) (Figure 9A) [321]. The organic cations sandwich the inorganic monolayers formed by PbI_6_^4−^ octahedral. This layered structure enhances stability compared to 3D perovskites and absorbs/diffuses Li ions effectively [322, 323]. The drop-casting deposition method was chosen so the nanostructures would grow vertically on top of the FTO substrate, allowing favorable charge transfer, increasing the porosity and promoting more efficient light interactions. Figure 9B shows the SEM image of 2D perovskite nanoplatelets that are 8–10 μm high and 320 nm thick. The energy is stored in the perovskite by ion intercalation and conversion processes [324]. Because of the 2D perovskite structure, Li^+^ ions can migrate and interact with the perovskite throughout different sites (*i.e.* with the ring or functional group of the organic molecule, in the bulk or the surface) [325] in a more efficient way when compared to 3D perovskites, leading to greater device performance. It was noticed that *d*-spacing between the interlayers measured by XRD moves from 17 to 18 Å when the discharge voltage is under 1.85 V. This proves that there is an efficient transport of Li^+^ ions between the 2D perovskite layers that contribute to the capacity [325]. In addition, it also demonstrates that the discharge processes modify the perovskite structure when the Li^+^ ions occupy empty spaces in the perovskite crystalline structure. It is observed that the intercalation occurs at 1.85 V, while the conversion reaction proceeds at 0.3 V. The latter one is responsible for the stability issues by generating SEIs and lead metal that can form an alloy with Li. This results in an irreversible change of the morphology of the layer [326] and an important decrease in the capacity/overall efficiency of the device (which was 0.034% in the best case). Because of this and the toxic nature of Pb, other metals (such as Sn or Bi) need to be leveraged for energy storing systems.

Two more recent examples of lead-free perovskite have been reported using Cs_2_AgBiBr_6_ double perovskite [116] and Cs_3_Bi_2_I_9_ nanosheets [327] as photo-active materials. The double perovskite fulfills the basic requirements to be an adequate photoactive material, but it lacks stability since solvent crossover between compartments will eventually dissolve the perovskite. In addition, the formation of AgBr after cycling changes the morphology of the Cs_2_AgBiBr_6_ film and it has oxygen-induced self-discharge issues, a concern in aqueous batteries with low O_2_ tolerance [116, 328, 329]. Cs_3_Bi_2_I_9_ is a lead-free, all-inorganic halide perovskite that forms zero-dimensional (0-D) nanocrystals (NCs) with hexagonal packing structure where [Bi_2_I_9_]^−3^ forms octahedral clusters surrounded by Cs^+^ ions, as can be seen in Figure 9C. The champion photobattery (photoconversion efficiency of 0.43% for the first cycle) includes carbon felt as the current collector and it was shown that it can be charged without any external current. Under illumination, the photogenerated current compensates for the discharge current at the studied discharge rate. Nevertheless, a drop in the discharge capacity is observed after the first cycle. If the degradation of the photobattery follows the same mechanisms as the non-photo Li-ion reported using the same perovskite as an anode, the loss in capacity is due to SEI, the formation of Li–Bi alloys and the conversion of Bi^3+^ into Bi^0^. The latter causes irreversible changes in the morphological structure of the perovskite that can be detected by XRD. The authors observed metallic Bi^0^ and a shift of 1.4° to a smaller angle in the (0012) plane peak, indicating an increase in the *d*-spacing due to lithium intercalation (Figure 9D). More studies are needed to fully understand degradation pathways before the enhancement of photobattery stability is possible.

### 3.5 Organic-based photoactive materials

**Figure 10:**
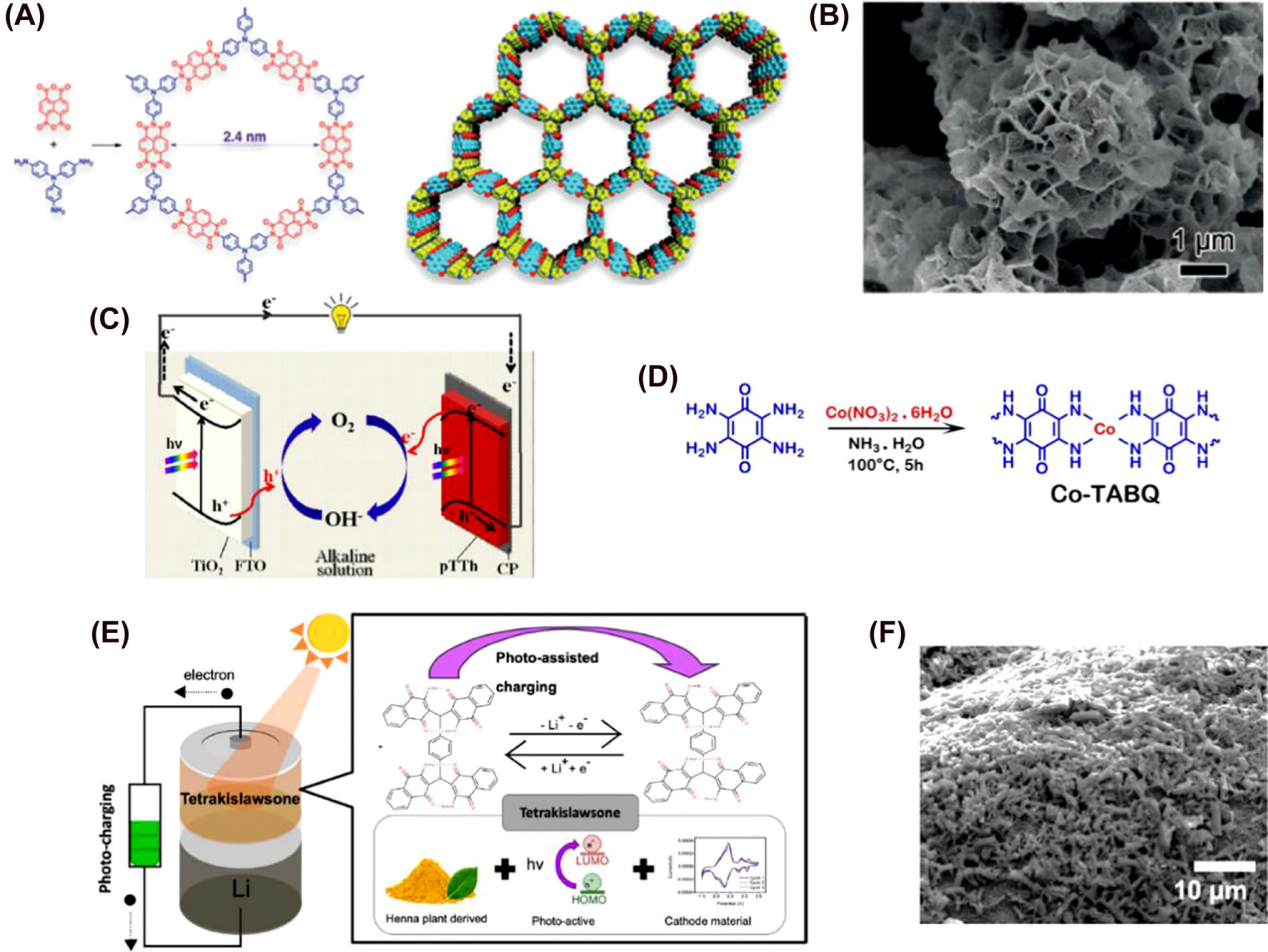
Organic-based photoelectrodes. (A) Condensation reaction and extended structure of NT-COF showing hexagonal mesoporous channels with the alternating columns of TPA (yellow) and NDI (cyan) subunits [56]. Copyright 2018, John Wiley and Sons. (B) SEM image of nanofibers of carbon paper coated with pTTh [96]. Copyright 2019, John Wiley and Sons. (C) Illustration of the dual PE photoelectrochemical cell [315]. Copyright 2019, John Wiley and Sons. (D) Synthesis and structure of Co-TABQ [316]. Copyright 2021, American Chemical Society. (E) Schematic representation of LiTKL photo-rechargeable battery along with the molecular structures of LiTKL and TKL. (F) SEM image of TKL PE [317]. Copyright 2021, American Chemical Society.

The main advantage of using organic-based materials is being able to tune their physicochemical, optical, and electrical properties through simple synthetic steps. These modifications open up an immense number of possible materials. There are some examples of small organic molecules, polymers, metal–organic framework (MOFs), and covalent organic frameworks (COFs) that have been used in rechargeable batteries [330], [331], [332], [333], but not all of them have light absorption and charge storage abilities. Lv et al. reported the first and only COF utilized as a bifunctional electrode to date, in solar rechargeable Li-ion batteries. NT-COF consists of an extended aromatic electron-deficient unit based on 1,4,5,8-naphthalenediimide (NDI) and a triphenylamine (TPA) moiety as an electron-rich unit (Figure 10A) with absorption in the UV region and a broad peak between 450 and 600 nm. It forms a porous 2D trigonal crystalline structure with a hexagonal aperture of 2.4 nm. A 3.65 Å distance was elucidated through XRD for face-to-face π–π stacking of two consecutive planes. Based on these facts, intramolecular charge transfer and reversible electrochemical reactions required in Li-ion batteries are favorable. The charge and discharge voltage under illumination was 2.53 and 2.96 V respectively at 10 mA g^−1^, compared to 2.02 and 2.42 V under dark conditions. Therefore, the total battery efficiency was increased up to 117%. Another study showed the utilization of polytrithiophene (pTTh) deposited on carbon paper as photoelectrode in Zn–air batteries [120]. The SEM image in Figure 10B displays the hierarchical spherical structures of 4–5 μm in diameter that are kept together by nanosheets of 20–50 nm in thickness. The rounded structures are beneficial for light capturing and efficiently boost the electrolyte and oxygen diffusion and transport. This has favorable effects on the ORR reaction during the charging process. Although the photoactive material exhibited physicochemical stability over charging and discharging cycles for 64 h without showing any voltage decay, additional efforts are required to increase the photogenerated current.

Zhang et al. also published a pTTh photocathode in a fuel-free photoelectrochemical cell (PEC) with TiO_2_ for the water oxidation reaction [334]. This cell produces water and oxygen instead of CO_2_ (which makes it environmentally friendly) and electricity concurrently, being the first fuel-free PEC cell reported with these beneficial characteristics. Figure 10C depicts the working principles of the dual-PE PEC cell. It is based on the circulation of OH^−^ and oxygen in alkaline media. When both photoelectrodes are illuminated, the photo-to-electric energy conversion efficiency is 0.18% under air atmosphere and tandem illumination. pTTh PE benefits from the absorption of TiO_2_ that blocks the UV light, so it does not reach the organic polymer and thus the photodegradation of pTTh can be avoided completely. pTTh nanostructure on carbon paper is similar to that is described in the previous example, with a 43 μm film thickness.

Lv et al. published a metal–organic polymer (MOP) based on tetraaminobenzoquinone (TABQ) as the organic ligand and Co^2+^ as the metal (Co-TABQ). It has a bandgap of 2.2 eV and an absorption edge at 585 nm [335]. Figure 10D reveals the SEM image of Co-TABQ nanosheets (100–200 nm) covering the surface of carbon paper uniformly. The adsorption–desorption isotherm type IV and the SEM image suggest that Co-TABQ is a nanoporous material. This is beneficial for having more active sites and better light absorption. Under illumination, the round-trip efficiency is 94% for a two-electrode Li-ion photobattery. Charge and discharge voltage values remain almost constant over 50 cycles, demonstrating good stability of the MOP, which is also confirmed by SEM images taken during the charge and discharge processes showing the as-synthetized hierarchically porous structure. This means that Li_2_O_2_ is formed, deposited on the surface of Co-TABQ and oxidized into Li^+^ and O_2_ during the charge of the PBAT.

An organic small molecule was for the first time reported as bifunctional PE in photo-rechargeable batteries in 2021 [336]. It is environmentally friendly since it does not contain any inorganic metal and can be extracted from the henna leaf. It is called tetrakislawsone (TKL) and has the capability of storing energy chemically as a result of the reversible chemical coordination of ketone groups with Li^+^ ions. This process occurs through the electrochemical reduction of the carbonyl and/or the covalent modification of hydroxyl, as shown in Figure 10E. The molecular structure plays a key role; the nearby functional groups and the 3D orientation of the molecule will influence the stability and the storing ability [337]. As a result of the polyaromatic quinones TKL contains in its molecular structure, it can effectively delocalize the charge carriers within the molecule. Under illumination, the photogenerated electrons will travel to the anode, reducing Li^+^ into metallic Li, while holes assist with the delithiation and charging process. TKL is deposited on top of the carbon-coated stainless-steel mesh, showing nanosize crystal-like textures (see Figure 10F). This work paves the way for the utilization of organic molecules in photo-rechargeable storing systems by carefully designing the molecular structure with the proper bandgap that allows light absorption and functional groups that interact with metal ions.

### 3.6 Carbon nitride-based nanomaterials

**Figure 11:**
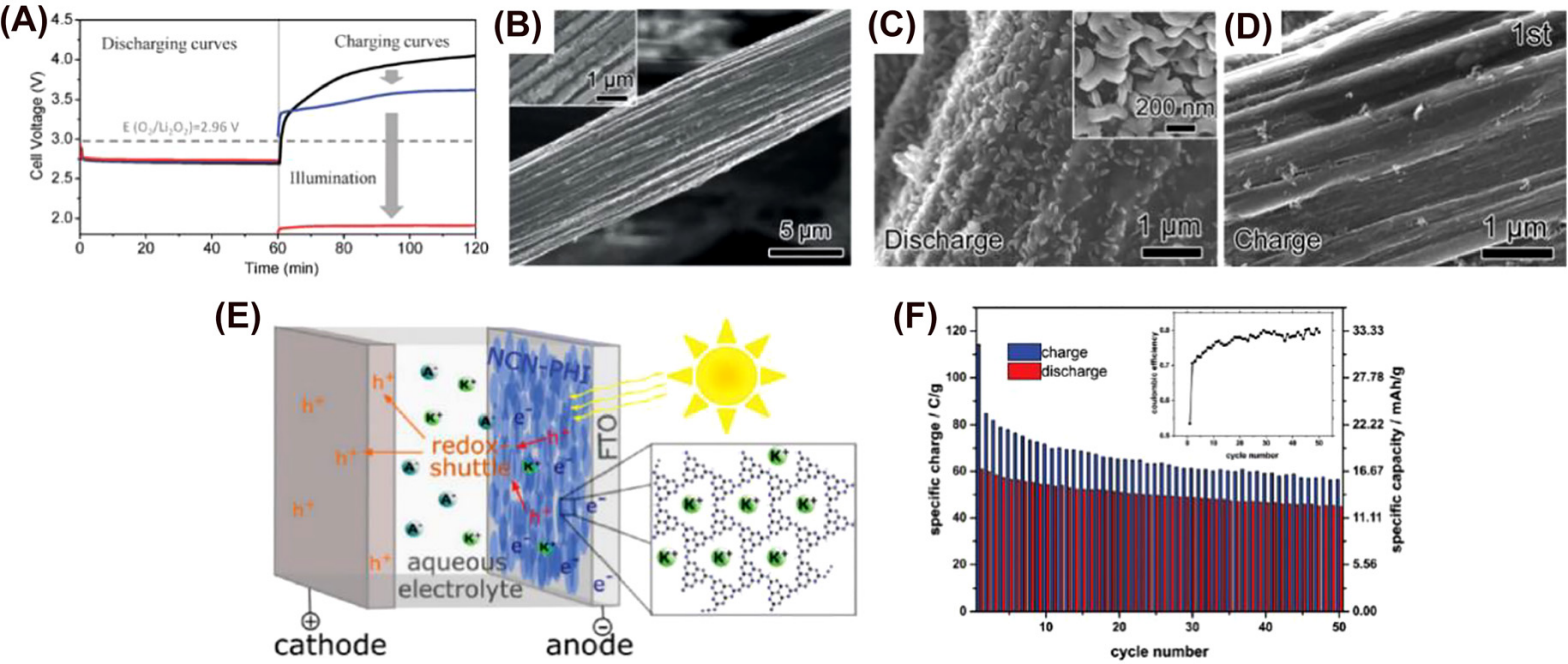
Carbon nitride-based photoelectrodes. (A) Charging–discharging curves of g-C_3_N_4_-based Li–O_2_ battery with (blue line), without (black line) the I^-^ ion redox mediator; and the photoassisted rechargeable Li–O_2_ battery (red line) [91]. Copyright 2015, Royal Society of Chemistry. (B) SEM of C_3_N_4_/carbon paper PE. Inset is the enlarged image (C) and (D) SEM images of PE after the first charge and discharge cycle [90]. Copyright 2019, John Wiley and Sons. (E) Photo-rechargeable solar battery based on NCN-PHI PE. (F) Charge stored and extracted during 50 cycles. The inset corresponds to the Coulombic efficiency versus the cycle number [328]. Copyright 201, John Wiley and Sons.

Graphitic carbon nitride (g-C_3_N_4_) is a metal-free semiconductor first reported by Wang et al. in 2009 as a photocatalyst for H_2_ evolution from water splitting [338]. Since then, it has attracted immense attention from the scientific community due to its outstanding characteristics that include facile synthesis from low-cost materials, environmental friendliness, moderate bandgap (∼2.7 eV), natural abundance, strong redox capability, high active surface, unique layered structure, and good photo- and physico-chemical stability [339], [340], [341], [342]. It consists of tri-*s*-triazine rings as building units with high nitrogen content and abundant triangular nanopores/defects, which provide a substantial number of active sites for ion adsorption and redox reactions [342, 343]. Its low CB makes it a more suitable photocatalyst in Li–O_2_ system compared to TiO_2_ or ZnO because it is closer to the redox potential of Li/Li^+^ and its thin layered structure ensures rapid diffusion of oxygen into the electrode. The first publication including g-C_3_N_4_ as bifunctional PE was reported by Liu et al. for a photo-assisted rechargeable nonaqueous Li–O_2_ battery [115]. The simultaneous oxygen electrode and PE is formed by growing g-C_3_N_4_ on carbon paper. g-C_3_N_4_ acts as a photocatalyst and as an ORR catalyst. Photo-excited holes oxidize I^−^ into I_3_^−^ ions, which will be reduced back to I^−^ when Li_2_O_2_ is oxidized to O_2_. Under illumination, the charging voltage is reduced to 1.9 V, addressing the overpotential issue (4–4.5 V) present in this type of battery (Figure 11A) [54, 344], giving an energy-conversion efficiency of ∼140%. Two more publications (one from the same group and the other from Zhuo et al.) using the same PE configuration have been published showing similar results [69, 84]. g-C_3_N_4_ forms a uniform and high-density layer on the porous carbon paper electrode (see Figure 11B) and exhibits a clear 2D/2D contact with it, which is beneficial for interfacial electron transfer [340]. This coating structure also provides sufficient space for the deposition of Li_2_O_2_ and allows an efficient hole transport from g-C_3_N_4_ to I^−^ during the discharge process. Figure 11C and D shows SEM images of the PE surface in photo-assisted charging and discharging process. Li_2_O_2_ particles are deposited uniformly over the surface of the semiconductor layer during battery discharge, and reversibly decomposed upon photocharging. The stability of g-C_3_N_4_ was measured over 70 photo-assisted charge and galvanostatic discharge cycles. No changes were apparent, demonstrating a stable electrode structure and maintenance of almost constant charging and discharging voltages over the cycles.

Another example using g-C_3_N_4_ was reported by Zhu et al. In this case, g-C_3_N_4_ had nitrogen defects decorated with plasmonic Au NPs [345]. Defects help promote photocatalytic OER reaction by increasing the number of active sites on its surface. Plasmonic Au NPs with an average size of 30 nm reduce electron–hole recombination and, more important, widen the light absorption up to NIR region of the PE [339]. The two-electrode system yielded an energy efficiency of 92.5% after 50 cycles, mainly attributed to the hot electrons transferred to g-C_3_N_4_ from the Au NPs. Podjaski et al. proposed a solar battery half-cell by using semicrystalline 2D carbon nitride as a light harvesting material with the capability of storing electrons for hours in the form of a long-lived photo-reduced state, separating in time the light absorption and catalytic conversion processes [346]. They synthetized a 2D cyanamide (NCN^−^)-functionalized polyheptazine imide (NCN-PHI) shown in Figure 11E. NCN-PHI is deposited on top of FTO substrate in form of exfoliated nanosheets, yielding a hexagonal pore system nanostructure. The capacity of the NCN-PHI depends on the conjugation length of the imide bridged polymer, which is in turn influenced by the synthetic conditions [347]. The more conjugation it has, the more it can trap electrons on the heptazine rings and delocalize these negative charges across the polymer backbone in the form of stable π-radicals, visible by their blue color [348]. When the holes are extracted to the CE with the help of a redox shuttle having electron donor properties (4-MBA, 4-methylbenzyl alcohol), more electrons can accumulate and reach more negative states. This leads to a more negative open circuit potential and a greater number of electrons stored. The same as g-C_3_N_4_, NCN-PHI nanosheets present high electronic stability over 50 cycles, maintaining 75–80% of Coulombic efficiency (Figure 11F). Another important aspect that is involved in the accumulation is their interaction with alkali ions (provided by the electrolyte) that diffuse into and through the structural pores of NCN-PHI.

### 3.7 Other photoactive nanomaterials

Several photoactive materials have been reported over the last years in photo-assisted batteries. For example, Thimmappa et al. published a chemically rechargeable photobattery that could be recharged even under ambient light, without applying an external bias. TiN acts as the photoactive material and, KFe[Fe(CN)_6_] and sodium persulfate (Na_2_S_2_O_8_) as the active species in the battery [349]. TiN is well-known for its broad absorption spectrum up to 650 nm, high electrochemical stability, corrosion resistance and decent conductivity. Oxynitride (TiON) and some traces of TiO_2_ can be found on the surface of TiN due to oxidation. TiON has light absorption in the visible and UV regions, good conductivity and chemical stability so it contributes positively to the generation of electron–hole pairs and charge separation [350, 351]. Since TiN is not involved in any electrochemical reaction it does not present signs of deterioration over charge–discharge cycles nor gets dissolved in the electrolyte.

**Figure 12:**
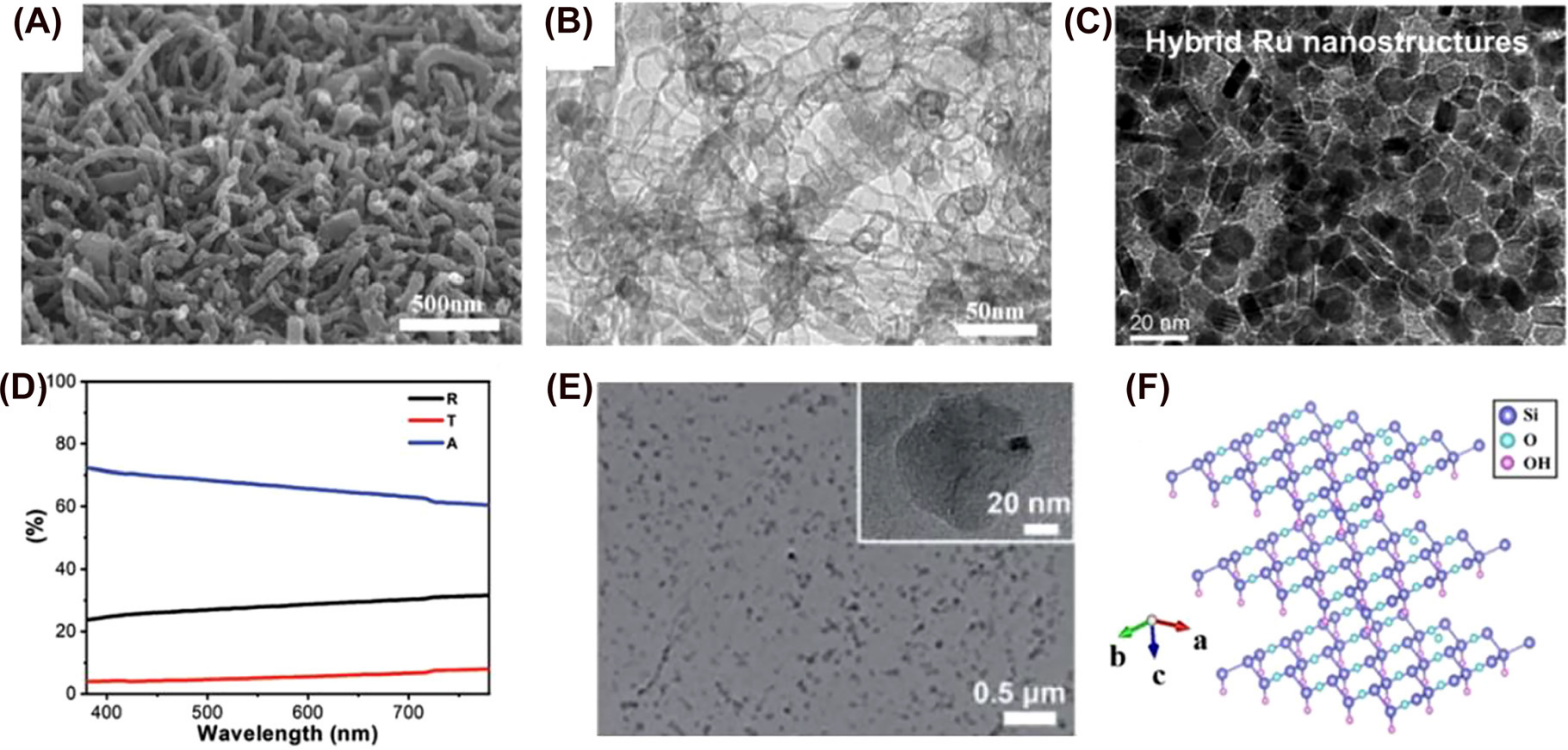
Photoelectrodes based on other photoactive nanomaterials. (A) and (B) correspond to SEM and TEM images of Ni_12_P_5_@NCNT hybrid [336]. Copyright 2018, Elsevier. (C) TEM image of single-layer plasmonic Ru nanostructures assembled on the porous cathode. (D) Reflection (black line), transmission (red line), and absorption spectra (blue line) of single layer [337]. Copyright 2020, Royal Society of Chemistry. (E) TEM image of SiC/rGO PE. Inset image is a magnified TEM image [338]. Copyright 2020, Royal Society of Chemistry. (F) Crystal structure of siloxane NSs [339]. Copyright 2021, John Wiley and Sons.

Another example is the work published in 2018 by Lv et al. where they synthetized a Ni_12_P_5_@NCNT (nitrogen-doped carbon nanotubes) catalyst as photoanode in Zn–air photobattery [352]. Ni_12_P_5_@NCNT forms a p–n heterojunction and when it is exposed to light, photogenerated holes are transferred to Ni_12_P_5_ surface to oxidize water and the electrons move to the NCNT surface to reduce oxygen. The bamboo-like CNTs are decorated with Ni_12_P_5_ NPs that are highly dispersed (Figure 12A and B). CNTs construct 3D networks that are greatly favorable for fast electrons transfer and help to prevent aggregation of Ni_12_P_5_ NPs [353]. Brunauer–Emmett–Teller (BET) analysis displayed a hysteresis loop distinctive of a mesoporous material with a surface area of 120.3 m^2^ g^−1^ and an average pore size of ∼3.6 nm. This proves that the actives sites are sufficiently exposed to the electrolyte to promote the photocatalytic reactions [354]. The Ni^*δ*+^ peak at 853.8 eV in high-resolution XPS analysis was red-shifted compared to the peak for Ni_12_P_5_ NPs alone (852.6 eV) [355]. This demonstrates the strong coupling effect between NCNT and Ni_12_P_5_ NPs, which facilitates the electron transfer to Ni_12_P_5_ NPs. However, the round-trip efficiency obtained was only 64.2%. In 2020, Song et al. published an all-solid-state Li–air photobattery that can operate at a very low temperature (−73 °C) [356]. The working mechanism involves the deposition of a very thin layer of Ru NPs (5–20 nm of size) with surface plasmon resonance properties, as Figure 12C and D demonstrates. The photogenerated carriers formed at the electrode release the energy in form of heat dissipation, resulting in light-induced thermal heating of the battery, increasing the charge storage and cycling life [357, 358].

**Figure 13:**
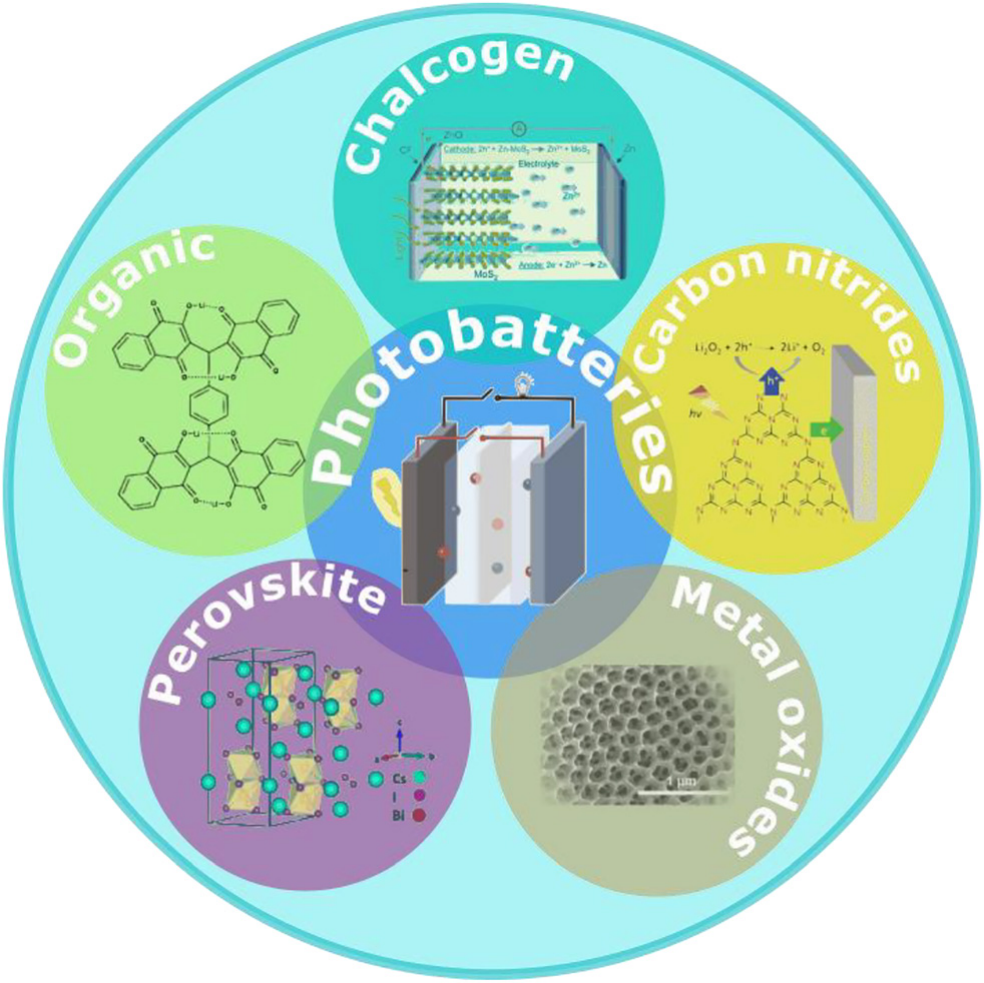
Schematic diagram summarizing the main contents of this review, *i.e.*, main groups of nanomaterials that are used as PE in PBATs. Copyright 2021, American Chemical Society and 2016, Royal Society of Chemistry.

Two graphitic-like nanomaterials were reported for Li–CO_2_ batteries. The first consisted of a hybrid photocathode employing SiC grown on rGO (SiC/rGO) [68], where SiC was synthetized *in situ* on the rGO surface. They adopted “island-in-sea-like” morphology (see Figure 12E) with cubic structured crystals. The CO_2_ adsorption is favoured by 2D nanosheets rGO as well as the Si–OH bonds present on the surface of SiC. The photoelectrode remains stable over 20 cycles, maintaining its initial morphology. In 2021, 3-nanosheet thick ultralarge size siloxene (2.05 nm) was proposed as a photoactive material with energy storing properties by C. Jia et al. [67]. It confers an impressive discharge/charge voltage retention (98 and 93% respectively), round-trip efficiency of 185% in the first cycle that remained at 170% after 100 cycles. Figure 12F shows siloxene nanosheets with a graphene-like structure formed by S_6_ rings connected by Si–O–Si bridges with surface-terminated functional groups such as –O, –H and –OH, corresponding to the Kautsky structure [359, 360]. Its structure, optical bandgap of 2.48 eV, high surface area and low recombination rate allow high photocatalytic activity yielding an impressive discharge capacity of 1170 mA h g^−1^ after 12 h of illumination (at 0.75 mA cm^−2^). The formation of Li_2_O_2_ was fully reversible, demonstrating great stability of the PE. Materials with 2D nanosheet morphology (such as SiC, siloxane, g-C_3_N_4,_ and Cs_3_Bi_2_I_9_ nanosheets) have proven to yield higher capacity values due to the high electrolyte insertion in their structure [361].

## 4 Challenges and outlook

Currently, photo-rechargeable batteries are still in an early experimental stage, presenting numerous problems and challenges. The maximum overall efficiency in a two-electrode photobattery achieved to date was 9% (see Table 2, where a summary with all the photoactive nanomaterials and their performance is described). Based on these values, we can conclude that the commercialization of integrated photobatteries remains feasible but not in their current state. In this section, we present the main challenges standing in the way of developing efficient PEs. Insights into the most efficient nanomaterials will be offered for developing ideas in the future rational design of PEs (Figure 13).

**Table 2:** Classification and main characteristics of described two-electrode integrated photo-rechargeable batteries.

Photoanode active material	Photocathode active material	Type of integrated photobattery	Capacity (mA h g^−1^)	Cycling number	*η*_conversion_ (%)	*η*_battery_ (%)	Need of external bias	Reference
Dye-sensitized (Z907)	–	SFB	–	4	–	–	Yes	[132]
–	Dye-sensitized (N719)/LiFePO_4_	Li-ion	340	15	0.06–0.08	–	No	[83]
Dye-sensitized (N719)/TiO_2_	–	Li-ion	209	10	–	–	Yes	[133]
–	Dye-sensitized (N719)/TiO_2_	Li–S	906	30	–	–	Yes	[135]
–	Li_*x*_TiO_2_ mesoporous film	Li-ion	243	–	–	–	–	[163]
–	Li_*x*_TiO_2_ NPs	Li-ion	–	–	0.5	–	Yes	[165]
–	TiN/TiO_2_ NWs@carbon clothe	Li–O_2_	500	120	–	94	Yes	[179]
–	Fe_2_O_3_ nanorods/TiO_2_ nanoarrays@carbon clothe	Li–O_2_	–	100	–	86	Yes	[180]
Ultralong TiO_2_ nanobelts	–	SFB	–	–	–	–	Yes	[168]
–	TiO_2_ nanotubes	Na-ion (Seawater)	–	30	–	–	–	[174]
–	TiO_2_ nanorod arrays/Au NPs	Li–O_2_	–	200	–	–	Yes	[181]
–	α-Fe_2_O_3_ nanorods	Zn–O_2_	598.7	75	–	70.3	Yes	[86]
–	α-Fe_2_O_3_ nanorods	Li–O_2_	–	41	–	81.2	Yes	[85]
α-Fe_2_O_3_ NPs	–	RFB	∼275	10	0.05	–	No	[195]
					0.08%			
–	α-Fe_2_O_3_ NPs	Li–I_2_	∼192	28	–	95.4	Yes	[199]
–	WO_3_ nanowire array	Li–O_2_	–	100	–	81.4	Yes	[220]
–	g-C_3_N_4_ decorated WO_3_ nanowire array	Li–O_2_	–	100	–	–	Yes	[223]
–	V_2_O_5_ nanofibers	Zn–air	∼370*	25	1.2	–	No	[59]
–	V_2_O_5_ nanofibers	Li-ion	∼161	175	2.6*	–	No	[66]
					0.22**			
–	VO_2_ nanorods	Zn-ion	∼315	1000	0.18*	–	No	[234]
–	LiV_2_O_5_	Li-ion	185	50	9	–	Yes	[65]
MoO_3_ platelets	–	Na-ion	190	2	–	–	No	[248]
–	Spinel-type Co_3_O_4_	Zn–air	769	17	–	60	Yes	[257]
CdS clusters	–	RFB	–	–	2.12	–	Yes	[265]
CdS cauliflower-like features	–	RFB	–	8	–	–	No	[269]
–	CdS NPs	Li–S	792	10	–	–	Yes	[119]
CdS-sensitized WO_3_	–	Li-intercalation	19.5	10	–	–	Yes	[113]
CdS-sensitized (TiO_2_)-WO_3_	–	Na-ion	18.1	10	0.3	–	Yes	[280]
–	CdSe/ZnS QDs	Li–O_2_	–	100	–	–	Yes	[283]
–	ZnS	Li-ion O_2_	–	50	–	113	Yes	[289]
–	GeSe	Li-ion	670	100	–	–	Yes	[290]
SnS_2_ arrays	–	RFB	–	10	0.11	–	Yes	[298]
–	2D MoS_2_ nanosheets	Zn-ion	340*	200	1.8*	–	No	[299]
					0.2**			
–	Porous In_2_S_3_	Li–CO_2_	–	30	–	98.1	Yes	[303]
–	Urchin-like NiCo_2_S_4_ microspheres	Zn–air	734	60	–	68.8	Yes	[308]
CHPI/CHPB (2D perovskite platelets)	–	Li-ion	100/410	12/–	0.034/–	–/–	Yes/–	[321]
Cs_2_AgBiBr_6_	–	RFB	0.25	20	–	–	Yes	[116]
–	Cs_3_Bi_2_I_9_ nanosheets	Li-ion	975	150	0.43	–	No	[327]
–	2D porous NT-COF	Li-ion	100	5	–	117	Yes	[80]
–	pTTh nanosheets	Zn–air	–	5	–	–	Yes	[120]
TiO_2_ nanorods	pTTh	Non-metal anode–air	–	–	0.18–0.22	–	No	[334]
–	Co-TABQ nanosheets	Li–O_2_	–	50	–	94	Yes	[335]
–	TKL	Li-ion	332	12	–	–	Yes	[336]
–	g-C_3_N_4_	Li–O_2_	–	50	–	142	Yes	[115]
–	g-C_3_N_4_	Li–O_2_	600	70	–	140	Yes	[69]
–	Layered C_3_N_4_	Li–O_2_	–	10	–	95.3	Yes	[84]
–	N_v_-g-C_3_N_4_ + Au NPs	Li–O_2_	–	50	–	92.5	Yes	[345]
NCN-PHI	–	Half-cell	12.1	50	–	–	–	[362]
TiN	–	Aqueous nonmetal-ion	77.8	100	–	91.8	No	[349]
–	Ni_12_P_5_ NPs	Zn–air	640	500	–	64.2	Yes	[352]
–	Hybrid Ru NPs	All-solid-state Li-ion***	470	15	–	–	Yes	[356]
–	SiC nanosheets	Li–CO_2_	4996	20	–	–	Yes	[68]
–	Siloxene nanosheets	Li–O_2_	1170	100	–	185	Yes	[67]

*At 455 nm illumination. **At 1 sun illumination. ***At −73 °C.

Low efficiencies of PBATs are due to a variety of factors including low light absorption of nanomaterials, high charge recombination within the photoactive material and/or heterojunctions, low storage capacity and cycle reversibility. The energy levels of the photoactive material must allow broad absorption in the visible range of the spectra and overcome the energy barrier for efficient photocharging in the absence of an external energy supply. However, this remains a challenge due to large overpotentials required by metal anodes, especially in Li-based batteries. Charge recombination can be diminished by rationally designing heterojunctions or nanostructures that enhance charge transfer within and among them. The storage capability and cycle reversibility depend on the nanomaterial’s nature and the working principles of the PBAT; and will be discussed later in this section. In some metal-ion batteries, the charge is stored by means of ion intercalation with a host. The limitation comes from the intrinsic diffusivity of the metal ion in the solid PE, which inhibits the intercalation/deintercalation rate and extends the charge/discharge processes. Because of their small size and shape, nanomaterials allow for a higher degree of metal insertion/removal due to the shorter diffusion distances for metal ions within the particles. Despite this, distortion of the crystal structure and volume expansion upon intercalation can be irreversible, causing deep cracks and exfoliation/pulverization of the photoelectrode. For bifunctional PEs, *n*-type semiconductors are preferred because they can accumulate electrons in the CB effectively, counterbalancing the positive charge due to metal ion insertion. However, Lou et al. demonstrated that *p*-type semiconductors (such as MoO_3_) can also efficiently harvest light and store energy by Na^+^ intercalation [248], opening new possibilities. Surface modification of the active materials with electron-rich groups can effectively decrease the metal ion insertion energy barrier by increasing the interaction with metal ions and active sites. Transition metal compounds from IV.B and V.B groups are well-known to intercalate with metal ions efficiently [102, 220]. Perovskite-based nanomaterials have shown great ion intercalation capabilities as well, but more investigation is required to understand degradation mechanisms and enhance long-term stability [107, 363], [364], [365].

Corrosion of the PE usually arises from redox reactions with the active species in the electrolyte. PBATs suffer greatly from corrosion and passivation due to the formation of a SEI (*i.e.* Li_2_O_2_) that is not reversibly dissolved and accumulates in the electrode surface, hindering the light-absorption and diminishing the electrode–electrolyte contact [37, 366]. In addition, parasitic reactions on the metal electrode surface might occur because of light-induced decomposition of the electrode. Dissolution of nanomaterials, such as BiVO_4_ or MoO_3_ also represents a critical drawback and compromises the long-term stability of the battery. The compatibility of electrode materials and redox active species along with the solvent should be evaluated in advance to ensure the stability of the device.

Photoinduced corrosion is another commonly found issue in some organic materials, like pTTh, Cu_2_O [367], or metal chalcogenides, such as CdS. It leads to an important loss in photogenerated current and Coulombic efficiency [368, 369]. For organic materials, the photodegradation is related to changes in the conjugated molecular structure. In the specific case of CdS or other chalcogenide-based nanomaterials, photogenerated holes are transported to the surface where they irreversibly promote the oxidation of S^2−^ ions into sulfur (S^0^) and/or sulfate (SO_4_^2−^) [368]. The stability and reliability of the PBATs highly depends on the lifetime of the photoactive material or photocatalyst. One possible solution is to introduce a hole scavenger in the electrolyte that prevents oxidation of the semiconductor by the photogenerated holes [280]. Another strategy involves coating the nanomaterials with a thin anti-photocorrosion layer to form heterojunction or core@shell structures, such as CdS@ZnS QDs [370]. The difficulty of this option lies in finding a highly conductive material, with high charge transfer, which however does not affect the light-harvesting and storing properties of the PE.

From a morphological point of view, nanotubes, nanowires, or nanorods are more beneficial compared to other NPs since they exhibit higher reversibility of intercalation and have enhanced specific surface area. These types of nanostructures are also beneficial because of their expansive surface area that allows more active sites to be in contact with the electrolyte and/or the active species. In addition, the increase in the surface area has a positive impact on light absorption, which can increase IPCE [168, 371, 372]. Nanorods and nanowires are more photoactive and transport charge carriers more effectively compared to thin films (hematite film made of nanoparticles, g-C_3_N_4_ and MoS_2_ nanosheets), but nanotubes enhance the redox reaction rate due to their high specific surface area [373]. This happens as a result of more extended contact with the electrolyte of nanotubes since nanowires and nanorods can easily become disconnected when they contract and expand during discharge/charge [374], [375], [376].

The morphology also influences the surface-active sites depending on the coordination number or atoms in the nanostructure. For instance, atoms from the corner of nanorods have more active sites compared to nanospheres, as a result of their lower coordination number [377]. In general, these 1D nanostructures provide rapid diffusion of charges (*e.g.* for TiO_2_ nanostructures it is around 200 times higher [378]) which leads to low recombination rates. Well-aligned or vertically oriented nanostructures are recommended for an optimal charge and ion transport efficiency since charge transport is preferred in one distinct direction, which decreases even more charge recombination. Another advantage of using 1D nanostructures is the formation of a packed interconnected network [379, 380] that was shown to improve electronic conductivity and considerably prolonged lifetime due to having a reduced number of agglomeration sites [381].

Mesoporous materials have also benefited from more rapid intercalation/deintercalation of ions, enabling them to store more energy compared to their bulk counterparts. The thin mesoporous walls confirm short diffusion paths for ion intercalation and charge transfer. Such materials are composed of micrometer-sized particles with pore size of 2–50 nm [60]. Because of their uniform and ordered porosity microstructure, the internal pores are also in contact with the electrolyte, ensuring a high surface area in contact with the photoelectrode. As a result, mesoporous materials have been found to have superior cycling capability for Li-ion batteries [382], [383], [384]. Hole-electron recombination decreases when crystallinity increases, influencing the photogenerated current within the device. Regardless, the electrochemical properties and the rate performance need to be improved for most nanomaterials. It is highly recommended to combine different nanostructures to form heterojunctions or nanocomposites that can compensate the individual flows of each material or synergistically help improve their properties. Heterojunctions and nanocomposites also promote electrolyte diffusion, charge separation, and ion transfer, improving electrochemical kinetics [151]. In addition, the exploration of different synthetic procedures might improve the optical and electrochemical properties of the nanomaterials by having good control over the defects and morphology. For instance, annealing precursors that emit gases (*e.g.* CO_2_) will tend to increase the porosity of the nanostructure. Ball-milling techniques are also recommended to increase the surface area, hence, the photocatalytic activity. Electrodes are the most expensive component of batteries, and the nanomaterials synthesis increases the cost even further. For this reason, it is mandatory to seek out novel low-cost nanomaterials or new preparation techniques including ball milling or liquid-based methods which are convenient for scaling-up commercial-sized batches. The use of plasmonic NPs helps increase light-absorption into NIR region. In general, 2D materials shorten the charge diffusion path during charge and discharge and they are suitable host materials to form heterojunctions and composites. The size and shape of the pores in 2D nanosheet-based PE materials are also critical factors that control the penetration of the electrolyte, which will influence the overall specific capacity and the charge/discharge rate capability [385, 386]. 2D materials also benefit from high surface area. As a result, they have a large number of exposed active sites that boost the electrochemical kinetics and conductivity of the PE. However, an increase of the surface area does not always mean an improvement on the performance due to the possible excessive formation of SEI layer in the surface of the PE [361, 387].

As it can be observed from the main limitations, there are still many factors that hinder high battery stability and reliability, hence, performance. Nanomaterials with high surface area and small size showed higher performance due to the increase in surface-to-volume proportion, which extend the number of active sites, interfacial charge transfer, and improve reaction kinetics with redox active species, which will enhance the catalytic activity. In this way, single atom catalysts are the direction to follow. These individual metal atoms on support materials benefit from a low coordination environment (allowing a higher interaction), quantum size effects, homogeneity of active sites, and metal–support interactions which improve charge transfer [388], [389], [390]. Having this said, durability issue needs to be addressed for this type of highly active catalysts. We would like to point out the importance of integrating researchers from a wide variety of backgrounds (such as Chemistry, Physics, Engineering, and Material Science) to work together in overcoming these limitations. Additionally, an emphasis should be given to life cycle analysis, to better assess the economic, environmental and social impacts associated with all the stages of the life cycle and components of PBATs [391, 392]. This includes the resources generation, material processing of all battery components, product manufacturing, package and distribution, use and end of life.

One critical aspect we would like to mention is the necessity of standardization of experimental conditions and characterization tests in the field of PBATs. Currently, it is very difficult to directly compare results and draw conclusions from the published literature. Without such standardization, progress cannot be guided in a rational way. Furthermore, photo-to-electric energy conversion efficiencies are not discussed in most publications on photo-rechargeable batteries. There has been a proposed guideline for Li-ion batteries that can be adapted for other fields or types of batteries [393]. For PBATs, it should include photo-to-electric energy efficiency, round-trip efficiency, charge and discharge capacity (or areal capacity) at a specific charging-rate, and charge and discharge voltage, among others. Nanomaterials tested in half-cells should also demonstrate their performance in a full cell.

Lastly, an emphasis on in-depth theoretical research focusing on voltage, current, energy and power matching of the different components of the storage system would prove beneficial to the field. This could allow for predictive studies of photobattery performance and rational design of devices.

## 5 Conclusions

To summarize, we believe that nanomaterials are the key to meaningful progress in photoelectrode research for solar rechargeable batteries. Their significance was demonstrated through relevant examples discussed in this review and their main advantages have been discussed compared to bulk materials. Future generations of photobatteries with high energy and photo-to-electric efficiency will assuredly rely on nanostructured materials. Although fabrication of a low-cost stable and efficient photoelectrode remains a challenge, projected growth in the field will eventually lead to a solution for viable commercialization. In particular, the price of Li-ion batteries will drop below $100 and lithium–nickel–manganese–cobalt-oxide (NMC)–graphite–silicon and LFP-graphite will dominate the market in the next 20 years. The integration of the photo battery in these two systems will reduce range anxiety and increase the calendar life of the photobattery. We expect important developments of these integrated systems help meet environmental and energy goals in the coming decade.

## References

[1] Schiffer H.-W. (2008). WEC energy policy scenarios to 2050. *Energy Pol.*.

[2] McManamay R. A., Parish E. S., DeRolph C. R., Witt A. M., Graf W. L., Burtner A. (2020). Evidence-based indicator approach to guide preliminary environmental impact assessments of hydropower development. *J. Environ. Manag.*.

[3] DeCarolis Joseph F., Keith David W. (2001). The real cost of wind energy. *Science*.

[4] Guangul F. M., Chala G. T. (2019). Solar energy as renewable energy source: SWOT analysis. *2019 4th MEC International Conference on Big Data and Smart City (ICBDSC)*.

[5] Kannan N., Vakeesan D. (2016). Solar energy for future world: - a review. *Renew. Sustain. Energy Rev.*.

[6] Carrillo A. J., González-Aguilar J., Romero M., Coronado J. M. (2019). Solar energy on demand: a review on high temperature thermochemical heat storage systems and materials. *Chem. Rev.*.

[7] Jurado U. T., Pu S. H., White N. M. (2020). Grid of hybrid nanogenerators for improving ocean wave impact energy harvesting self-powered applications. *Nano Energy*.

[8] Huang S., Liu J. (2010). Geothermal energy stuck between a rock and a hot place. *Nature*.

[9] Bajwa D. S., Peterson T., Sharma N., Shojaeiarani J., Bajwa S. G. (2018). A review of densified solid biomass for energy production. *Renew. Sustain. Energy Rev.*.

[10] Green M. A. (2016). Commercial progress and challenges for photovoltaics. *Nat. Energy*.

[11] Green M. A. (2001). Third generation photovoltaics: ultra-high conversion efficiency at low cost. *Prog. Photovoltaics Res. Appl.*.

[12] Lewis Nathan S. (2007). Toward cost-effective solar energy use. *Science*.

[13] Larcher D., Tarascon J. M. (2015). Towards greener and more sustainable batteries for electrical energy storage. *Nat. Chem.*.

[14] Yan N.-F., Gao X.-P. (2021). Photo-assisted rechargeable metal batteries for energy conversion and storage. *Energy Environ. Mater.*.

[15] Rugolo J., Aziz M. J. (2012). Electricity storage for intermittent renewable sources. *Energy Environ. Sci.*.

[16] Bayeh A. W., Kabtamu D. M., Chang Y.-C., Wondimu T. H., Huang H.-C., Wang C.-H. (2021). Carbon and metal-based catalysts for vanadium redox flow batteries: a perspective and review of recent progress. *Sustain. Energy Fuels*.

[17] Al Hassan M. R., Sen A., Zaman T., Mostari M. S. (2019). Emergence of graphene as a promising anode material for rechargeable batteries: a review. *Mater. Today Chem.*.

[18] Peng H.-J., Huang J.-Q., Zhang Q. (2017). A review of flexible lithium–sulfur and analogous alkali metal–chalcogen rechargeable batteries. *Chem. Soc. Rev.*.

[19] Cheng X.-B., Zhang R., Zhao C.-Z., Zhang Q. (2017). Toward safe lithium metal anode in rechargeable batteries: a review. *Chem. Rev.*.

[20] Lourenssen K., Williams J., Ahmadpour F., Clemmer R., Tasnim S. (2019). Vanadium redox flow batteries: a comprehensive review. *J. Energy Storage*.

[21] Abdel Maksoud M. I. A., Fahim R. A., Shalan A. E. (2021). Advanced materials and technologies for supercapacitors used in energy conversion and storage: a review. *Environ. Chem. Lett.*.

[22] Forouzandeh P., Kumaravel V., Pillai S. C. (2020). Electrode materials for supercapacitors: a review of recent advances. *Catalysts*.

[23] Liu H., Liu X., Wang S., Liu H.-K., Li L. (2020). Transition metal based battery-type electrodes in hybrid supercapacitors: a review. *Energy Storage Mater.*.

[24] Grätzel M. (2003). Dye-sensitized solar cells. *J. Photochem. Photobiol. C Photochem. Rev.*.

[25] Jošt M., Kegelmann L., Korte L., Albrecht S. (2020). Monolithic perovskite tandem solar cells: a review of the present status and advanced characterization methods toward 30% efficiency. *Adv. Energy Mater.*.

[26] Babar F., Mehmood U., Asghar H. (2020). Nanostructured photoanode materials and their deposition methods for efficient and economical third generation dye-sensitized solar cells: a comprehensive review. *Renew. Sustain. Energy Rev.*.

[27] Cariou R., Benick J., Feldmann F. (2018). III–V-on-silicon solar cells reaching 33% photoconversion efficiency in two-terminal configuration. *Nat. Energy*.

[28] Ogunniyi E. O., Pienaar H. (2017). Overview of battery energy storage system advancement for renewable (photovoltaic) energy applications. *2017 International Conference on the Domestic Use of Energy (DUE)*.

[29] Ludin N. A., Mustafa N. I., Hanafiah M. M. (2018). Prospects of life cycle assessment of renewable energy from solar photovoltaic technologies: a review. *Renew. Sustain. Energy Rev.*.

[30] Gurung A., Qiao Q. (2018). Solar charging batteries: advances, challenges, and opportunities. *Joule*.

[31] Li Q., Liu Y., Guo S., Zhou H. (2017). Solar energy storage in the rechargeable batteries. *Nano Today*.

[32] Badescu V. (2003). Dynamic model of a complex system including PV cells, electric battery, electrical motor and water pump. *Energy*.

[33] Yang Z., Li L., Luo Y. (2013). An integrated device for both photoelectric conversion and energy storage based on free-standing and aligned carbon nanotube film. *J. Mater. Chem.*.

[34] Meng H., Pang S., Cui G. (2019). Photo-supercapacitors based on third-generation solar cells. *ChemSusChem*.

[35] Vega-Garita V., Ramirez-Elizondo L., Nishant N., Bauer P. (2019). Integrating a photovoltaic storage system in one device: a critical review. *Prog. Photovoltaics Res. Appl.*.

[36] K N., Rout C. S. (2021). Photo-powered integrated supercapacitors: a review on recent developments, challenges and future perspectives. *J. Mater. Chem.*.

[37] Paolella A., Vijh A., Guerfi A., Zaghib K., Faure C. (2020). Review—Li-ion photo-batteries: challenges and opportunities. *J. Electrochem. Soc.*.

[38] Fang Z., Hu X., Yu D. (2020). Integrated photo-responsive batteries for solar energy harnessing: recent advances, challenges, and opportunities. *ChemPlusChem*.

[39] Das A., Deshagani S., Kumar R., Deepa M. (2018). Bifunctional photo-supercapacitor with a new architecture converts and stores solar energy as charge. *ACS Appl. Mater. Interfaces*.

[40] Ng C. H., Lim H. N., Hayase S., Harrison I., Pandikumar A., Huang N. M. (2015). Potential active materials for photo-supercapacitor: a review. *J. Power Sources*.

[41] Poonam, Sharma K., Arora A., Tripathi S. K. (2019). Review of supercapacitors: materials and devices. *J. Energy Storage*.

[42] Li W., Jin S. (2020). Design principles and developments of integrated solar flow batteries. *Acc. Chem. Res.*.

[43] Lu P., Leung P., Su H., Yang W., Xu Q. (2021). Materials, performance, and system design for integrated solar flow batteries – a mini review. *Appl. Energy*.

[44] Cao L., Skyllas-Kazacos M., Wang D.-W. (2018). Solar redox flow batteries: mechanism, design, and measurement. *Adv. Sustainable Syst.*.

[45] Schmidt D., Hager M. D., Schubert U. S. (2016). Photo-rechargeable electric energy storage systems. *Adv. Energy Mater.*.

[46] Liu J., Pang W. K., Zhou T. (2017). Li_2_TiSiO_5_: a low potential and large capacity Ti-based anode material for Li-ion batteries. *Energy Environ. Sci.*.

[47] Huang Q., Yang J., Ng C. B., Jia C., Wang Q. (2016). A redox flow lithium battery based on the redox targeting reactions between LiFePO_4_ and iodide. *Energy Environ. Sci.*.

[48] Ma T., Zhao Q., Wang J., Pan Z., Chen J. (2016). A sulfur heterocyclic quinone cathode and a multifunctional binder for a high-performance rechargeable lithium-ion battery. *Angew. Chem. Int. Ed.*.

[49] Wu Y., Li C., Tian Z., Sun J. (2020). Solar-driven integrated energy systems: state of the art and challenges. *J. Power Sources*.

[50] Aurbach D., McCloskey B. D., Nazar L. F., Bruce P. G. (2016). Advances in understanding mechanisms underpinning lithium–air batteries. *Nat. Energy*.

[51] Jiao F., Bruce P. G. (2007). Mesoporous crystalline β-MnO_2_—a reversible positive electrode for rechargeable lithium batteries. *Adv. Mater.*.

[52] Cheng F., Chen J. (2012). Metal–air batteries: from oxygen reduction electrochemistry to cathode catalysts. *Chem. Soc. Rev.*.

[53] Li F., Chen J. (2017). Mechanistic evolution of aprotic lithium-oxygen batteries. *Adv. Energy Mater.*.

[54] McCloskey B. D., Scheffler R., Speidel A., Bethune D. S., Shelby R. M., Luntz A. C. (2011). On the efficacy of electrocatalysis in nonaqueous Li–O_2_ batteries. *J. Am. Chem. Soc.*.

[55] Yao X., Dong Q., Cheng Q., Wang D. (2016). Why do lithium–oxygen batteries fail: parasitic chemical reactions and their synergistic effect. *Angew. Chem. Int. Ed.*.

[56] Kang S. J., Mori T., Narizuka S., Wilcke W., Kim H.-C. (2014). Deactivation of carbon electrode for elimination of carbon dioxide evolution from rechargeable lithium–oxygen cells. *Nat. Commun.*.

[57] Liu C., Neale Z., Zheng J. (2019). Expanded hydrated vanadate for high-performance aqueous zinc-ion batteries. *Energy Environ. Sci.*.

[58] Wang N., Wan H., Duan J. (2021). A review of zinc-based battery from alkaline to acid. *Mater. Today Adv.*.

[59] Boruah B. D., Mathieson A., Wen B., Feldmann S., Dose W. M., De Volder M. (2020). Photo-rechargeable zinc-ion batteries. *Energy Environ. Sci.*.

[60] Bruce P. G., Scrosati B., Tarascon J.-M. (2008). Nanomaterials for rechargeable lithium batteries. *Angew. Chem. Int. Ed.*.

[61] Yu L., Zhou X., Lu L., Wu X., Wang F. (2020). Recent developments of nanomaterials and nanostructures for high-rate lithium ion batteries. *ChemSusChem*.

[62] Sanchez C., Belleville P., Popall M., Nicole L. (2011). Applications of advanced hybrid organic–inorganic nanomaterials: from laboratory to market. *Chem. Soc. Rev.*.

[63] Yang Y., Zhang C., Lai C. (2018). BiOX (X = Cl, Br, I) photocatalytic nanomaterials: applications for fuels and environmental management. *Adv. Colloid Interface Sci.*.

[64] Leary R., Westwood A. (2011). Carbonaceous nanomaterials for the enhancement of TiO_2_ photocatalysis. *Carbon*.

[65] Wang J., Wang Y., Zhu C., Liu B. (2022). Photoinduced rechargeable lithium-ion battery. *ACS Appl. Mater. Interfaces*.

[66] Boruah B. D., Wen B., De Volder M. (2021). Light rechargeable lithium-ion batteries using V_2_O_5_ cathodes. *Nano Lett.*.

[67] Jia C., Zhang F., She L. (2021). Ultra-large sized siloxene nanosheets as bifunctional photocatalyst for a Li-O_2_ battery with superior round-trip efficiency and extra-long durability. *Angew. Chem. Int. Ed.*.

[68] Li Z., Li M.-L., Wang X.-X., Guan D.-H., Liu W.-Q., Xu J.-J. (2020). In situ fabricated photo-electro-catalytic hybrid cathode for light-assisted lithium–CO_2_ batteries. *J. Mater. Chem.*.

[69] Liu Y., Li N., Liao K., Li Q., Ishida M., Zhou H. (2016). Lowering the charge voltage of Li–O_2_ batteries via an unmediated photoelectrochemical oxidation approach. *J. Mater. Chem.*.

[70] Jing L., Zhou W., Tian G., Fu H. (2013). Surface tuning for oxide-based nanomaterials as efficient photocatalysts. *Chem. Soc. Rev.*.

[71] Wang W., Li G., Xia D., An T., Zhao H., Wong P. K. (2017). Photocatalytic nanomaterials for solar-driven bacterial inactivation: recent progress and challenges. *Environ. Sci.: Nano*.

[72] Sharma S., Dutta V., Raizada P., Hosseini-Bandegharaei A., Singh P., Nguyen V.-H. (2021). Tailoring cadmium sulfide-based photocatalytic nanomaterials for water decontamination: a review. *Environ. Chem. Lett.*.

[73] Fang Z., Zhang Y., Hu X., Fu X., Dai L., Yu D. (2019). Tactile UV- and solar-light multi-sensing rechargeable batteries with smart self-conditioned charge and discharge. *Angew. Chem. Int. Ed.*.

[74] Yu M., McCulloch W. D., Huang Z. (2016). Solar-powered electrochemical energy storage: an alternative to solar fuels. *J. Mater. Chem.*.

[75] Zeng Q., Lai Y., Jiang L. (2020). Integrated photorechargeable energy storage system: next-generation power source driving the future. *Adv. Energy Mater.*.

[76] Luo B., Ye D., Wang L. (2017). Recent progress on integrated energy conversion and storage systems. *Adv. Sci.*.

[77] Hodes G., Manassen J., Cahen D. (1976). Photoelectrochemical energy conversion and storage using polycrystalline chalcogenide electrodes. *Nature*.

[78] Liu P., Yang H. X., Ai X. P., Li G. R., Gao X. P. (2012). A solar rechargeable battery based on polymeric charge storage electrodes. *Electrochem. Commun.*.

[79] Yu M., Ren X., Ma L., Wu Y. (2014). Integrating a redox-coupled dye-sensitized photoelectrode into a lithium–oxygen battery for photoassisted charging. *Nat. Commun.*.

[80] Lv J., Tan Y.-X., Xie J. (2018). Direct solar-to-electrochemical energy storage in a functionalized covalent organic framework. *Angew. Chem. Int. Ed.*.

[81] Sharon M., Sinha A. (1982). A rechargeable photo-electrochemical solar cell (saur viddyut kosh—III). *Int. J. Hydrogen Energy*.

[82] Wang Q., Chen H., McFarland E., Wang L. (2015). Solar rechargeable batteries based on lead–organohalide electrolyte. *Adv. Energy Mater.*.

[83] Paolella A., Faure C., Bertoni G. (2017). Light-assisted delithiation of lithium iron phosphate nanocrystals towards photo-rechargeable lithium ion batteries. *Nat. Commun.*.

[84] Zhu Z., Shi X., Fan G., Li F., Chen J. (2019). Photo-energy conversion and storage in an aprotic Li-O_2_ battery. *Angew. Chem. Int. Ed.*.

[85] Gong H., Xue H., Gao B. (2020). Introduction of photo electrochemical water-oxidation mechanism into hybrid lithium–oxygen batteries. *Energy Storage Mater.*.

[86] Liu X., Yuan Y., Liu J. (2019). Utilizing solar energy to improve the oxygen evolution reaction kinetics in zinc–air battery. *Nat. Commun.*.

[87] Dunn B., Kamath H., Tarascon J.-M. (2011). Electrical energy storage for the grid: a battery of choices. *Science*.

[88] Wei X., Pan W., Duan W. (2017). Materials and systems for organic redox flow batteries: status and challenges. *ACS Energy Lett.*.

[89] Liu P., Cao Y.-l., Li G.-R., Gao X.-P., Ai X.-P., Yang H.-X. (2013). A solar rechargeable flow battery based on photoregeneration of two soluble redox couples. *ChemSusChem*.

[90] Yan N. F., Li G. R., Gao X. P. (2013). Solar rechargeable redox flow battery based on Li_2_WO_4_/LiI couples in dual-phase electrolytes. *J. Mater. Chem.*.

[91] Fu H.-C., Li W., Yang Y. (2021). An efficient and stable solar flow battery enabled by a single-junction GaAs photoelectrode. *Nat. Commun.*.

[92] Li W., Zheng J., Hu B. (2020). High-performance solar flow battery powered by a perovskite/silicon tandem solar cell. *Nat. Mater.*.

[93] Li W., Kerr E., Goulet M. A. (2019). A long lifetime aqueous organic solar flow battery. *Adv. Energy Mater.*.

[94] Liu R., Wang J., Sun T. (2017). Silicon nanowire/polymer hybrid solar cell-supercapacitor: a self-charging power unit with a total efficiency of 10.5%. *Nano Lett.*.

[95] Hu R., Zhang H., Lu Z. (2018). Unveiling critical size of coarsened Sn nanograins for achieving high round-trip efficiency of reversible conversion reaction in lithiated SnO_2_ nanocrystals. *Nano Energy*.

[96] Xu Q., Ji Y. N., Qin L. Y. (2018). Evaluation of redox flow batteries goes beyond round-trip efficiency: a technical review. *J. Energy Storage*.

[97] Xiao J., Li Q., Bi Y. (2020). Understanding and applying coulombic efficiency in lithium metal batteries. *Nat. Energy*.

[98] Yang F., Wang D., Zhao Y., Tsui K.-L., Bae S. J. (2018). A study of the relationship between coulombic efficiency and capacity degradation of commercial lithium-ion batteries. *Energy*.

[99] Liao S., Zong X., Seger B. (2016). Integrating a dual-silicon photoelectrochemical cell into a redox flow battery for unassisted photocharging. *Nat. Commun.*.

[100] Flox C., Murcia-López S., Carretero N. M., Ros C., Morante J. R., Andreu T. (2018). Role of bismuth in the electrokinetics of silicon photocathodes for solar rechargeable vanadium redox flow batteries. *ChemSusChem*.

[101] Yu M., McCulloch W. D., Beauchamp D. R., Huang Z., Ren X., Wu Y. (2015). Aqueous lithium–iodine solar flow battery for the simultaneous conversion and storage of solar energy. *J. Am. Chem. Soc.*.

[102] Zhao W., Wang X.-F., Zheng E., Wei Y., Sanehira Y., Chen G. (2017). High capacity WO_3_ film as efficient charge collection electrode for solar rechargeable batteries. *J. Power Sources*.

[103] Mahmoudzadeh M. A., Usgaocar A. R., Giorgio J., Officer D. L., Wallace G. G., Madden J. D. W. (2016). A high energy density solar rechargeable redox battery. *J. Mater. Chem.*.

[104] Kim B.-M., Lee M.-H., Dilimon V. S. (2020). Indoor-light-energy-harvesting dye-sensitized photo-rechargeable battery. *Energy Environ. Sci.*.

[105] Fan L., Jia C., Zhu Y. G., Wang Q. (2017). Redox targeting of Prussian Blue: toward low-cost and high energy density redox flow battery and solar rechargeable battery. *ACS Energy Lett.*.

[106] Andrade T. S., Dracopoulos V., Pereira M. C., Lianos P. (2020). Unmediated photoelectrochemical charging of a Zn-air battery: the realization of the photoelectrochemical battery. *J. Electroanal. Chem.*.

[107] Kin L.-c., Liu Z., Astakhov O. (2020). Efficient area matched converter aided solar charging of lithium ion batteries using high voltage perovskite solar cells. *ACS Appl. Energy Mater.*.

[108] Hu Y., Bai Y., Luo B. (2019). A portable and efficient solar-rechargeable battery with ultrafast photo-charge/discharge rate. *Adv. Energy Mater.*.

[109] Chen P., Li G. R., Li T. T., Gao X. P. (2019). Solar-driven rechargeable lithium–sulfur battery. *Adv. Sci.*.

[110] Gurung A., Reza K. M., Mabrouk S. (2020). Rear-illuminated perovskite photorechargeable lithium battery. *Adv. Funct. Mater.*.

[111] Li Q., Li N., Ishida M., Zhou H. (2015). Saving electric energy by integrating a photoelectrode into a Li-ion battery. *J. Mater. Chem.*.

[112] Wang L., Wen L., Tong Y. (2021). Photo-rechargeable batteries and supercapacitors: critical roles of carbon-based functional materials. *Carbon Energy*.

[113] Wang Z., Chiu H. C., Paolella A., Zaghib K., Demopoulos G. P. (2019). Lithium photo-intercalation of cds-sensitized WO_3_ anode for energy storage and photoelectrochromic applications. *ChemSusChem*.

[114] Zhai T., Liu H., Li H. (2010). Centimeter-long V_2_O_5_ nanowires: from synthesis to field-emission, electrochemical, electrical transport, and photoconductive properties. *Adv. Mater.*.

[115] Liu Y., Li N., Wu S. (2015). Reducing the charging voltage of a Li–O_2_ battery to 1.9 V by incorporating a photocatalyst. *Energy Environ. Sci.*.

[116] Prabhu K., Chandiran A. K. (2020). Solar energy storage in a Cs_2_AgBiBr_6_ halide double perovskite photoelectrochemical cell. *Chem. Commun.*.

[117] Qiao Y., Liu Y., Jiang K. (2018). Boosting the cycle life of aprotic Li–O_2_ batteries via a photo-assisted hybrid Li_2_O_2_-scavenging strategy. *Small Methods*.

[118] Lim H.-D., Song H., Kim J. (2014). Superior rechargeability and efficiency of lithium–oxygen batteries: hierarchical air electrode architecture combined with a soluble catalyst. *Angew. Chem. Int. Ed.*.

[119] Li N., Wang Y., Tang D., Zhou H. (2015). Integrating a photocatalyst into a hybrid lithium–sulfur battery for direct storage of solar energy. *Angew. Chem. Int. Ed.*.

[120] Zhu D., Zhao Q., Fan G. (2019). Photoinduced oxygen reduction reaction boosts the output voltage of a zinc–air battery. *Angew. Chem. Int. Ed.*.

[121] Qian W., Xu S., Zhang X. (2021). Differences and similarities of photocatalysis and electrocatalysis in two-dimensional nanomaterials: strategies, traps, applications and challenges. *Nano-Micro Lett.*.

[122] Xu C., Ravi Anusuyadevi P., Aymonier C., Luque R., Marre S. (2019). Nanostructured materials for photocatalysis. *Chem. Soc. Rev.*.

[123] Joy J., Mathew J., George S. C. (2018). Nanomaterials for photoelectrochemical water splitting – review. *Int. J. Hydrogen Energy*.

[124] Zhong Y., Peng C., He Z. (2021). Interface engineering of heterojunction photocatalysts based on 1D nanomaterials. *Catal. Sci. Technol.*.

[125] Lee S. L., Chang C.-J. (2019). Recent progress on metal sulfide composite nanomaterials for photocatalytic hydrogen production. *Catalysts*.

[126] Nazeeruddin M. K., Baranoff E., Grätzel M. (2011). Dye-sensitized solar cells: a brief overview. *Sol. Energy*.

[127] Chen H. M., Chen C. K., Liu R.-S., Zhang L., Zhang J., Wilkinson D. P. (2012). Nano-architecture and material designs for water splitting photoelectrodes. *Chem. Soc. Rev.*.

[128] Wolcott A., Smith W. A., Kuykendall T. R., Zhao Y., Zhang J. Z. (2009). Photoelectrochemical water splitting using dense and aligned TiO_2_ nanorod arrays. *Small*.

[129] Hagfeldt A., Boschloo G., Sun L., Kloo L., Pettersson H. (2010). Dye-sensitized solar cells. *Chem. Rev.*.

[130] Hardin B. E., Snaith H. J., McGehee M. D. (2012). The renaissance of dye-sensitized solar cells. *Nat. Photonics*.

[131] Lee M.-H., Kim B.-M., Lee Y. (2021). Electrochemically induced crystallite alignment of lithium manganese oxide to improve lithium insertion kinetics for dye-sensitized photorechargeable batteries. *ACS Energy Lett.*.

[132] McCulloch W. D., Yu M., Wu Y. (2016). pH-tuning a solar redox flow battery for integrated energy conversion and storage. *ACS Energy Lett.*.

[133] Xu C., Zhang X., Duan L., Zhang X., Li X., Lü W. (2020). A photo-assisted rechargeable battery: synergy, compatibility, and stability of a TiO_2_/dye/Cu_2_S bifunctional composite electrode. *Nanoscale*.

[134] Wu J., Lan Z., Lin J. (2015). Electrolytes in dye-sensitized solar cells. *Chem. Rev.*.

[135] Li J., Ren C., Zhang L. (2022). Hybridized S cathode with N719 dye for a photo-assisted charging Li-S battery. *J. Energy Chem.*.

[136] Fang W., Xing M., Zhang J. (2017). Modifications on reduced titanium dioxide photocatalysts: a review. *J. Photochem. Photobiol. C Photochem. Rev.*.

[137] Yang Z., Choi D., Kerisit S. (2009). Nanostructures and lithium electrochemical reactivity of lithium titanites and titanium oxides: a review. *J. Power Sources*.

[138] Hoffmann M. R., Martin S. T., Choi W., Bahnemann D. W. (1995). Environmental applications of semiconductor photocatalysis. *Chem. Rev.*.

[139] Barbé C. J., Arendse F., Compte P. (1997). Nanocrystalline titanium oxide electrodes for photovoltaic applications. *J. Am. Ceram. Soc.*.

[140] Wei F., Li M., Crawford R., Zhou Y., Xiao Y. (2019). Exosome-integrated titanium oxide nanotubes for targeted bone regeneration. *Acta Biomater.*.

[141] Du G. H., Chen Q., Che R. C., Yuan Z. Y., Peng L.-M. (2001). Preparation and structure analysis of titanium oxide nanotubes. *Appl. Phys. Lett.*.

[142] Kuang D., Brillet J., Chen P. (2008). Application of highly ordered TiO_2_ nanotube arrays in flexible dye-sensitized solar cells. *ACS Nano*.

[143] Cushing S. K., Meng F., Zhang J. (2017). Effects of defects on photocatalytic activity of hydrogen-treated titanium oxide nanobelts. *ACS Catal.*.

[144] Wang C.-T., Chiu Y.-C., Wang W.-P. (2016). Synthesis of iron-doped titanium oxide nanobelts for dye-sensitized solar cells. *Mater. Lett.*.

[145] Lim J. H., Choi J. (2007). Titanium oxide nanowires originating from anodically grown nanotubes: the bamboo-splitting model. *Small*.

[146] Zhang Z., Dua R., Zhang L., Zhu H., Zhang H., Wang P. (2013). Carbon-layer-protected cuprous oxide nanowire arrays for efficient water reduction. *ACS Nano*.

[147] Zhang Y., Shen Y., Gu F., Wu M., Xie Y., Zhang J. (2009). Influence of Fe ions in characteristics and optical properties of mesoporous titanium oxide thin films. *Appl. Surf. Sci.*.

[148] Ke W., Liu Y., Wang X. (2020). Nucleation and initial stages of growth during the atomic layer deposition of titanium oxide on mesoporous Silica. *Nano Lett.*.

[149] Dahl M., Liu Y., Yin Y. (2014). Composite titanium dioxide nanomaterials. *Chem. Rev.*.

[150] Chen X., Mao S. S. (2007). Titanium dioxide nanomaterials: synthesis, properties, modifications, and applications. *Chem. Rev.*.

[151] Lee K., Mazare A., Schmuki P. (2014). One-dimensional titanium dioxide nanomaterials: nanotubes. *Chem. Rev.*.

[152] Gopinath K. P., Madhav N. V., Krishnan A., Malolan R., Rangarajan G. (2020). Present applications of titanium dioxide for the photocatalytic removal of pollutants from water: a review. *J. Environ. Manag.*.

[153] Demirci S., Sunol A. K., Sahiner N. (2020). Catalytic activity of amine functionalized titanium dioxide nanoparticles in methanolysis of sodium borohydride for hydrogen generation. *Appl. Catal. B Environ.*.

[154] Aung S. H., Zhao L., Nonomura K. (2019). Toward an alternative approach for the preparation of low-temperature titanium dioxide blocking underlayers for perovskite solar cells. *J. Mater. Chem.*.

[155] Lee D.-H., Lee B.-H., Sinha A. K. (2018). Engineering titanium dioxide nanostructures for enhanced lithium-ion storage. *J. Am. Chem. Soc.*.

[156] Calcagno G., Lotsari A., Dang A. (2020). Fast charging negative electrodes based on anatase titanium dioxide beads for highly stable Li-ion capacitors. *Mater. Today Energy*.

[157] Mamaghani A. H., Haghighat F., Lee C.-S. (2020). Role of titanium dioxide (TiO_2_) structural design/morphology in photocatalytic air purification. *Appl. Catal. B Environ.*.

[158] Subhapriya S., Gomathipriya P. (2018). Green synthesis of titanium dioxide (TiO_2_) nanoparticles by Trigonella foenum-graecum extract and its antimicrobial properties. *Microb. Pathog.*.

[159] Tseng T.-M., Huang R.-H., Huang C.-Y., Liu C.-C., Hsueh K.-L., Shieu F.-S. (2014). Carbon felt coated with titanium dioxide/carbon black composite as negative electrode for vanadium redox flow battery. *J. Electrochem. Soc.*.

[160] Tseng T.-M., Huang R.-H., Huang C.-Y., Hsueh K.-L., Shieu F.-S. (2013). Improvement of titanium dioxide addition on carbon black composite for negative electrode in vanadium redox flow battery. *J. Electrochem. Soc.*.

[161] Natarajan C., Setoguchi K., Nogami G. (1998). Preparation of a nanocrystalline titanium dioxide negative electrode for the rechargeable lithium ion battery. *Electrochim. Acta*.

[162] Balogun M.-S., Li C., Zeng Y. (2014). Titanium dioxide@titanium nitride nanowires on carbon cloth with remarkable rate capability for flexible lithium-ion batteries. *J. Power Sources*.

[163] Nguyen O., Courtin E., Sauvage F., Krins N., Sanchez C., Laberty-Robert C. (2017). Shedding light on the light-driven lithium ion de-insertion reaction: towards the design of a photo-rechargeable battery. *J. Mater. Chem.*.

[164] Usui H., Miyamoto O., Nomiyama T., Horie Y., Miyazaki T. (2005). Photo-rechargeability of TiO_2_ film electrodes prepared by pulsed laser deposition. *Sol. Energy Mater. Sol. Cell.*.

[165] Andriamiadamanana C., Sagaidak I., Bouteau G., Davoisne C., Laberty-Robert C., Sauvage F. (2018). Light-Induced charge separation in mixed electronic/ionic semiconductor driving lithium-ion transfer for photo-rechargeable electrode. *Adv. Sustainable Syst.*.

[166] Fang Y.-H., Ma S.-C., Liu Z.-P. (2019). 3-D tunnel TiO_2_ crystal phase as a fast charging lithium battery anode from stochastic surface walking-based material screening. *J. Phys. Chem. C*.

[167] Dahlman C. J., Heo S., Zhang Y. (2021). Dynamics of lithium insertion in electrochromic titanium dioxide nanocrystal ensembles. *J. Am. Chem. Soc.*.

[168] Wei Z., Shen Y., Liu D. (2016). Geometry-enhanced ultra-long TiO_2_ nanobelts in an all-vanadium photoelectrochemical cell for efficient storage of solar energy. *Nano Energy*.

[169] Jang H. D., Kim S.-K., Kim S.-J. (2001). Effect of particle size and phase composition of titanium dioxide nanoparticles on the photocatalytic properties. *J. Nanoparticle Res.*.

[170] Giammar D. E., Maus C. J., Xie L. (2006). Effects of particle size and crystalline phase on lead adsorption to titanium dioxide nanoparticles. *Environ. Eng. Sci.*.

[171] Pei D.-N., Gong L., Zhang A.-Y. (2015). Defective titanium dioxide single crystals exposed by high-energy {001} facets for efficient oxygen reduction. *Nat. Commun.*.

[172] Zheng R., Shu C., Hou Z. (2019). In situ fabricating oxygen vacancy-rich TiO_2_ nanoparticles via utilizing thermodynamically metastable Ti atoms on Ti_3_C_2_T_x_ MXene nanosheet surface to boost electrocatalytic activity for high-performance Li–O_2_ batteries. *ACS Appl. Mater. Interfaces*.

[173] Ge J., Du G., Kalam A. (2021). Oxygen vacancy-rich black TiO_2_ nanoparticles as a highly efficient catalyst for Li–O_2_ batteries. *Ceram. Int.*.

[174] Han J., Lee S., Youn C., Lee J., Kim Y., Choi T. (2020). Hybrid photoelectrochemical-rechargeable seawater battery for efficient solar energy storage systems. *Electrochim. Acta*.

[175] Mathews N. R., Morales E. R., Cortés-Jacome M. A., Toledo Antonio J. A. (2009). TiO_2_ thin films – influence of annealing temperature on structural, optical and photocatalytic properties. *Sol. Energy*.

[176] Wang Y., Zhu C., Zuo G. (2020). 0D/2D Co_3_O_4_/TiO_2_ Z-scheme heterojunction for boosted photocatalytic degradation and mechanism investigation. *Appl. Catal. B Environ.*.

[177] Yun H.-S., Park B. w., Choi Y. C., Im J., Shin T. J., Seok S. I. (2019). Efficient nanostructured TiO_2_/SnS heterojunction solar cells. *Adv. Energy Mater.*.

[178] Sun B., Chen Y., Tao L. (2019). Nanorod Array of SnO_2_ Quantum Dot Interspersed Multiphase TiO_2_ heterojunctions with highly photocatalytic water splitting and self-rechargeable battery-like applications. *ACS Appl. Mater. Interfaces*.

[179] Yang X.-y., Feng X. l., Jin X. (2019). An illumination-assisted flexible self-powered energy system based on a Li–O_2_ battery. *Angew. Chem. Int. Ed.*.

[180] Li M., Wang X., Li F., Zheng L., Xu J., Yu J. (2020). A bifunctional photo-assisted Li–O_2_ battery based on a hierarchical heterostructured cathode. *Adv. Mater.*.

[181] Tong S., Luo C., Li J. (2020). Utilizing a photocatalysis process to achieve a cathode with low charging overpotential and high cycling durability for a Li-O_2_ battery. *Angew. Chem. Int. Ed.*.

[182] Macera L., Taglieri G., Daniele V., Passacantando M., D’Orazio F. (2020). Nano-sized Fe(III) oxide particles starting from an innovative and eco-friendly synthesis method. *Nanomaterials*.

[183] Al-Hakkani M. F., Gouda G. A., Hassan S. H. A. (2021). A review of green methods for phyto-fabrication of hematite (α-Fe_2_O_3_) nanoparticles and their characterization, properties, and applications. *Heliyon*.

[184] Ashraf M., Khan I., Usman M. (2020). Hematite and magnetite nanostructures for green and sustainable energy harnessing and environmental pollution control: a review. *Chem. Res. Toxicol.*.

[185] Xue Y., Wang Y. (2020). A review of the α-Fe_2_O_3_ (hematite) nanotube structure: recent advances in synthesis, characterization, and applications. *Nanoscale*.

[186] Wu C., Yin P., Zhu X., OuYang C., Xie Y. (2006). Synthesis of hematite (α-Fe_2_O_3_) nanorods: diameter-size and shape effects on their applications in magnetism, lithium ion battery, and gas sensors. *J. Phys. Chem. B*.

[187] de Carvalho V. A. N., Luz R. A. d. S., Lima B. H., Crespilho F. N., Leite E. R., Souza F. L. (2012). Highly oriented hematite nanorods arrays for photoelectrochemical water splitting. *J. Power Sources*.

[188] Zhang Z., Hossain M. F., Takahashi T. (2010). Self-assembled hematite (α-Fe_2_O_3_) nanotube arrays for photoelectrocatalytic degradation of azo dye under simulated solar light irradiation. *Appl. Catal. B Environ.*.

[189] Ali G., Park Y. J., Hussain A., Cho S. O. (2019). A novel route to the formation of 3D nanoflower-like hierarchical iron oxide nanostructure. *Nanotechnology*.

[190] Sun P., Wang C., Zhou X. (2014). Cu-doped α-Fe_2_O_3_ hierarchical microcubes: synthesis and gas sensing properties. *Sensor. Actuator. B Chem.*.

[191] Grigorescu S., Lee C.-Y., Lee K. (2012). Thermal air oxidation of Fe: rapid hematite nanowire growth and photoelectrochemical water splitting performance. *Electrochem. Commun.*.

[192] Zhu C., Li C., Zheng M., Delaunay J.-J. (2015). Plasma-induced oxygen vacancies in ultrathin hematite nanoflakes promoting photoelectrochemical water oxidation. *ACS Appl. Mater. Interfaces*.

[193] Colombo C., Palumbo G., Di Iorio E. (2015). Influence of hydrothermal synthesis conditions on size, morphology and colloidal properties of Hematite nanoparticles. *Nano-Struct. Nano-Objects*.

[194] Li L., Liu C., Qiu Y., Mitsuzak N., Chen Z. (2017). The influence of the hydrothermal temperature and time on morphology and photoelectrochemical response of α-Fe_2_O_3_ photoanode. *J. Alloys Compd.*.

[195] Wedege K., Azevedo J., Khataee A., Bentien A., Mendes A. (2016). Direct solar charging of an organic–inorganic, stable, and aqueous alkaline redox flow battery with a hematite photoanode. *Angew. Chem. Int. Ed.*.

[196] Le Formal F., Pendlebury S. R., Cornuz M., Tilley S. D., Grätzel M., Durrant J. R. (2014). Back electron–hole recombination in hematite photoanodes for water Splitting. *J. Am. Chem. Soc.*.

[197] Klahr B. M., Hamann T. W. (2011). Current and voltage limiting processes in thin film hematite electrodes. *J. Phys. Chem. C*.

[198] Azevedo J., Steier L., Dias P. (2014). On the stability enhancement of cuprous oxide water splitting photocathodes by low temperature steam annealing. *Energy Environ. Sci.*.

[199] Nikiforidis G., Tajima K., Byon H. R. (2016). High energy efficiency and stability for photoassisted aqueous lithium–iodine redox batteries. *ACS Energy Lett.*.

[200] Urbain F., Tang P., Smirnov V. (2019). Multilayered hematite nanowires with thin-film silicon photovoltaics in an all-earth-abundant hybrid tandem device for solar water splitting. *ChemSusChem*.

[201] Yao Y., Sang D., Duan S., Wang Q., Liu C. (2021). Excellent optoelectronic applications and electrical transport behavior of the n-WO_3_ nanostructures/p-diamond heterojunction: a new perspective. *Nanotechnology*.

[202] Murillo-Sierra J. C., Hernández-Ramírez A., Hinojosa-Reyes L., Guzmán-Mar J. L. (2021). A review on the development of visible light-responsive WO_3_-based photocatalysts for environmental applications. *Chem. Eng. J. Adv.*.

[203] Cheng H., Klapproth M., Sagaltchik A., Li S., Thomas A. (2018). Ordered mesoporous WO_2.83_: selective reduction synthesis, exceptional localized surface plasmon resonance and enhanced hydrogen evolution reaction activity. *J. Mater. Chem.*.

[204] Guo T., Ling C., Zhang T. (2018). High-performance WO_3_−x-WSe_2_/SiO_2_/n-Si heterojunction near-infrared photodetector via a homo-doping strategy. *J. Mater. Chem. C*.

[205] Chen J., Ren Y., Hu T., Xu T., Xu Q. (2019). Fabrication and application of substoichiometric tungsten oxide with tunable localized surface plasmon resonances. *Appl. Surf. Sci.*.

[206] Tahir M. B., Nabi G., Rafique M., Khalid N. R. (2017). Nanostructured-based WO_3_ photocatalysts: recent development, activity enhancement, perspectives and applications for wastewater treatment. *Int. J. Environ. Sci. Technol.*.

[207] Singh S., Srivastava V. C., Lo S. L. (2016). Surface Modification or Doping of WO_3_ for enhancing the photocatalytic degradation of organic pollutant containing wastewaters: a review. *Mater. Sci. Forum*.

[208] Yin C., Zhu S., Zhang D. (2017). 3D nanostructured WO_3_/BiVO_4_ heterojunction derived from Papilio paris for efficient water splitting. *RSC Adv.*.

[209] Kim Y. H., Lee S. Y., Umh H. N. (2020). Directional change of interfacial electric field by carbon insertion in heterojunction system TiO_2_/WO_3_. *ACS Appl. Mater. Interfaces*.

[210] Zhang J., Lu H., Liu C., Chen C., Xin X. (2017). Porous NiO–WO_3_ heterojunction nanofibers fabricated by electrospinning with enhanced gas sensing properties. *RSC Adv.*.

[211] Li N., Fu S., Wu J. (2020). WO_3_/ZnO nanowire heterojunction as hole transport channel for building up persistent holographic fringes. *Appl. Phys. Lett.*.

[212] Lin H., Long X., An Y., Yang S. (2020). In situ growth of Fe_2_WO_6_ on WO_3_ nanosheets to fabricate heterojunction arrays for boosting solar water splitting. *J. Chem. Phys.*.

[213] Karthik Yadav P. V., Ajitha B., Reddy Y. A. K., Minnam Reddy V. R., Reddeppa M., Kim M.-D. (2021). Effect of sputter pressure on UV photodetector performance of WO_3_ thin films. *Appl. Surf. Sci.*.

[214] Sun J., Zhang S., Zhan T. (2020). A high responsivity and controllable recovery ultraviolet detector based on a WO_3_ gate AlGaN/GaN heterostructure with an integrated micro-heater. *J. Mater. Chem. C*.

[215] Dutta V., Sharma S., Raizada P. (2021). An overview on WO_3_ based photocatalyst for environmental remediation. *J. Environ. Chem. Eng.*.

[216] Tahir M. B., Ali S., Rizwan M. (2019). A review on remediation of harmful dyes through visible light-driven WO_3_ photocatalytic nanomaterials. *Int. J. Environ. Sci. Technol.*.

[217] Wang Y., Tian W., Chen C., Xu W., Li L. (2019). Tungsten trioxide nanostructures for photoelectrochemical water splitting: material engineering and charge carrier dynamic manipulation. *Adv. Funct. Mater.*.

[218] Wei Z., Xu L., Peng S., Zhou Q. (2020). Application of WO_3_ hierarchical structures for the detection of dissolved gases in transformer oil: a mini review. *Front. Chem.*.

[219] Dai J., Li Y., Ruan H. (2021). Fiber optical hydrogen sensor based on WO_3_-Pd_2_Pt-Pt nanocomposite films. *Nanomaterials*.

[220] Feng Y., Xue H., Wang T. (2019). Enhanced Li_2_O_2_ decomposition in rechargeable Li–O_2_ battery by incorporating WO_3_ nanowire array photocatalyst. *ACS Sustain. Chem. Eng.*.

[221] Seifollahi Bazarjani M., Hojamberdiev M., Morita K. (2013). Visible light photocatalysis with c-WO_3_–x/WO_3_×H_2_O nanoheterostructures in situ formed in mesoporous polycarbosilane-siloxane polymer. *J. Am. Chem. Soc.*.

[222] Xi G., Ouyang S., Li P. (2012). Ultrathin W_18_O_49_ nanowires with diameters below 1 nm: synthesis, near-infrared absorption, photoluminescence, and photochemical reduction of carbon dioxide. *Angew. Chem. Int. Ed.*.

[223] Xue H., Wang T., Feng Y. (2020). Efficient separation of photoexcited carriers in a g-C_3_N_4_-decorated WO_3_ nanowire array heterojunction as the cathode of a rechargeable Li–O_2_ battery. *Nanoscale*.

[224] Liu M., Su B., Tang Y., Jiang X., Yu A. (2017). Recent advances in nanostructured vanadium oxides and composites for energy conversion. *Adv. Energy Mater.*.

[225] Lee S., Ivanov I. N., Keum J. K., Lee H. N. (2016). Epitaxial stabilization and phase instability of VO_2_ polymorphs. *Sci. Rep.*.

[226] Shen T. F. R., Lai M.-H., Yang T. C.-K., Fu I.-P., Liang N.-Y., Chen W.-T. (2012). Photocatalytic production of hydrogen by vanadium oxides under visible light irradiation. *J. Taiwan Inst. Chem. Eng.*.

[227] Mattelaer F., Geryl K., Rampelberg G., Dendooven J., Detavernier C. (2017). Amorphous and crystalline vanadium oxides as high-energy and high-power cathodes for three-dimensional thin-film lithium ion batteries. *ACS Appl. Mater. Interfaces*.

[228] Wang J., Yao S., Lin W. (2015). Improving the electrochemical properties of high-voltage lithium nickel manganese oxide by surface coating with vanadium oxides for lithium ion batteries. *J. Power Sources*.

[229] Zhao H., Pan L., Xing S., Luo J., Xu J. (2013). Vanadium oxides–reduced graphene oxide composite for lithium-ion batteries and supercapacitors with improved electrochemical performance. *J. Power Sources*.

[230] Cheng J., Wang B., Xin H. L. (2013). Self-assembled V_2_O_5_ nanosheets/reduced graphene oxide hierarchical nanocomposite as a high-performance cathode material for lithium ion batteries. *J. Mater. Chem.*.

[231] Palanisamy K., Um J. H., Jeong M., Yoon W.-S. (2016). Porous V_2_O_5_/RGO/CNT hierarchical architecture as a cathode material: emphasis on the contribution of surface lithium storage. *Sci. Rep.*.

[232] Chao D., Xia X., Liu J. (2014). A V_2_O_5_/conductive-polymer core/shell nanobelt array on three-dimensional graphite foam: a high-rate, ultrastable, and freestanding cathode for lithium-ion batteries. *Adv. Mater.*.

[233] Tolhurst T. M., Leedahl B., Andrews J. L., Banerjee S., Moewes A. (2017). The electronic structure of ε′-V_2_O_5_: an expanded band gap in a double-layered polymorph with increased interlayer separation. *J. Mater. Chem.*.

[234] Deka Boruah B., Mathieson A., Park S. K. (2021). Vanadium dioxide cathodes for high-rate photo-rechargeable zinc-ion batteries. *Adv. Energy Mater.*.

[235] Li Z., Ganapathy S., Xu Y., Zhou Z., Sarilar M., Wagemaker M. (2019). Mechanistic insight into the electrochemical performance of Zn/VO_2_ batteries with an aqueous ZnSO_4_ electrolyte. *Adv. Energy Mater.*.

[236] Chen L., Ruan Y., Zhang G. (2019). Ultrastable and high-performance Zn/VO_2_ battery based on a reversible single-phase reaction. *Chem. Mater.*.

[237] Ding J., Du Z., Gu L. (2018). Ultrafast Zn^2+^ intercalation and deintercalation in vanadium dioxide. *Adv. Mater.*.

[238] Guan S., Fan Q., Shen Z., Zhao Y., Sun Y., Shi Z. (2021). Heterojunction TiO_2_@TiOF_2_ nanosheets as superior anode materials for sodium-ion batteries. *J. Mater. Chem.*.

[239] de Castro I. A., Datta R. S., Ou J. Z. (2017). Molybdenum oxides – from fundamentals to functionality. *Adv. Mater.*.

[240] Ren H., Sun S., Cui J., Li X. (2018). Synthesis, functional modifications, and diversified applications of molybdenum oxides micro-/nanocrystals: a review. *Cryst. Growth Des.*.

[241] Avigad E., Etgar L. (2018). Studying the effect of MoO_3_ in hole-conductor-free perovskite solar cells. *ACS Energy Lett.*.

[242] Zhang Y., Chen P., Wang Q. (2021). High-capacity and kinetically accelerated lithium storage in MoO_3_ enabled by oxygen vacancies and heterostructure. *Adv. Energy Mater.*.

[243] Munasinghe Arachchige H. M. M., Zappa D., Poli N., Gunawardhana N., Comini E. (2018). Gold functionalized MoO_3_ nano flakes for gas sensing applications. *Sensor. Actuator. B Chem.*.

[244] Zhu Y., Yao Y., Luo Z. (2020). Nanostructured MoO_3_ for efficient energy and environmental catalysis. *Molecules*.

[245] Sun Z., Yang C., Liu G. (2017). Largely enhanced electrochemical performance in MoO_3-x_ nanobelts formed by a “sauna reaction”: importance of oxygen vacancie*s*. *Electrochim. Acta*.

[246] Huang Q., Hu S., Zhuang J., Wang X. (2012). MoO_3–x_-based hybrids with tunable localized surface plasmon resonances: chemical oxidation driving transformation from ultrathin nanosheets to nanotubes. *Chem. Eur J.*.

[247] Arash A., Ahmed T., Govind Rajan A. (2019). Large-area synthesis of 2D MoO_3− x_ for enhanced optoelectronic applications. *2D Mater.*.

[248] Lou S. N., Sharma N., Goonetilleke D. (2017). An operando mechanistic evaluation of a solar-rechargeable sodium-ion intercalation battery. *Adv. Energy Mater.*.

[249] Lou S. N., Yap N., Scott J., Amal R., Ng Y. H. (2014). Influence of MoO_3_(110) crystalline plane on its self-charging photoelectrochemical properties. *Sci. Rep.*.

[250] Besenhard J. O., Schöllhorn R. (1976). The discharge reaction mechanism of the MoO_3_ electrode in organic electrolytes. *J. Power Sources*.

[251] Światowska-Mrowiecka J., de Diesbach S., Maurice V. (2008). Li-ion intercalation in thermal oxide thin films of MoO_3_ as studied by XPS, RBS, and NRA. *J. Phys. Chem. C*.

[252] Wang J., Liu J., Zhang B. (2017). Synergistic effect of two actions sites on cobalt oxides towards electrochemical water-oxidation. *Nano Energy*.

[253] García-Mota M., Bajdich M., Viswanathan V. (2012). Importance of correlation in determining electrocatalytic oxygen evolution activity on cobalt oxides. *J. Phys. Chem. C*.

[254] Lee J. S., Jo M. S., Saroha R. (2020). Hierarchically well-developed porous graphene nanofibers comprising N-doped graphitic C-coated cobalt oxide hollow nanospheres as anodes for high-rate Li-ion batteries. *Small*.

[255] Wang K., Wan J., Xiang Y. (2020). Recent advances and historical developments of high voltage lithium cobalt oxide materials for rechargeable Li-ion batteries. *J. Power Sources*.

[256] Tang C.-W., Wang C.-B., Chien S.-H. (2008). Characterization of cobalt oxides studied by FT-IR, Raman, TPR and TG-MS. *Thermochim. Acta*.

[257] Tomon C., Sarawutanukul S., Duangdangchote S., Krittayavathananon A., Sawangphruk M. (2019). Photoactive Zn–air batteries using spinel-type cobalt oxide as a bifunctional photocatalyst at the air cathode. *Chem. Commun.*.

[258] Theerthagiri J., Karuppasamy K., Durai G. (2018). Recent advances in metal chalcogenides (MX; X = S, Se) nanostructures for electrochemical supercapacitor applications: a brief review. *Nanomaterials*.

[259] Deng W., Chen J., Yang L. (2021). Solid solution metal chalcogenides for sodium-ion batteries: the recent advances as anodes. *Small*.

[260] Jung Y., Zhou Y., Cha J. J. (2016). Intercalation in two-dimensional transition metal chalcogenides. *Inorg. Chem. Front.*.

[261] Zhang Y., Zhang L., Lv T. a., Chu P. K., Huo K. (2020). Two-dimensional transition metal chalcogenides for alkali metal ions storage. *ChemSusChem*.

[262] Yao W., Song X., Huang C., Xu Q., Wu Q. (2013). Enhancing solar hydrogen production via modified photochemical treatment of Pt/CdS photocatalyst. *Catal. Today*.

[263] Jang J. S., Ji S. M., Bae S. W., Son H. C., Lee J. S. (2007). Optimization of CdS/TiO_2_ nano-bulk composite photocatalysts for hydrogen production from Na2S/Na_2_SO_3_ aqueous electrolyte solution under visible light (λ≥420nm). *J. Photochem. Photobiol. Chem.*.

[264] Li C., Yuan J., Han B., Jiang L., Shangguan W. (2010). TiO_2_ nanotubes incorporated with CdS for photocatalytic hydrogen production from splitting water under visible light irradiation. *Int. J. Hydrogen Energy*.

[265] Peimanifard Z., Rashid-Nadimi S. (2015). Glassy carbon/multi walled carbon nanotube/cadmium sulphide photoanode for light energy storage in vanadium photoelectrochemical cell. *J. Power Sources*.

[266] Kalyanasundaram K., Borgarello E., Duonghong D., Grätzel M. (1981). Cleavage of water by visible-light irradiation of colloidal cds solutions; inhibition of photocorrosion by RuO_2_. *Angew Chem. Int. Ed. Engl.*.

[267] Zhen W., Ning X., Yang B., Wu Y., Li Z., Lu G. (2018). The enhancement of CdS photocatalytic activity for water splitting via anti-photocorrosion by coating Ni_2_P shell and removing nascent formed oxygen with artificial gill. *Appl. Catal. B Environ.*.

[268] Yang M.-Q., Han C., Xu Y.-J. (2015). Insight into the effect of highly dispersed MoS_2_ versus layer-structured MoS_2_ on the photocorrosion and photoactivity of CdS in Graphene–CdS–MoS2 composites. *J. Phys. Chem. C*.

[269] Azevedo J., Seipp T., Burfeind J. (2016). Unbiased solar energy storage: photoelectrochemical redox flow battery. *Nano Energy*.

[270] Masudy-Panah S., Eugene Y.-J. K., Khiavi N. D., Katal R., Gong X. (2018). Aluminum-incorporated p-CuO/n-ZnO photocathode coated with nanocrystal-engineered TiO_2_ protective layer for photoelectrochemical water splitting and hydrogen generation. *J. Mater. Chem.*.

[271] Zheng J., Lyu Y., Wang R. (2018). Crystalline TiO_2_ protective layer with graded oxygen defects for efficient and stable silicon-based photocathode. *Nat. Commun.*.

[272] Wang P., Wen X., Amal R., Ng Y. H. (2015). Introducing a protective interlayer of TiO_2_ in Cu_2_O–CuO heterojunction thin film as a highly stable visible light photocathode. *RSC Adv.*.

[273] Azevedo J., Tilley S. D., Schreier M. (2016). Tin oxide as stable protective layer for composite cuprous oxide water-splitting photocathodes. *Nano Energy*.

[274] Li F., Yang J., Gao J., Liu Y., Gong Y. (2020). Enhanced photocatalytic hydrogen production of CdS embedded in cationic hydrogel. *Int. J. Hydrogen Energy*.

[275] Sun Q., Wang N., Yu J., Yu J. C. (2018). A hollow porous CdS photocatalyst. *Adv. Mater.*.

[276] Pendashteh A., Rahmanifar M. S., Kaner R. B., Mousavi M. F. (2014). Facile synthesis of nanostructured CuCo_2_O_4_ as a novel electrode material for high-rate supercapacitors. *Chem. Commun.*.

[277] Liu X., Ruan Z., Zhang L. (2021). Porous cauliflower-like molybdenum disulfide/cadmium sulfide hybrid micro/nano structure: enhanced visible light absorption ability and photocatalytic activity. *J. Colloid Interface Sci.*.

[278] Majeed I., Nadeem M. A., Al-Oufi M. (2016). On the role of metal particle size and surface coverage for photo-catalytic hydrogen production: a case study of the Au/CdS system. *Appl. Catal. B Environ.*.

[279] Antoniadou M., Daskalaki V.M., Balis N. (2011). Photocatalysis and photoelectrocatalysis using (CdS-ZnS)/TiO_2_ combined photocatalysts. *Appl. Catal. B Environ.*.

[280] Wang Z., Chiu H.-C., Paolella A., Gauvin R., Zaghib K., Demopoulos G. P. (2020). A sustainable light-chargeable two-electrode energy storage system based on aqueous sodium-ion photo-intercalation. *Sustain. Energy Fuels*.

[281] Sethuraman V. A., Weidner J. W. (2010). Analysis of sulfur poisoning on a PEM fuel cell electrode. *Electrochim. Acta*.

[282] Faber M. S., Park K., Cabán-Acevedo M., Santra P. K., Jin S. (2013). Earth-abundant cobalt pyrite (CoS_2_) thin film on glass as a robust, high-performance counter electrode for quantum dot-sensitized solar cells. *J. Phys. Chem. Lett.*.

[283] Veeramani V., Chen Y.-H., Wang H.-C. (2018). CdSe/ZnS QD@CNT nanocomposite photocathode for improvement on charge overpotential in photoelectrochemical Li-O_2_ batteries. *Chem. Eng. J.*.

[284] Subila K. B., Kishore Kumar G., Shivaprasad S. M., George Thomas K. (2013). Luminescence properties of CdSe quantum dots: role of crystal structure and surface composition. *J. Phys. Chem. Lett.*.

[285] Landry M. L., Morrell T. E., Karagounis T. K., Hsia C.-H., Wang C.-Y. (2014). Simple syntheses of CdSe quantum dots. *J. Chem. Educ.*.

[286] Dabbousi B. O., Rodriguez-Viejo J., Mikulec F. V. (1997). (CdSe)ZnS core−shell quantum dots: synthesis and characterization of a size series of highly luminescent nanocrystallites. *J. Phys. Chem. B*.

[287] Mathew S., Bhardwaj B. S., Saran A. D. (2015). Effect of ZnS shell on optical properties of CdSe–ZnS core–shell quantum dots. *Opt. Mater.*.

[288] Zhu H., Song N., Lian T. (2010). Controlling charge separation and recombination rates in CdSe/ZnS type I core−shell quantum dots by shell thicknesses. *J. Am. Chem. Soc.*.

[289] Liu Y., Yi J., Qiao Y. (2018). Solar-driven efficient Li_2_O_2_ oxidation in solid-state Li-ion O_2_ batteries. *Energy Storage Mater.*.

[290] Ren C., Zhou Q., Jiang W. (2020). Investigation of germanium selenide electrodes for the integrated photo-rechargeable battery. *Int. J. Energy Res.*.

[291] Sannyal A., Zhang Z., Gao X., Jang J. (2018). Two-dimensional sheet of germanium selenide as an anode material for sodium and potassium ion batteries: first-principles simulation study. *Comput. Mater. Sci.*.

[292] Abel P. R., Klavetter K. C., Heller A., Mullins C. B. (2014). Thin nanocolumnar Ge_0.9_Se_0.1_ films are rapidly lithiated/delithiated. *J. Phys. Chem. C*.

[293] Im H. S., Lim Y. R., Cho Y. J., Park J., Cha E. H., Kang H. S. (2014). Germanium and tin selenide nanocrystals for high-capacity lithium ion batteries: comparative phase conversion of germanium and tin. *J. Phys. Chem. C*.

[294] Kim H., Son Y., Lee J. (2016). Nanocomb architecture design using germanium selenide as high-performance lithium storage material. *Chem. Mater.*.

[295] Liu X., Wu X.-Y., Chang B., Wang K.-X. (2020). Recent progress on germanium-based anodes for lithium ion batteries: efficient lithiation strategies and mechanisms. *Energy Storage Mater.*.

[296] Onodera A., Sakamoto I., Fujii Y., Mori N., Sugai S. (1997). Structural and electrical properties of GeSe and GeTe at high pressure. *Phys. Rev. B*.

[297] Cho Y. J., Im H. S., Kim H. S. (2013). Tetragonal phase germanium nanocrystals in lithium ion batteries. *ACS Nano*.

[298] Tian Z., Li C., Cai J. (2019). Solar-driven capacity enhancement of aqueous redox batteries with a vertically oriented tin disulfide array as both the photo-cathode and battery-anode. *Chem. Commun.*.

[299] Boruah B. D., Wen B., De Volder M. (2021). Molybdenum disulfide–zinc oxide photocathodes for photo-rechargeable zinc-ion batteries. *ACS Nano*.

[300] Toh R. J., Sofer Z., Luxa J., Sedmidubský D., Pumera M. (2017). 3R phase of MoS_2_ and WS_2_ outperforms the corresponding 2H phase for hydrogen evolution. *Chem. Commun.*.

[301] Friedman A. L., Hanbicki A. T., Perkins F. K., Jernigan G. G., Culbertson J. C., Campbell P. M. (2017). Evidence for chemical vapor induced 2H to 1T phase transition in MoX_2_ (X = Se, S) transition metal dichalcogenide films. *Sci. Rep.*.

[302] Tang Q., Jiang D.-e. (2015). Stabilization and band-gap tuning of the 1T-MoS_2_ monolayer by covalent functionalization. *Chem. Mater.*.

[303] Guan D.-H., Wang X. X., Li M. L. (2020). Light/electricity energy conversion and storage for a hierarchical porous In_2_S_3_@CNT/SS cathode towards a flexible Li-CO_2_ battery. *Angew. Chem. Int. Ed.*.

[304] Liu L., Xiang W., Zhong J. (2010). Flowerlike cubic β-In_2_S_3_ microspheres: synthesis and characterization. *J. Alloys Compd.*.

[305] Feng J., Yang Z., He S. (2018). Photocatalytic reduction of uranium(VI) under visible light with Sn-doped In_2_S_3_ microspheres. *Chemosphere*.

[306] Fu X., Wang X., Chen Z. (2010). Photocatalytic performance of tetragonal and cubic β-In_2_S_3_ for the water splitting under visible light irradiation. *Appl. Catal. B Environ.*.

[307] Miao Y.-f., Guo R.-t., Gu J.-w. (2020). Fabrication of β-In_2_S_3_/NiAl-LDH heterojunction photocatalyst with enhanced separation of charge carriers for efficient CO_2_ photocatalytic reduction. *Appl. Surf. Sci.*.

[308] Sarawutanukul S., Tomon C., Duangdangchote S., Phattharasupakun N., Sawangphruk M. (2020). Rechargeable photoactive Zn-air batteries using NiCo_2_S_4_ as an efficient bifunctional photocatalyst towards OER/ORR at the cathode. *Batteries Supercaps*.

[309] Zhu Y., Wu Z., Jing M., Yang X., Song W., Ji X. (2015). Mesoporous NiCo_2_S_4_ nanoparticles as high-performance electrode materials for supercapacitors. *J. Power Sources*.

[310] Jadhav H. S., Kalubarme R. S., Roh J.-W. (2014). Facile and cost effective synthesized mesoporous spinel NiCo_2_O_4_as catalyst for non-aqueous lithium-oxygen batteries. *J. Electrochem. Soc.*.

[311] Shinde S. K., Jalak M. B., Ghodake G. S. (2019). Chemically synthesized nanoflakes-like NiCo_2_S_4_ electrodes for high-performance supercapacitor application. *Appl. Surf. Sci.*.

[312] Li C., Soh K. C. K., Wu P. (2004). Formability of ABO_3_ perovskites. *J. Alloys Compd.*.

[313] Shamsi J., Urban A. S., Imran M., De Trizio L., Manna L. (2019). Metal halide perovskite nanocrystals: synthesis, post-synthesis modifications, and their optical properties. *Chem. Rev.*.

[314] Huang H., Pradhan B., Hofkens J., Roeffaers M. B. J., Steele J. A. (2020). Solar-driven metal halide perovskite photocatalysis: design, stability, and performance. *ACS Energy Lett.*.

[315] NREL Cell Chart (2021). ..

[316] Jeong J., Kim M., Seo J. (2021). Pseudo-halide anion engineering for α-FAPbI_3_ perovskite solar cells. *Nature*.

[317] Kostopoulou A., Vernardou D., Makri D., Brintakis K., Savva K., Stratakis E. (2020). Highly stable metal halide perovskite microcube anodes for lithium-air batteries. *J. Power Sources Advances*.

[318] Xia H.-R., Sun W.-T., Peng L.-M. (2015). Hydrothermal synthesis of organometal halide perovskites for Li-ion batteries. *Chem. Commun.*.

[319] Wang Q., Yang T., Wang H. (2019). Morphological and chemical tuning of lead halide perovskite mesocrystals as long-life anode materials in lithium-ion batteries. *CrystEngComm*.

[320] Mathieson A., Rahil M., Zhang Y. (2021). Ruddlesden Popper 2D perovskites as Li-ion battery electrodes. *Mater. Adv.*.

[321] Ahmad S., George C., Beesley D. J., Baumberg J. J., De Volder M. (2018). Photo-rechargeable organo-halide perovskite batteries. *Nano Lett.*.

[322] Chen P., Bai Y., Wang S., Lyu M., Yun J. H., Wang L. (2018). In situ growth of 2D perovskite capping layer for stable and efficient perovskite solar cells. *Adv. Funct. Mater.*.

[323] Kim E.-B., Akhtar M. S., Shin H.-S., Ameen S., Nazeeruddin M. K. (2021). A review on two-dimensional (2D) and 2D-3D multidimensional perovskite solar cells: perovskites structures, stability, and photovoltaic performances. *J. Photochem. Photobiol. C Photochem. Rev.*.

[324] Dawson J. A., Naylor A. J., Eames C. (2017). Mechanisms of lithium intercalation and conversion processes in organic–inorganic halide perovskites. *ACS Energy Lett.*.

[325] He M., Zhang L., Li J. (2021). Theoretical investigation on interactions between lithium ions and two-dimensional halide perovskite for solar-rechargeable batteries. *Appl. Surf. Sci.*.

[326] Martos M., Morales J., Sánchez L. (2003). Lead-based systems as suitable anode materials for Li-ion batteries. *Electrochim. Acta*.

[327] Tewari N., Shivarudraiah S. B., Halpert J. E. (2021). Photorechargeable lead-free perovskite lithium-ion batteries using hexagonal Cs_3_Bi_2_I_9_ nanosheets. *Nano Lett.*.

[328] Wu G., Li P., Zhu C. (2017). Amorphous titanium oxide passivated lithium titanium phosphate electrode for high stable aqueous lithium ion batteries with oxygen tolerance. *Electrochim. Acta*.

[329] Chen L., Cao L., Ji X. (2020). Enabling safe aqueous lithium ion open batteries by suppressing oxygen reduction reaction. *Nat. Commun.*.

[330] Chen Y., Wang C. (2020). Designing high performance organic batteries. *Acc. Chem. Res.*.

[331] Xu Y., Zhou M., Lei Y. (2018). Organic materials for rechargeable sodium-ion batteries. *Mater. Today*.

[332] Xie J., Zhang Q. (2020). Recent progress in aqueous monovalent-ion batteries with organic materials as promising electrodes. *Mater. Today Energy*.

[333] Xie J., Zhang Q. (2019). Recent progress in multivalent metal (Mg, Zn, Ca, and Al) and metal-ion rechargeable batteries with organic materials as promising electrodes. *Small*.

[334] Zhang B., He L., Yao T. (2019). Simultaneous photoelectrocatalytic water oxidation and oxygen reduction for solar electricity production in alkaline solution. *ChemSusChem*.

[335] Lv Q., Zhu Z., Zhao S. (2021). Semiconducting metal–organic polymer nanosheets for a photoinvolved Li–O_2_ battery under visible light. *J. Am. Chem. Soc.*.

[336] Kato K., Puthirath A. B., Mojibpour A. (2021). Light-assisted rechargeable lithium batteries: organic molecules for simultaneous energy harvesting and storage. *Nano Lett.*.

[337] Miroshnikov M., Kato K., Babu G. (2019). Made from henna! a fast-charging, high-capacity, and recyclable tetrakislawsone cathode material for lithium ion batteries. *ACS Sustain. Chem. Eng.*.

[338] Wang X., Maeda K., Thomas A. (2009). A metal-free polymeric photocatalyst for hydrogen production from water under visible light. *Nat. Mater.*.

[339] Zhang Q., Liu X., Chaker M., Ma D. (2021). Advancing graphitic carbon nitride-based photocatalysts toward broadband solar energy harvesting. *ACS Mater. Lett.*.

[340] Fu J., Yu J., Jiang C., Cheng B. (2018). g-C_3_N_4_-based heterostructured photocatalysts. *Adv. Energy Mater.*.

[341] Ong W.-J., Tan L.-L., Ng Y. H., Yong S.-T., Chai S.-P. (2016). Graphitic Carbon Nitride (g-C_3_N_4_)-based photocatalysts for artificial photosynthesis and environmental remediation: are we a step closer to achieving sustainability?. *Chem. Rev.*.

[342] Wang Y., Liu L., Ma T., Zhang Y., Huang H. (2021). 2D graphitic carbon nitride for energy conversion and storage. *Adv. Funct. Mater.*.

[343] Yoon Y., Lee M., Kim S. K. (2018). A strategy for synthesis of carbon nitride induced chemically doped 2D MXene for high-performance supercapacitor electrodes. *Adv. Energy Mater.*.

[344] McCloskey B. D., Speidel A., Scheffler R. (2012). Twin problems of interfacial carbonate formation in nonaqueous Li–O_2_ batteries. *J. Phys. Chem. Lett.*.

[345] Zhu Z., Y Ni, Q Lv Surface plasmon mediates the visible light–responsive lithium–oxygen battery with Au nanoparticles on defective carbon nitride. *Proc. Natl. Acad. Sci. Unit. States Am.*.

[346] Lau V. W.-h., Klose D., Kasap H. (2017). Dark photocatalysis: storage of solar energy in carbon nitride for time-delayed hydrogen generation. *Angew. Chem. Int. Ed.*.

[347] Schlomberg H., Kröger J., Savasci G. (2019). Structural insights into poly(heptazine imides): a light-storing carbon nitride material for dark photocatalysis. *Chem. Mater.*.

[348] Kröger J., Jiménez-Solano A., Savasci G. (2021). Interfacial engineering for improved photocatalysis in a charge storing 2D carbon nitride: melamine functionalized poly(heptazine imide). *Adv. Energy Mater.*.

[349] Thimmappa R., Paswan B., Gaikwad P. (2015). Chemically chargeable photo battery. *J. Phys. Chem. C*.

[350] Zukalova M., Prochazka J., Bastl Z. (2010). Facile conversion of electrospun TiO_2_ into titanium nitride/oxynitride fibers. *Chem. Mater.*.

[351] Grosso S., Latu-Romain L., Berthomé G., Renou G., Le Coz T., Mantel M. (2017). Titanium and titanium nitride thin films grown by dc reactive magnetron sputtering physical vapor deposition in a continuous mode on stainless steel wires: chemical, morphological and structural investigations. *Surf. Coating. Technol.*.

[352] Lv J., Abbas S. C., Huang Y. (2018). A photo-responsive bifunctional electrocatalyst for oxygen reduction and evolution reactions. *Nano Energy*.

[353] Wang C., Ding T., Sun Y., Zhou X., Liu Y., Yang Q. (2015). Ni12P5 nanoparticles decorated on carbon nanotubes with enhanced electrocatalytic and lithium storage properties. *Nanoscale*.

[354] Friedl J., Bauer C. M., Rinaldi A., Stimming U. (2013). Electron transfer kinetics of the VO_2_+/VO_2_+ – reaction on multi-walled carbon nanotubes. *Carbon*.

[355] Wang J., Li K., Zhong H.-x. (2015). Synergistic effect between metal–nitrogen–carbon sheets and NiO nanoparticles for enhanced electrochemical water-oxidation performance. *Angew. Chem. Int. Ed.*.

[356] Song H., Wang S., Song X. (2020). Solar-driven all-solid-state lithium–air batteries operating at extreme low temperatures. *Energy Environ. Sci.*.

[357] Lalisse A., Tessier G., Plain J., Baffou G. (2015). Quantifying the efficiency of plasmonic materials for near-field enhancement and photothermal conversion. *J. Phys. Chem. C*.

[358] Novoa-Cid M., Baldovi H. G. (2020). Study of the photothermal catalytic mechanism of CO_2_ reduction to CH_4_ by ruthenium nanoparticles supported on titanate nanotubes. *Nanomaterials*.

[359] Li S., Wang H., Li D. (2016). Siloxene nanosheets: a metal-free semiconductor for water splitting. *J. Mater. Chem.*.

[360] Krishnamoorthy K., Pazhamalai P., Kim S.-J. (2018). Two-dimensional siloxene nanosheets: novel high-performance supercapacitor electrode materials. *Energy Environ. Sci.*.

[361] Mei J., Liao T., Sun Z. (2018). Two-dimensional metal oxide nanosheets for rechargeable batteries. *J. Energy Chem.*.

[362] Podjaski F., Kröger J., Lotsch B. V. (2018). Toward an aqueous solar battery: direct electrochemical storage of solar energy in carbon nitrides. *Adv. Mater.*.

[363] Suntivich J., Gasteiger H. A., Yabuuchi N., Nakanishi H., Goodenough J. B., Shao-Horn Y. (2011). Design principles for oxygen-reduction activity on perovskite oxide catalysts for fuel cells and metal–air batteries. *Nat. Chem.*.

[364] Kim Y., Seo H., Kim E., Kim J., Seo I. (2020). Development of a self-charging lithium-ion battery using perovskite solar cells. *Nanomaterials*.

[365] Tan P., Liu M., Shao Z., Ni M. (2017). Recent advances in perovskite oxides as electrode materials for nonaqueous lithium–oxygen batteries. *Adv. Energy Mater.*.

[366] Tan P., Xiao X., Dai Y., Cheng C., Ni M. (2020). Photo-assisted non-aqueous lithium-oxygen batteries: progress and prospects. *Renew. Sustain. Energy Rev.*.

[367] Toe C. Y., Scott J., Amal R., Ng Y. H. (2019). Recent advances in suppressing the photocorrosion of cuprous oxide for photocatalytic and photoelectrochemical energy conversion. *J. Photochem. Photobiol. C Photochem. Rev.*.

[368] Ning X., Lu G. (2020). Photocorrosion inhibition of CdS-based catalysts for photocatalytic overall water splitting. *Nanoscale*.

[369] Weng B., Qi M.-Y., Han C., Tang Z.-R., Xu Y.-J. (2019). Photocorrosion inhibition of semiconductor-based photocatalysts: basic principle, current development, and future perspective. *ACS Catal.*.

[370] You D., Xu C., Wang X. (2020). A core@dual-shell nanorod array with a cascading band configuration for enhanced photocatalytic properties and anti-photocorrosion. *J. Mater. Chem.*.

[371] Franz S., Arab H., Chiarello G. L., Bestetti M., Selli E. (2020). Single-step preparation of large area TiO_2_ photoelectrodes for water splitting. *Adv. Energy Mater.*.

[372] Yang W., Prabhakar R. R., Tan J., Tilley S. D., Moon J. (2019). Strategies for enhancing the photocurrent, photovoltage, and stability of photoelectrodes for photoelectrochemical water splitting. *Chem. Soc. Rev.*.

[373] Liu N., Albu S. P., Lee K., So S., Schmuki P. (2012). Water annealing and other low temperature treatments of anodic TiO_2_ nanotubes: a comparison of properties and efficiencies in dye sensitized solar cells and for water splitting. *Electrochim. Acta*.

[374] Chan C. K., Peng H., Twesten R. D., Jarausch K., Zhang X. F., Cui Y. (2007). Fast, completely reversible Li insertion in vanadium pentoxide nanoribbons. *Nano Lett.*.

[375] Park M.-S., Wang G.-X., Kang Y.-M., Wexler D., Dou S.-X., Liu H.-K. (2007). Preparation and electrochemical properties of SnO_2_ nanowires for application in lithium-ion batteries. *Angew. Chem. Int. Ed.*.

[376] Nam Ki T., Kim D.-W., Yoo P. J. (2006). Virus-enabled synthesis and assembly of nanowires for lithium ion battery electrodes. *Science*.

[377] Ghosh S., Raj C. R. (2010). Facile in situ synthesis of multiwall carbon nanotube supported flowerlike Pt nanostructures: an efficient electrocatalyst for fuel cell application. *J. Phys. Chem. C*.

[378] Feng X., Zhu K., Frank A. J., Grimes C. A., Mallouk T. E. (2012). Rapid charge transport in dye-sensitized solar cells made from vertically aligned single-crystal rutile TiO_2_ nanowires. *Angew. Chem. Int. Ed.*.

[379] Chou S.-L., Wang J.-Z., Sun J.-Z. (2008). High capacity, safety, and enhanced cyclability of lithium metal battery using a V_2_O_5_ nanomaterial cathode and room temperature ionic liquid electrolyte. *Chem. Mater.*.

[380] Li H., He P., Wang Y., Hosono E., Zhou H. (2011). High-surface vanadium oxides with large capacities for lithium-ion batteries: from hydrated aerogel to nanocrystalline VO_2_(B), V_6_O_13_ and V_2_O_5_. *J. Mater. Chem.*.

[381] Wang H. G., Ma D.-l., Huang Y., Zhang X.-b. (2012). Electrospun V_2_O_5_ nanostructures with controllable morphology as high-performance cathode materials for lithium-ion batteries. *Chemistry*.

[382] Xie J., Li B. Q., Peng H. J. (2019). Implanting atomic cobalt within mesoporous carbon toward highly stable lithium–sulfur batteries. *Adv. Mater.*.

[383] Hou J., Tu X., Wu X. (2020). Remarkable cycling durability of lithium-sulfur batteries with interconnected mesoporous hollow carbon nanospheres as high sulfur content host. *Chem. Eng. J.*.

[384] Wang J., Liao L., Lee H. R. (2019). Surface-engineered mesoporous silicon microparticles as high-Coulombic-efficiency anodes for lithium-ion batteries. *Nano Energy*.

[385] Oh S. M., Patil S. B., Jin X., Hwang S.-J. (2018). Recent applications of 2D inorganic nanosheets for emerging energy storage system. *Chem. Eur J.*.

[386] Gu T.-H., Kwon N. H., Lee K.-G., Jin X., Hwang S.-J. (2020). 2D inorganic nanosheets as versatile building blocks for hybrid electrode materials for supercapacitor. *Coord. Chem. Rev.*.

[387] Chen J. S., Lou X. W. (2012). SnO_2_ and TiO_2_ nanosheets for lithium-ion batteries. *Mater. Today*.

[388] Yang X.-F., Wang A., Qiao B., Li J., Liu J., Zhang T. (2013). Single-atom catalysts: a new Frontier in heterogeneous catalysis. *Acc. Chem. Res.*.

[389] Wang A., Li J., Zhang T. (2018). Heterogeneous single-atom catalysis. *Nat. Rev. Chem.*.

[390] Chen Y., Ji S., Chen C., Peng Q., Wang D., Li Y. (2018). Single-atom catalysts: synthetic strategies and electrochemical applications. *Joule*.

[391] Sick N., Bröring S., Figgemeier E. (2018). Start-ups as technology life cycle indicator for the early stage of application: an analysis of the battery value chain. *J. Clean. Prod.*.

[392] Li X., Chalvatzis K. J., Stephanides P. (2018). Innovative energy islands: life-cycle cost-benefit analysis for battery energy storage. *Sustainability*.

[393] Sun Y.-K. (2021). An experimental checklist for reporting battery performances. *ACS Energy Lett.*.

